# Protective effectiveness of previous SARS-CoV-2 infection and hybrid immunity against the omicron variant and severe disease: a systematic review and meta-regression

**DOI:** 10.1016/S1473-3099(22)00801-5

**Published:** 2023-05

**Authors:** Niklas Bobrovitz, Harriet Ware, Xiaomeng Ma, Zihan Li, Reza Hosseini, Christian Cao, Anabel Selemon, Mairead Whelan, Zahra Premji, Hanane Issa, Brianna Cheng, Laith J Abu Raddad, David L Buckeridge, Maria D Van Kerkhove, Vanessa Piechotta, Melissa M Higdon, Annelies Wilder-Smith, Isabel Bergeri, Daniel R Feikin, Rahul K Arora, Minal K Patel, Lorenzo Subissi

**Affiliations:** a Temerty Faculty of Medicine, University of Toronto, Toronto, ON, Canada; b Institute of Health Policy Management and Evaluation, University of Toronto, Toronto, ON, Canada; c Department of Critical Care Medicine, University of Calgary, Calgary, AB, Canada; d Centre for Health Informatics, Cumming School of Medicine, University of Calgary, Calgary, AB, Canada; e Department of Bioengineering, University of California, Berkeley, CA, USA; f School of Population and Public Health, University of British Columbia, Vancouver, BC, Canada; g Libraries, University of Victoria, Victoria, BC, Canada; h Institute of Health Informatics, University College London, London, UK; i Infectious Disease Epidemiology Group, Weill Cornell Medicine–Qatar, Cornell University, Doha, Qatar; j Department of Epidemiology and Biostatistics, School of Population and Global Health, McGill University, Montreal, QC, Canada; k Health Emergencies Programme, World Health Organization, Geneva, Switzerland; l Department of Infectious Disease Epidemiology, Robert Koch Institute, Berlin, Germany; m International Vaccine Access Center, Department of International Health, John Hopkins Bloomberg School of Public Health, Johns Hopkins University, Baltimore, MD, USA; n Department of Immunizations, Vaccines and Biologicals, World Health Organization, Geneva, Switzerland; o Heidelberg Institute of Global Health, University of Heidelberg, Germany; p Institute of Biomedical Engineering, University of Oxford, Oxford, UK

## Abstract

**Background:**

The global surge in the omicron (B.1.1.529) variant has resulted in many individuals with hybrid immunity (immunity developed through a combination of SARS-CoV-2 infection and vaccination). We aimed to systematically review the magnitude and duration of the protective effectiveness of previous SARS-CoV-2 infection and hybrid immunity against infection and severe disease caused by the omicron variant.

**Methods:**

For this systematic review and meta-regression, we searched for cohort, cross-sectional, and case–control studies in MEDLINE, Embase, Web of Science, ClinicalTrials.gov, the Cochrane Central Register of Controlled Trials, the WHO COVID-19 database, and Europe PubMed Central from Jan 1, 2020, to June 1, 2022, using keywords related to SARS-CoV-2, reinfection, protective effectiveness, previous infection, presence of antibodies, and hybrid immunity. The main outcomes were the protective effectiveness against reinfection and against hospital admission or severe disease of hybrid immunity, hybrid immunity relative to previous infection alone, hybrid immunity relative to previous vaccination alone, and hybrid immunity relative to hybrid immunity with fewer vaccine doses. Risk of bias was assessed with the Risk of Bias In Non-Randomized Studies of Interventions Tool. We used log-odds random-effects meta-regression to estimate the magnitude of protection at 1-month intervals. This study was registered with PROSPERO (CRD42022318605).

**Findings:**

11 studies reporting the protective effectiveness of previous SARS-CoV-2 infection and 15 studies reporting the protective effectiveness of hybrid immunity were included. For previous infection, there were 97 estimates (27 with a moderate risk of bias and 70 with a serious risk of bias). The effectiveness of previous infection against hospital admission or severe disease was 74·6% (95% CI 63·1–83·5) at 12 months. The effectiveness of previous infection against reinfection waned to 24·7% (95% CI 16·4–35·5) at 12 months. For hybrid immunity, there were 153 estimates (78 with a moderate risk of bias and 75 with a serious risk of bias). The effectiveness of hybrid immunity against hospital admission or severe disease was 97·4% (95% CI 91·4–99·2) at 12 months with primary series vaccination and 95·3% (81·9–98·9) at 6 months with the first booster vaccination after the most recent infection or vaccination. Against reinfection, the effectiveness of hybrid immunity following primary series vaccination waned to 41·8% (95% CI 31·5–52·8) at 12 months, while the effectiveness of hybrid immunity following first booster vaccination waned to 46·5% (36·0–57·3) at 6 months.

**Interpretation:**

All estimates of protection waned within months against reinfection but remained high and sustained for hospital admission or severe disease. Individuals with hybrid immunity had the highest magnitude and durability of protection, and as a result might be able to extend the period before booster vaccinations are needed compared to individuals who have never been infected.

**Funding:**

WHO COVID-19 Solidarity Response Fund and the Coalition for Epidemic Preparedness Innovations.

## Introduction

Restricting the spread of SARS-CoV-2 infection and preventing severe COVID-19 remains a priority at the global scale. Immunological protection from SARS-CoV-2 can be induced from previous infection or vaccination.^1^, ^2^ However, estimating the magnitude and durability of this protection in the population has become a challenge because of the surge in the omicron (B.1.1.529) variant, which has resulted in many individuals with hybrid immunity (immunity developed through a combination of SARS-CoV-2 infection and vaccination), varying rates and timings of past infection and vaccination, multiple types of vaccination and numbers of doses, and variants of concern that can escape pre-existing immunity.^3^, ^4^

**Research in context**

**Evidence before this study**

The global emergence and rapid spread of the omicron (B.1.1.529) variant of SARS-CoV-2 and its subvariants, characterised by its ability to escape immunity, has required scientists and policy makers to reassess population protection against SARS-CoV-2 infection and severe disease due to the omicron variant. We searched MEDLINE (Ovid), Embase (Ovid), Web of Science (Core Collection), ClinicalTrials.gov, the Cochrane Central Register of Controlled Trials (Ovid), the WHO COVID-19 database, and Europe PubMed Central (limited to preprints) from Jan 1, 2020, to June 1, 2022, with keywords related to SARS-CoV-2, reinfection, protective effectiveness, previous infection, presence of antibodies, and hybrid immunity. We found a few systematic reviews that incorporated data on the omicron variant, but none that examined protection against the omicron variant conferred by hybrid immunity (ie, immunity gained from the combination of vaccination and previous infection), which is now widespread globally. One review reported the duration and magnitude of protection from previous infection over time; however, to the best of our knowledge, no study has systematically compared the magnitude and duration of protection from vaccination, previous infection, and hybrid immunity. A large study in Qatar reported that protection from infection or hybrid immunity against the omicron variant wanes to low levels at 15 months but is relatively stable against severe disease.

**Added value of this study**

Previous SARS-CoV-2 infection and hybrid immunity both provided greater and more sustained protection against the omicron variant than vaccination alone. Individuals with hybrid immunity had the highest magnitude and durability of protection against all outcomes; protection against severe disease remained higher than 95% until the end of available follow-up at 11 months after hybrid immunity with primary series vaccination and 4 months after hybrid immunity with booster vaccination, and was sustained at these high levels of protection in projections to 12 months after primary series hybrid immunity and 6 months after booster vaccination hybrid immunity.

**Implications of all the available evidence**

These results provide information that can be used to tailor guidance on the number and timing of SARS-CoV-2 vaccinations. At the public health level, these findings can be combined with data on local infection prevalence, vaccination rates, and their timing. In settings with high seroprevalence, scarce resources, and competing health priorities, evidence suggests that it is reasonable to focus on achieving high coverage rates with primary series vaccination among individuals who are at higher risk of poor outcomes, as this will provide a high level of protection against severe disease for at least 1 year among those with previous infection. Furthermore, given the waning protection for both infection-induced and vaccine-induced immunity against SARS-CoV-2 infection or reinfection, wider vaccination among populations (eg, mass vaccination) could be timed for rollout before periods of expected increased incidence, such as the winter season. At the individual level, these results can be combined with knowledge of a person's infection and vaccination history. A 6-month delay in a booster dose might be justified after the last infection or vaccination for individuals with a known history of previous infection and full primary series vaccination. Further follow-up data on the protective effectiveness of hybrid immunity against hospital admission or severe disease for all vaccines are needed to clarify how much waning of protection might occur with a longer duration since the last infection or vaccination. Producing estimates of protection for vaccines targeting new variants will be crucial for COVID-19 vaccination policy and decision-making bodies. Policy makers considering the use and timing of vaccinations could include the local extent of past infection, the protection conferred by previous infection or hybrid immunity, and the duration of this protection as key considerations to inform their decision making.

Systematic reviews of SARS-CoV-2 vaccine effectiveness studies have provided clarity on the durability of protection for different variants of concern.^5^, ^6^ These studies have compared protection among vaccinated individuals to that in unvaccinated individuals and compared protection between different numbers of doses. However, there are gaps in the literature on the magnitude and duration of protection conferred by previous infection, both among individuals who have not been vaccinated (ie, the effectiveness of previous infection alone) and among individuals who have been vaccinated (ie, the effectiveness of hybrid immunity). A systematic review has estimated the durability of protection conferred by previous infection.^7^ However, no systematic review has, to the best of our knowledge, estimated the durability of protection conferred by hybrid immunity or compared the durability of the different types of protection.

We aimed to systematically review the evidence for the magnitude and duration of the effectiveness of previous infection and hybrid immunity against multiple clinical outcomes of SARS-CoV-2 infection caused by the omicron variant. We also aimed to examine the comparative protection of hybrid immunity relative to previous infection only, vaccination only, and hybrid immunity with fewer vaccine doses.

## Methods

### Search strategy and selection criteria

This systematic review and meta-regression was registered with PROSPERO (CRD42022318605), conducted in alignment with the Cochrane Handbook for Systematic Reviews of Interventions,^8^ and reported according to the Preferred Reporting Items for Systematic Reviews and Meta-Analyses (PRISMA) guidelines (appendix pp 2–4).^9^

We searched MEDLINE (Ovid), Embase (Ovid), Web of Science (Core Collection), ClinicalTrials.gov, the Cochrane Central Register of Controlled Trials (Ovid), WHO COVID-19 database, and Europe PubMed Central (limited to preprints) from Jan 1, 2020, to June 1, 2022 (appendix pp 5–9). The search strategy comprised three keyword concepts: SARS-CoV-2; reinfection and protective effectiveness; and previous infection, presence of antibodies, and hybrid immunity. We also sought article recommendations from authors of previous vaccine effectiveness studies and WHO Solidarity II network investigators.

Screening was done in Covidence software with titles and abstracts and full-text manuscripts. Two reviewers independently screened studies (NB, XM, ZL, RH, CC, AS, HI, BC). Conflicts were resolved by a third reviewer (NB, XM).

Detailed inclusion and exclusion criteria are reported in the appendix (pp 10–12). We included studies examining protection against reinfection with the omicron variant, in which the exposure group was people with previous infection with any SARS-CoV-2 variant or hybrid immunity and the control group was immune-naive individuals, previously infected individuals, or previously vaccinated individuals. Infection due to the omicron variant was determined by genomic sequencing or inferred on the basis of time periods when the variants were predominant according to the GISAID (Global Initiative on Sharing Avian Influenza Data) EpiFlu database. Studies were excluded if they did not report evidence of previously confirmed SARS-CoV-2 cases or did not report the period of time between the index infection and reinfection.

### Outcome definition and comparison groups

SARS-CoV-2 reinfection was defined as a possible, probable, or confirmed reinfection case according to adapted WHO definitions (appendix p 10).^10^ Partial primary series and full primary series vaccination were defined according to the original clinical trials of the respective COVID-19 vaccines. First booster vaccination included individuals at least 7 days from receipt of the first booster dose.

Estimates of vaccine effectiveness (ie, vaccinated *vs* unvaccinated) against the omicron variant were obtained from the dataset of a systematic review and meta-regression involving 19 studies of both primary series and booster vaccination (of which 18 were primary series studies and 12 were first booster studies).^6^ This previous systematic review^6^ used similar inclusion and exclusion criteria to our analysis. We applied our meta-regression model to the dataset to project the trends of waning vaccine effectiveness, in parallel with trends for previous infection and hybrid immunity generated from data procured in our systematic review (appendix pp 12–13).

### Data analysis

Summary data were extracted from published reports by one reviewer and verified by a second reviewer (NB, XM, ZL, RH, CC, and AS). We extracted data for each outcome (infection [asymptomatic or symptomatic disease], hospital admission, or severe disease [a combination of the WHO definitions of severe, critical, or fatal COVID-19]),^11^ stratifying by age, sex, vaccine type, the variant of concern causing the index infection, and the severity of the index infection (appendix pp 10–13). Few studies reported ethnicity data, so we were unable to analyse the impact of ethnicity on our study outcomes.

Risk of bias was assessed with the Risk of Bias In Non-Randomized Studies of Interventions (ROBINS-I) Tool.^12^ Two reviewers (NB and VP) independently completed the assessments for each reported outcome. Conflicts were resolved by discussion and consensus among the two authors.

Five effect measures of protection were calculated on the basis of different comparisons of immune status: the protective effectiveness of previous infection (defined as previous infection *vs* immune naive); the protective effectiveness of hybrid immunity (defined as hybrid immunity *vs* immune naive); the comparative protective effectiveness of hybrid immunity relative to previous infection alone (defined as hybrid immunity *vs* infection); the comparative protective effectiveness of hybrid immunity relative to previous vaccination alone (defined as hybrid immunity *vs* previous vaccination); and the comparative protective effectiveness of hybrid immunity relative to hybrid immunity with fewer vaccine doses (defined as hybrid immunity with more vaccine doses *vs* hybrid immunity with fewer vaccine doses). The immunity comparisons, effect measures, and their epidemiological importance are described in the appendix (p 13). The primary outcomes were the abovementioned five measures of protection against hospital admission due to COVID-19 or severe disease at discrete timepoints. The secondary outcomes were the same five measures of protection against infection with the omicron variant at discrete timepoints. For previous infection, we used estimates starting from 2 months after the primary infection, reflecting the length of time required for a possible reinfection. For hybrid immunity, we used estimates starting from 2 months after the primary infection or at least 7 days after the most recent vaccination (the cutoff threshold varied by vaccine according to the original clinical trials; appendix pp 10–11).

Using the log-odds meta-regression model, we generated estimates of effectiveness in successive months as well as percentage-point changes in effectiveness from 3 to 6 months and from 3 to 12 months, with 95% bootstrap CIs for the changes calculated using 1000 bootstrap samples. For the protective effectiveness of hybrid immunity we predicted estimates 1 month and 2 months beyond final follow-up times, yielding estimates of protection at 12 months for hybrid immunity with primary series vaccination and 6 months for hybrid immunity with booster vaccination. For a given outcome measure, if there were insufficient data for meta-regression over time then estimates were pooled via meta-analysis (same log-odds model with no time covariate).^13^ These estimates represented an average of the timepoints reported in different studies. Modelling details are reported in the appendix (p 12).

Two sensitivity analyses were done: one analysis focused on severe disease and excluded all-cause hospital admission and the other excluded estimates with a serious risk of bias. There were insufficient data to report results stratified by age, sex, severity of the index infection, and the omicron subvariant causing reinfection.

Data were analysed with R statistical software (version 4.1.2).^14^

### Role of the funding source

The funders had no role in study design, data collection, data analysis, data interpretation, or the writing of the report.

## Results

**Figure 1:**
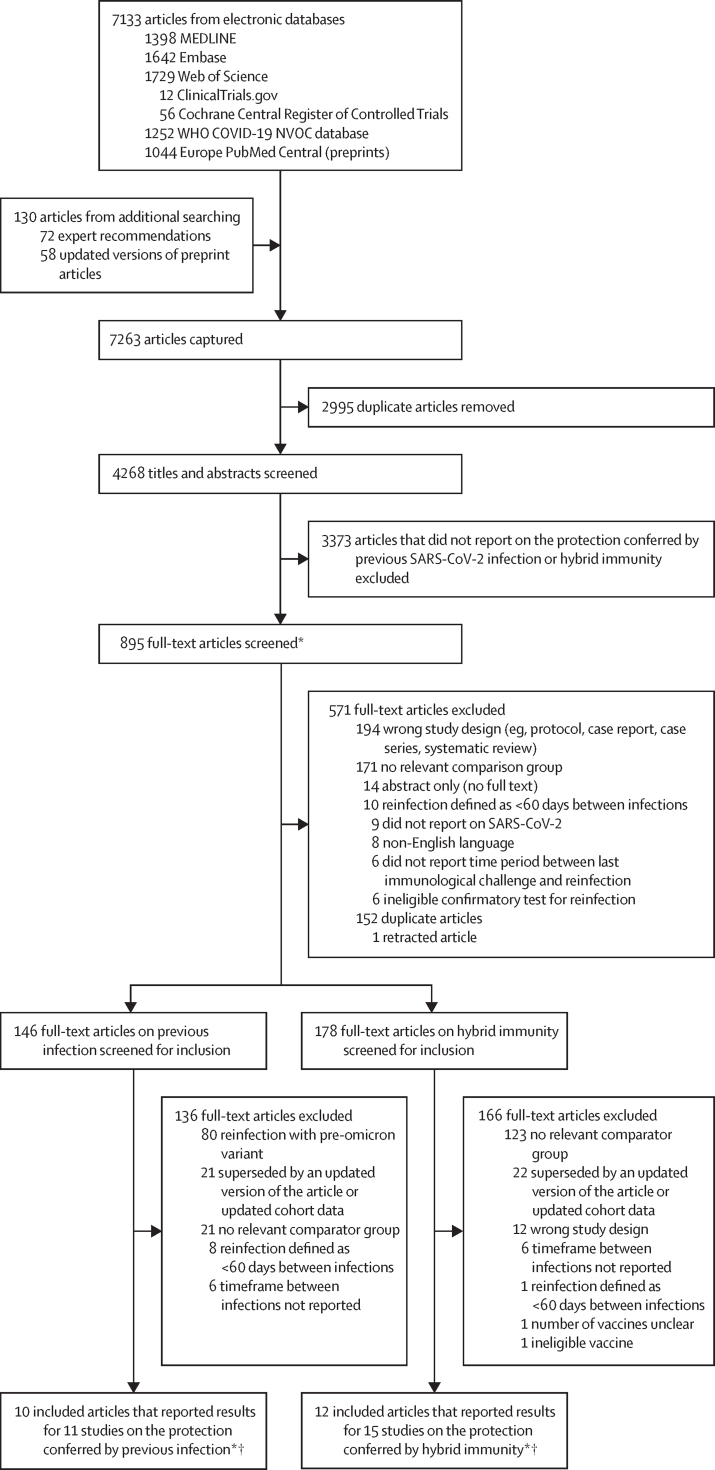
Study selection *There were two rounds of full-text screening. Studies not meeting general inclusion criteria were excluded in the first round. In the second round, articles were screened with criteria specific to the types of exposures and comparators being reported (ie, previous infection and hybrid immunity). †There were 16 unique articles: nine articles reported on one study, five articles reported on two studies (one on previous infection and one on hybrid immunity), one article reported on three studies (one on previous infection and two on hybrid immunity), and one article reported on four studies (two on previous infection and two on hybrid immunity). Seven articles reported data for previous infection and hybrid immunity.

4268 article titles and abstracts were screened and 895 underwent full-text review (figure 1). 16 unique articles reporting data for 26 studies were included in the analysis.^15^, ^16^, ^17^, ^18^, ^19^, ^20^, ^21^, ^22^, ^23^, ^24^, ^25^, ^26^, ^27^, ^28^, ^29^, ^30^ Seven articles reported data for two or more studies (at least one previous infection study and at least one hybrid immunity study), defined by multiple sets of participant inclusion and exclusion criteria or populations that resulted in unique non-pooled estimates of protection for each population. 11 studies reported on previous infection and 15 reported on hybrid immunity; seven reported on both. A summary of the characteristics of included studies is shown in table 1. Individual study characteristics are reported in the appendix (pp 14–37). For previous infection there were 97 estimates: 27 with a moderate risk of bias and 70 with a serious risk of bias. For hybrid immunity there were 153 estimates: 78 with a moderate risk of bias and 75 with a serious risk of bias. The most common reason for an increased risk of bias was incomplete adjustment for confounding factors (eg, testing frequency, comorbidity, or calendar time) and not reporting an a-priori protocol to enable ruling out reporting bias (appendix pp 38–42).

**Table 1:** Study characteristics

		**Studies focusing on previous infection**	**Studies focusing on hybrid immunity**				
		Previous infection *vs* immune naive* (n=11)	Hybrid immunity *vs* immune naive* (n=9)	Hybrid immunity *vs* previous infection* (n=7)	Hybrid immunity *vs* hybrid immunity with fewer vaccine doses* (n=4)	Hybrid immunity *vs* vaccination* (n=1)	Summary of all hybrid immunity studies (n=15)
Study design							
	Cohort	2 (18%)	0 (0%)	2 (29%)	1 (25%)	1 (100%)	3 (20%)
	Cross-sectional	1 (9%)	0 (0%)	0 (0%)	0 (0%)	0 (0%)	0 (0%)
	Test-negative design case–control	8 (73%)	7 (78%)	4 (57%)	2 (50%)	0 (40%)	9 (60%)
	Traditional case–control	0 (0%)	2 (22%)	1 (14%)	1 (25%)	0 (0%)	3 (20%)
Total sample size		294 900 (83 251–1 142 605)	317 110 (50 576–696 439)	130 073 (14 625–470 984)	75 643 (17 919–271 664)	38	130 073 (14 625–335 882)
Study population							
	General population	11 (100%)	9 (100%)	6 (86%)	3 (75%)	1 (100%)	14 (93%)
	Health-care workers	0 (0%)	0 (0%)	1 (14%)	1 (25%)	0 (0%)	1 (7%)
WHO region							
	African region	0 (0%)	0 (0%)	0 (0%)	0 (0%)	0 (0%)	0 (0%)
	Region of the Americas	4 (36%)	3 (33%)	5 (71%)	3 (75%)	0 (0%)	6 (40%)
	Eastern Mediterranean region	2 (18%)	2 (22%)	0 (0%)	0 (0%)	0 (0%)	2 (13%)
	European region	5 (45%)	4 (44%)	2 (29%)	1 (25%)	1 (100%)	7 (47%)
	South-East Asia region	0 (0%)	0 (0%)	0 (0%)	0 (0%)	0 (0%)	0 (0%)
	Western Pacific region	0 (0%)	0 (0%)	0 (0%)	0 (0%)	0 (0%)	0 (0%)
Reported the sequence of index infection and vaccination		..	4 (44%)	5 (71%)	3 (75%)	1 (100%)	9 (60%)
Reported omicron (B.1.1.529) subvariants		4 (36%)	5 (56%)	1 (14%)	1 (25%)	0 (0%)	6 (40%)
Primary infection dominant variant							
	Alpha (B.1.1.7)	1 (9%)	1 (11%)	0 (0%)	0 (0%)	0 (0%)	1 (7%)
	Delta (B.1.617.2)	3 (27%)	4 (44%)	0 (0%)	0 (0%)	0 (0%)	4 (27%)
	Mixed variant	9 (82%)	7 (78%)	7 (100%)	4 (100%)	1 (100%)	13 (87%)
Primary series vaccination for hybrid immunity†		..	9	5	3	..	12
	Inactivated: CoronaVac (Sinovac)	..	1 (11%)	1 (14%)	0 (0%)	..	1 (7%)
	mRNA: BNT162b2 (Pfizer–BioNTech)	..	1 (11%)	1 (14%)	0 (0%)	..	2 (13%)
	mRNA: mRNA-1273 (Moderna)	..	1 (11%)	0 (0%)	0 (0%)	..	1 (7%)
	mRNA: BNT162b2 (Pfizer–BioNTech) plus mRNA-1273 (Moderna)	..	2 (22%)	3 (43%)	3 (100%)	..	5 (33%)
	NRVV: Vaxzevria (Oxford–AstraZeneca)	..	1 (11%)	1 (14%)	0 (0%)	..	1 (7%)
	NRVV: Ad26.COV2.S (Janssen)	..	1 (11%)	1 (14%)	0 (0%)	..	1 (7%)
	Mixed (inactivated plus NRVV plus mRNA)‡	..	5 (56%)	4 (57%)	0 (0%)	..	8 (53%)
First booster vaccination for hybrid immunity†		..	7	5	4	1	12
	Inactivated: CoronaVac (Sinovac)	..	1 (11%)	1 (14%)	0 (0%)	0 (0%)	1 (7%)
	mRNA: BNT162b2 (Pfizer–BioNTech)	..	2 (22%)	1 (14%)	0 (0%)	0 (0%)	3 (20%)
	mRNA: BNT162b2 (Pfizer–BioNTech) plus mRNA-1273 (Moderna)	..	2 (22%)	3 (43%)	3 (75%)	1 (100%)	7 (470%)
	NRVV: Vaxzevria (Oxford–AstraZeneca)	..	1 (11%)	1 (14%)	0 (0%)	0 (0%)	1 (7%)
	NRVV: Ad26.COV2.S (Janssen)	..	1 (11%)	1 (14%)	0 (0%)	0 (0%)	1 (7%)
	Mixed (inactivated plus NRVV plus mRNA)‡	..	2 (22%)	1 (14%)	1 (25%)	0 (0%)	3 (20%)
Type of reinfection§							
	Confirmed	3 (27%)	3 (33%)	0 (0%)	0 (0%)	0 (0%)	3 (20%)
	Probable	6 (55%)	6 (67%)	7 (100%)	4 (100%)	0 (0%)	11 (73%)
	Possible	2 (18%)	0 (0%)	0 (0%)	0 (0%)	1 (100%)	1 (7%)
Reinfection severity¶							
	Any reinfection	10 (91%)	8 (89%)	6 (86%)	4 (100%)	1 (100%)	13 (87%)
	Severe disease (includes hospital admission)	7 (64%)	6 (67%)	3 (43%)	1 (25%)	0 (0%)	7 (47%)
	Severe disease (as per WHO definition)	4 (36%)	4 (44%)	0 (0%)	0 (0%)	0 (0%)	4 (27%)
Adjustment of primary estimate							
	None	2 (18%)	1 (11%)	2 (29%)	1 (25%)	1 (100%)	2 (13%)
	Population characteristics only	3 (27%)	0 (0%)	1 (14%)	0 (0%)	0 (0%)	1 (7%)
	Time only	0 (0%)	0 (0%)	1 (14%)	1 (25%)	0 (0%)	1 (7%)
	Population and time	6 (55%)	8 (89%)	3 (43%)	2 (50%)	0 (0%)	11 (73%)

Data are n (%) or median (IQR). NRVV=non-replicating viral vector.

* Seven studies reported results on both the protection conferred by previous infection and hybrid immunity. Hybrid immunity studies can be included in more than one column where they report multiple effect measures.

† Percentages in the columns summarising hybrid immunity studies can add up to more than 100% where studies report multiple vaccine types.

‡ The mixed vaccination category included the following types of vaccines: inactivated (BBIBP-CorV [Sinopharm]); non-replicating viral vector (Vaxzevria [Oxford–AstraZeneca], Ad26.COV2.S [Janssen], and Sputnik-V [Gamaleja]); and mRNA (BNT162b2 [Pfizer–BioNTech] and mRNA-1273 [Moderna]).

§ SARS-CoV-2 reinfection was defined as a possible, probable, or confirmed reinfection case according to adapted WHO definitions.^11^

¶ Percentages can add up to more than 100% where studies report multiple levels of reinfection severity.

**Figure 2:**
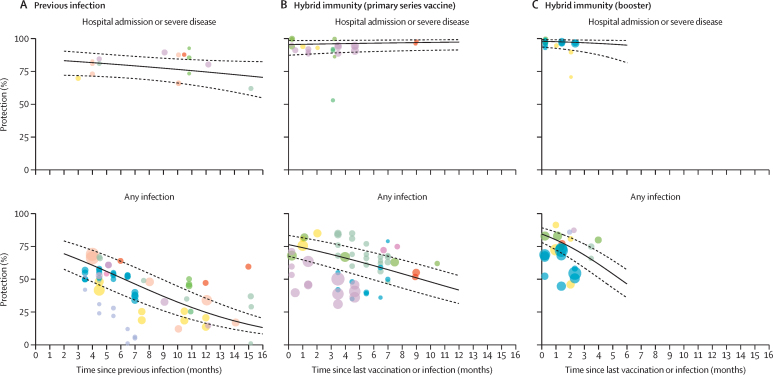
Protection against omicron variant conferred by previous infection or hybrid immunity compared to immune-naive individuals over time This analysis uses a log-odds meta-regression model. Points of the same color represent estimates from the same study. The diameter of the circles varies with the sample size of the study. Dotted lines represent 95% CIs. Solid black lines represent point estimates.

11 studies comprising a median of 294 900 (IQR 83 251–1 142 605) participants reported the protective effectiveness of previous infection (appendix pp 14–17). Of these 11 studies, six reported on protection against hospital admission or severe disease, ten reported on protection against reinfection, and six reported protection against both, with the longest follow-up at 15 months (table 2, figure 2). The effectiveness of previous infection against hospital admission or severe disease was 82·5% (95% CI 71·8 to 89·7) at 3 months; this was stable over time, reaching 74·6% (63·1 to 83·5) at 12 months (percentage point change –7·8% [95% CI –20·9 to 12·1]) and 71·6% (57·1 to 82·6) at 15 months (percentage point change –10·9% [–29·4 to 12·7]). The effectiveness of previous infection against reinfection was 65·2% (95% CI 52·9 to 75·9) at 3 months, dropping to 24·7% (16·4 to 35·5) at 12 months (percentage point change –40·5% [95% CI –51·9 to –33·9]) and 15·5% (9·9 to 23·6) at 15 months (percentage point change –49·7% [–42·2 to –62·8]).

**Table 2:** Protection conferred by previous infection and hybrid immunity compared to immune-naive individuals

	**Number of studies**	**Number of estimates**	Month 1*	**Month 2**†	**Month 3**	**Month 4**	**Month 6**	**Month 9**	**Month 12**	**Month 15**	**Percentage point change in protection, 3–6 months (95% CI)**‡	**Percentage point change in protection, 3–12 months (95% CI)**‡
**Previous infection**												
Hospital admission or severe disease	6	16	NA	83·2% (72·1 to 90·5)	82·5% (71·8 to 89·7)	81·7% (71·4 to 88·9)	80·1% (70·3 to 87·2)	77·5% (67·5 to 85·1)	74·6% (63·1 to 83·5)	71·6% (57·1 to 82·6)	−2·4 (−5·1 to 4·7)	−7·8 (−20·9 to 12·1)
Any infection§	10	64	NA	69·5% (57·6 to 79·2)	65·2% (52·9 to 75·9)	60·7% (48 to 72·1)	51·2% (38·6 to 63·7)	37·0% (26 to 49·6)	24·7% (16·4 to 35·5)	15·5% (9·9 to 23·6)	−14·0 (−12·0 to −18·2)	−40·5 (−33·9 to −51·9)
**Hybrid immunity (primary series vaccination)**												
Hospital admission or severe disease	5	23	95·7% (88·0 to 98·5)	95·9% (88·5 to 98·6)	96·0% (89·0 to 98·6)	96·2% (89·4 to 98·7)	96·5% (90·2 to 98·8)	97·0% (90·9 to 99)	97·4% (91·4 to 99·2)¶	NA	0·50 (−2·2 to 2·1)	1·3 (−4·3 to 7·4)
Any infection	7	55	74·1% (64·8 to 81·6)	71·6% (61·9 to 79·6)	69·0% (58·9 to 77·5)	66·2% (55·8 to 75·3)	60·4% (49·6 to 70·3)	51·1% (40·2 to 61·9)	41·8% (31·5 to 52·8)¶	NA	−8·6 (−1·7 to −17·2)	−27·2 (−6·4 to −53·2)
**Hybrid immunity (first booster vaccination)**												
Hospital admission or severe disease	4	17	98·0% (92·9 to 99·5)	97·6% (91·6 to 99·4)	97·2% (90·0 to 99·3)	96·7% (87·9 to 99·1)	95·3% (81·9 to 98·9)¶	NA	NA	NA	−1·8 (−10·3 to 0·77)	NA
Any infection	6	24	80·1% (72·5 to 86)	74·8 (66·0 to 81·9)	68·6% (58·8 to 76·9)	61·6% (51·2 to 71·1)	46·5% (36·0 to 57·3)¶	NA	NA	NA	−22·0 (−4·3 to −38·8)	NA

This table displays the point estimates and 95% CIs of protection shown in figure 2. This analysis used a log-odds meta-regression model. NA=not available (ie, insufficient data for model extrapolation).

* Month 1 data were for individuals with hybrid immunity whose last immunological challenge was vaccination and thus were eligible for reinfection within a shorter timeframe than people who most recently had previous infection (2 months minimum for probable reinfection).

† Month 2 data represent the minimum time period for reinfection among individuals with previous infection (ie, possible reinfection).

‡ 95% CIs calculated with the bootstrap method. Percentage point changes over time are reported from 3 months as this represents probable and confirmed reinfections.

§ Any infections comprised mild infections, symptomatic infections, and asymptomatic infections.

¶ Model predictions beyond the range of the available data. Previous infection data are available for predictions for 2–16 months; hybrid immunity data were available for predictions for 1–11 months. Data were extrapolated to a maximum of 3 months beyond the final follow-up date.

Nine studies, involving a median of 317 110 (IQR 50 576–696 439) participants, reported the protective effectiveness of hybrid immunity compared to immune-naive individuals, all of which reported on infection in combination with primary series vaccination. Seven studies, involving a median of 317 110 (IQR 172 738–807 329) participants, reported on the protective effectiveness of previous infection in combination with the first booster vaccination.

Of the nine studies that evaluated the protection conferred by hybrid immunity with primary series vaccination over time, five reported on protection against hospital admission or severe disease, seven reported on protection against reinfection, and three reported protection against both, with the longest follow-up at 11 months (table 2, figure 2). The effectiveness of hybrid immunity (with primary series vaccination) against hospital admission or severe disease was 96·0% (95% CI 89·0 to 98·6) at 3 months and remained stable at 97·4% (91·4 to 99·2) in projections at 12 months (percentage point change 1·3% [95% CI –4·3 to 7·4]). The effectiveness of hybrid immunity (with primary series vaccination) against reinfection was 69·0% (95% CI 58·9 to 77·5) at 3 months, dropping to 41·8% (31·5 to 52·8) at 12 months (percentage point change –27·2% [95% CI –6·4 to –53·2]).

Of the seven studies that evaluated the protection conferred by hybrid immunity with the first booster vaccination over time, four reported on protection against hospital admission or severe disease, six reported on protection against reinfection, and three reported on protection against both, with the longest follow-up of 4 months (table 2, figure 2). The effectiveness of hybrid immunity with the first booster vaccination against hospital admission or severe disease was 97·2% (95% CI 90·0 to 99·3) at 3 months and remained stable at 95·3% (81·9 to 98·9) in projections at 6 months (percentage point change –1·8 [95% CI –10·3 to 0·77]). The effectiveness of hybrid immunity with the first booster against reinfection was 68·6% (95% CI 58·8 to 76·9) at 3 months, dropping to 46·5% (36·0 to 57·3) at 6 months (percentage point change –22·0 [95% C I–4·3 to –38·8]).

Seven studies, involving a median of 130 073 (IQR 14 625–470 984) participants, reported the comparative protective effectiveness of hybrid immunity relative to individuals with previous infection only, of which five reported on hybrid immunity with primary series vaccination, and five reported on hybrid immunity with the first booster vaccination (appendix pp 18–37). Of these seven studies, one reported protection against hospital admission or severe disease, four reported protection against reinfection, and two reported protection against both, with the longest follow-up of 11 months (appendix pp 18–37). Data were insufficient for meta-regression; however, the meta-analysis showed that hybrid immunity conferred a significant gain in protection compared to previous infection alone, including with partial primary series (28·9% [95% CI 14·4–49·6] against hospital admission or severe disease [two studies] and 59·0% [51·5–66·1] against reinfection [two studies]); full primary series (57·7% [28·6–82·2] against hospital admission or severe disease [three studies] and 46·1% [30·6–62·4] against reinfection [four studies]), and the first booster vaccination (80·1% [48·6–94·5] against hospital admission or severe disease [three studies] and 46·5% [24·6–69·9%] against reinfection [four studies]; table 3).

**Table 3:** Comparative protective effectiveness of hybrid immunity relative to previous infection, vaccination, and hybrid immunity with fewer vaccine doses

	**Exposure**	**Comparator**	**Number of studies**	**Number of estimates**	**Pooled relative protection (95% CI)**
**Hybrid immunity *vs* previous infection**					
Hospital admission or severe disease	Previous infection plus partial primary series vaccine	Previous infection	2	3	28·9% (14·4–49·6)
Any infection	Previous infection plus partial primary series vaccine	Previous infection	2	2	59·0% (51·5–66·1)
Hospital admission or severe disease	Previous infection plus full primary series	Previous infection	3	4	57·7% (28·6–82·2)
Any infection	Previous infection plus full primary series	Previous infection	4	5	46·1% (30·6–62·4)
Hospital admission or severe disease	Previous infection plus first booster dose	Previous infection	3	3	80·1% (48·6–94·5)
Any infection	Previous infection plus first booster dose	Previous infection	4	5	46·5% (24·6–69·9)
**Hybrid immunity *vs* vaccination**					
Any infection	Previous infection plus first booster dose	First booster dose	1	1	88·9% (29·5–98·2)
**Hybrid immunity *vs* hybrid immunity with fewer vaccine doses**					
Hospital admission or severe disease	Previous infection plus full primary series	Previous infection plus partial primary series vaccine	1	2	49·6% (19·9–79·7)
Any infection	Previous infection plus full primary series	Previous infection plus partial primary series vaccine	1	1	16·3% (11·1–21·2)
Hospital admission or severe disease	Previous infection plus first booster dose	Previous infection plus partial primary series vaccine	1	1	67·1% (21·1–86·3)
Any infection	Previous infection plus first booster dose	Previous infection plus partial primary series vaccine	2	2	5·8% (0·2–65·2)
Hospital admission or severe disease	Previous infection plus first booster dose	Previous infection plus full primary series	1	2	37·0% (6·2–83·9)
Any infection	Previous infection plus first booster dose	Previous infection plus full primary series	3	3	40·8% (22·0–62·7)

There were insufficient data available for a sensitivity analysis.

Four studies, involving a median of 75 643 (IQR 17 919–271 664) participants, reported the comparative protective effectiveness of hybrid immunity with more vaccine doses relative to individuals with hybrid immunity with fewer vaccine doses. Of these four studies, three reported protection against reinfection and one reported protection against both reinfection and hospital admission or severe disease, with the longest follow-up of 3 months (appendix pp 18–37). In general, hybrid immunity with a greater number of vaccinations conferred significant gains in protection against both hospital admission or severe disease and reinfection; however, data were scarce for some comparisons (table 3).

One cohort study of 38 older adults (aged 71–101 years) done in France^19^ reported the comparative protective effectiveness of hybrid immunity with the first booster vaccination series relative to the first booster vaccination only with the BNT162b2 (Pfizer–BioNTech) or mRNA-1273 (Moderna) vaccines. After 90 days since the last immunological challenge, the estimate of protection against reinfection with the omicron variant was 88·9% (95% CI 29·5–98·2), indicating that hybrid immunity in combination with the first booster vaccination conferred a significant gain in protection compared to first booster vaccination alone (appendix p 25).

The appendix includes the subgroup analyses and summaries of results for studies reporting data by age (pp 43–44), vaccine type (p 45), and index variant (p 46).

Sensitivity analyses excluding estimates with a serious risk of bias and excluding hospital admission from the severe disease outcome showed similar findings as the main analyses (appendix pp 47–49). Definitions of severe disease used in each study are reported in the appendix (p 51).

**Figure 3:**
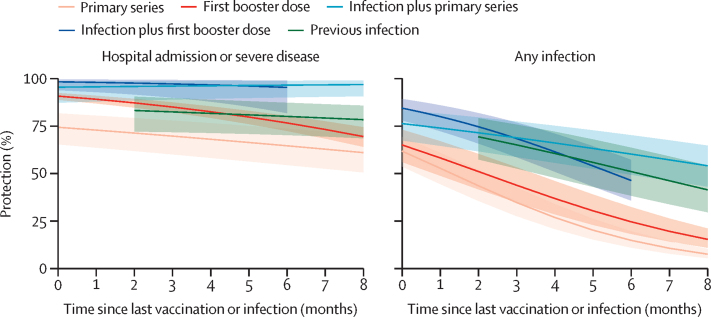
Protection against omicron variant conferred by the primary series vaccine, first booster vaccine, previous infection, and hybrid immunity compared to immune-naive individuals over time The shaded areas denote 95% CIs. Vaccine effectiveness data were procured from a separate systematic review.

Meta-regression showed that against hospital admission or severe disease at 6 months, hybrid immunity with the first booster vaccination (95·3% [95% CI 81·9–98·9]) or hybrid immunity with the primary series (96·5% [90·2–98·8]) provided significantly greater protection than previous infection alone (80·1% [70·3–87·2]), the first booster vaccination alone (76·7% [72·5–80·4]), or the primary series alone (64·6% [54·5–73·6]; figure 3; appendix p 52). Against reinfection at 6 months, there was a similar protection conferred by hybrid immunity with the first booster vaccination (46·5% [95% CI 36·0–57·3]), hybrid immunity with the primary series (60·4% [49·6–70·3]), and previous infection alone (51·2% [38·6–63·7]), with all three types of immunity conferring significantly greater protection than primary series vaccination alone (15·1% [11·3–19·8]) or first booster vaccination alone (24·8% [18·5–32·5]; figure 3; appendix p 52).

## Discussion

This systematic review and meta-regression found that both previous infection alone and previous infection combined with previous vaccination (ie, hybrid immunity) conferred rapidly waning protection against SARS-CoV-2 infection with the omicron variant, but high and sustained protection against hospital admission or severe disease due to the omicron variant. Previous infection was found to provide higher protection against reinfection and more sustained protection against hospital admission or severe disease than vaccination alone. However, individuals with hybrid immunity had the highest magnitude and durability of protection against all outcomes, emphasising the importance of providing vaccination to previously infected individuals.

Another systematic review similarly reported rapid waning of protection against infection with the omicron variant, but was unable to infer protection against severe disease over time and did not examine hybrid immunity.^7^ Previous studies have similarly reported that previous infection confers more durable protection than vaccination.^31^ This pattern might be explained by natural infection invoking a more diverse immune response to multiple antigenic sites on the virus compared to the immunity developed through vaccines that target only spike antigens.

Protection from previous infection should not detract from the need for vaccination. Infection-induced protection against reinfection wanes rapidly, and vaccination increases durability. Furthermore, there are serious risks associated with infection. These include the risks of hospital admission, ICU admission and mechanical ventilation, and death; they also include the risk of developing post-COVID-19 complications. Additionally, those who recover from severe COVID-19 have a higher risk of cardiovascular complications, neurological complications, dementia, diabetes, and chronic respiratory problems.^32^, ^33^ Vaccination is therefore a safe intervention to avert severe disease outcomes and to reduce post-COVID-19 complications.^34^ Reassuringly, vaccination after natural infection is not thought to be associated with an increased risk of reactogenicity or other safety concerns.^35^

At the individual level, our results show that the need for, and optimal timing of, the primary vaccination series and booster dose might be different in an individual who has previously had SARS-CoV-2 infection or who has had a breakthrough infection after initiation of the primary series compared to a previously uninfected individual. Our findings are in line with a recent study that reported a higher quality and magnitude of immune responses (antibodies and B cells) with a longer interval between infection and booster vaccination (>180 days).^36^ It might therefore be reasonable for individuals with a previous infection and full primary series vaccination to delay subsequent doses of vaccination by 6 months, while still maintaining high levels of protection against severe disease.

On a population level, the optimal number of vaccine doses and the inter-dose interval might differ in settings with various degrees of vaccine-induced versus infection-induced immunity. However, well designed serosurveys are required for estimating infection-induced seroprevalence rates and reliable estimates can only be generated in countries where inactivated vaccines were not used. Considering this caveat, basing national vaccination policies on infection-induced seroprevalence rates could be challenging in many settings.

In countries where only S-protein antigen vaccines were used, it might be possible to classify individuals as previously infected with an anti-N antibody test and use this to modify recommendations for boosters. However, this approach might not be reasonable for high-risk groups, including older people and immunocompromised individuals, since our data were not stratified by patient demographics. Caution is also required as programmatic vaccine rollout should be as simple as possible; modifying the number of vaccines and intervals by infection-induced seroprevalence rates could complicate vaccine programmes and thus hamper vaccine uptake. Furthermore, pre-vaccination screening for past infections is not recommended by WHO,^37^ similar to other mass vaccination programmes (eg, measles) where past infections are not a reason to exclude individuals from vaccination or delay vaccination. Additionally, there might still be benefit in providing boosters before periods with expected increased incidence, such as the winter season, to individuals whose last immunological challenge is unknown. In fact, this scenario is common as the prevalence of infection has been largely underestimated in most settings throughout the pandemic.^3^

Our systematic review had several potential limitations. First, the patterns of declining protection observed in this study might be explained by waning immunity; however, these results could also be in part attributable to unmeasured biases. The observational studies we included assumed that all individuals had the same risk of exposure. However, differential depletion of susceptibles bias could have occurred, where, when the vaccine is effective, people who are infected are more likely to be unvaccinated than vaccinated, thus reducing the proportion of susceptible individuals in the unvaccinated group and creating the appearance of waning.^38^ Exposure could also differ between groups, as in the case of individuals who are unvaccinated because they are severely immunocompromised, and thus also have a greater risk of infection. The likelihood of measuring an outcome could also differ between groups (eg, individuals with no previously reported infection who might not have had access to testing). Individual studies adjusted for some of these factors (eg, calendar time, age, comorbidities, and testing frequency) and these adjustments were considered in the risk of bias assessment; however, not all studies reported these adjustments. Second, our analysis did not incorporate the sequence of and timing between vaccination and previous infection for hybrid immunity. Nine studies reported information on the sequence of immunological challenges; however, the data were spread across different types of exposures (eg, hybrid immunity with different numbers of vaccine doses) and sequence permutations. Data from studies measuring neutralising antibodies suggest that the sequence and timing of immunological challenges could interact with the level of protection conferred,^36^, ^39^ but further studies with linked individual-level data are needed. Third, data were scarce for some analyses, which implies the need for caution in interpretation of results, particularly for estimates of the comparative protective effectiveness of hybrid immunity relative to vaccination, estimates by vaccination type, and estimates by the variant causing the primary infection. Scarce data also precluded stratified analyses by age, sex, ethnicity, severity of primary infection, and protection against the distinct omicron subvariants. Stratifications by participant demographic characteristics were often omitted by researchers and should be consistently reported. Fourth, in the 11 studies reporting data based on multiple vaccines, the relative proportions of individuals vaccinated by each product was unclear, complicating our ability to generate estimates specific to vaccine brands. Fifth, the vaccine effectiveness data were procured from a separate systematic review. However, the inclusion and exclusion criteria were similar to our study. Furthermore, we re-analysed the raw data using the same method that was used for the data on previous infection and hybrid immunity. Sixth, we were only able to examine protection conferred by pre-omicron SARS-CoV-2 variants (ie, the index virus through to the delta [B.1.617.2] variant). Future evidence synthesis will be needed to ascertain the protection conferred by the omicron variant against reinfection.

Our findings make clear the substantial durability of hybrid immunity and could help inform the timing and prioritisation of vaccination programmes in populations with high rates of past infection. Further follow-up is needed to assess the protective effectiveness of hybrid immunity against hospital admission or severe disease, the two outcomes that drive most COVID-19 policy decisions, to clarify how much waning of protection might occur over a longer duration, especially if new variants of concern emerge. More precise quantification of the duration of this protection will help inform the necessity and timing of future booster vaccinations. Policy makers can use these findings to project population protection from local vaccination and seroprevalence rates, helping to inform the use and timing of COVID-19 vaccination as an important public health tool.

For more on the **GISAID EpiFlu database s**ee https://www.re3data.org/repository/r3d100010126

For the **statistical code** see https://github.com/serotracker/protective-effectiveness

## Data sharing

The statistical code for our main model is available online on GitHub. Data extracted from published articles and used in our analysis will be made available upon request via email to the corresponding author. Data provided to us directly by authors of the included studies will not be shared unless the investigators requesting data obtain permission from the original study authors.

## Declaration of interests

NB, HW, XM, ZL, RH, CC, AS, MW, BC, and RKA report grants unrelated to the present work from the WHO Health Emergencies Program, the Public Health Agency of Canada through Canada's COVID-19 Immunity Task Force (grant 2021-HQ-000056), the Robert Koch Institute, and the Canadian Medical Association Joule Innovation Fund. HI reports contracts unrelated to the present work from WHO. MMH reports contracts unrelated to the present work from WHO, Pfizer, and the Bill & Melinda Gates Foundation. VP is an unpaid governing board member and Royal Trustee at Cochrane. RKA reports consulting fees unrelated to the present work from the Bill & Melinda Gates Foundation Strategic Investment Fund, Health Canada, and Flagship Pioneering, and stock in Alethea Medical that is unrelated to the present work. MDVK, IB, DF, and LS are employed by WHO. MMH has contracts with WHO, CEPI, and the Asian Development Bank to gather data on COVID-19 vaccine efficacy, effectiveness, and safety from published studies, and reports unrelated grants from Pfizer and the Bill & Melinda Gates Foundation. AW-S and MKP consult on COVID-19 research and policy for WHO.
