# Supplementary material for: Protective effectiveness of previous SARS-CoV-2 infection and hybrid immunity against the omicron variant and severe disease: a systematic review and meta-regression
Source: Lancet Infect Dis. 2023 May;23(5):556–67. doi: 10.1016/S1473-3099(22)00801-5 (PMC10014083; doi:10.1016/S1473-3099(22)00801-5)
Supplement: Supplementary appendix [file mmc1.pdf]

THE LANCET
Infectious Diseases

Supplementary appendix

This appendix formed part of the original submission and has been peer reviewed. We post it as supplied by the authors.

Supplement to: Bobrovitz N, Ware H, Ma X, et al. Protective effectiveness of previous SARS-CoV-2 infection and hybrid immunity against the omicron variant and severe disease: a systematic review and meta-regression. *Lancet Infect Dis* 2023; published online Jan 18. https://doi.org/10.1016/S1473-3099(22)00801-5.

Appendices

Appendix 1. PRISMA checklist 2

Appendix 2. Search strategy 5

Appendix 3. Additional methodological details 10

Appendix 4. Detailed list of comparisons with corresponding effect measures and epidemiological and policy questions 13

Appendix 5. Characteristics and results of individual prior infection studies included in meta-analysis 14

Appendix 6. Characteristics and results of individual hybrid immunity studies included in meta-analysis 18

Appendix 7. Risk of bias assessments 38

Appendix 8. Summary of results for studies reporting sub-group data by age 43

Appendix 9. Sub-group analysis of the protective effectiveness of hybrid immunity by vaccine type 45

Appendix 10. Sub-group analysis of the protective effectiveness of prior infection and hybrid immunity by prior infection variant 46

Appendix 11. Sensitivity analysis of the protection conferred by prior infection and hybrid immunity compared to immune naïve for studies at different risk of bias 47

Appendix 12. Sensitivity analysis of protection conferred by prior infection or hybrid immunity over time using the WHO definition of severe disease 49

Appendix 13. Sensitivity analysis of the protection against reinfection and severe disease conferred by the primary-series vaccine, first booster vaccine, prior infection, and hybrid immunity compared to immune naive 50

Appendix 14. Definitions of severe disease in included studies 51

Appendix 15. Six-month protection against reinfection and severe disease conferred by the primary-series vaccine, first booster vaccine, prior infection, and hybrid immunity compared to immune naive individuals 52

Appendix 16. Characteristics of vaccine effectiveness studies 53

Appendix 17. References for the supplement 54

Appendix 1. PRISMA checklist

Section and Topic	Item #	Checklist item	Location where item is reported
TITLE			
Title	1	Identify the report as a systematic review.	Page 1
ABSTRACT			
Abstract	2	See the PRISMA 2020 for Abstracts checklist.	Page 2
INTRODUCTION			
Rationale	3	Describe the rationale for the review in the context of existing knowledge.	Page 5, 6
Objectives	4	Provide an explicit statement of the objective(s) or question(s) the review addresses.	Page 2-4
METHODS			
Eligibility criteria	5	Specify the inclusion and exclusion criteria for the review and how studies were grouped for the syntheses.	Page 6, Appendix 3
Information sources	6	Specify all databases, registers, websites, organisations, reference lists and other sources searched or consulted to identify studies. Specify the date when each source was last searched or consulted.	Page 5,6
Search strategy	7	Present the full search strategies for all databases, registers and websites, including any filters and limits used.	Page 5,6, Appendix 2
Selection process	8	Specify the methods used to decide whether a study met the inclusion criteria of the review, including how many reviewers screened each record and each report retrieved, whether they worked independently, and if applicable, details of automation tools used in the process.	Page 7
Data collection process	9	Specify the methods used to collect data from reports, including how many reviewers collected data from each report, whether they worked independently, any processes for obtaining or confirming data from study investigators, and if applicable, details of automation tools used in the process.	Page 6, 7
Data items	10a	List and define all outcomes for which data were sought. Specify whether all results that were compatible with each outcome domain in each study were sought (e.g. for all measures, time points, analyses), and if not, the methods used to decide which results to collect.	Page 6,7,. Appendix 3
	10b	List and define all other variables for which data were sought (e.g. participant and intervention characteristics, funding sources). Describe any assumptions made about any missing or unclear information.	Page 6,7, Appendix 3
Study risk of bias assessment	11	Specify the methods used to assess risk of bias in the included studies, including details of the tool(s) used, how many reviewers assessed each study and whether they worked independently, and if applicable, details of automation tools used in the process.	Page 7

Effect measures	12	Specify for each outcome the effect measure(s) (e.g. risk ratio, mean difference) used in the synthesis or presentation of results.	Page 6-8 Appendix 3, 4
Synthesis methods	13a	Describe the processes used to decide which studies were eligible for each synthesis (e.g. tabulating the study intervention characteristics and comparing against the planned groups for each synthesis (item #5)).	Page 6-8 Appendix 3
	13b	Describe any methods required to prepare the data for presentation or synthesis, such as handling of missing summary statistics, or data conversions.	Page 6-8 Appendix 3
	13c	Describe any methods used to tabulate or visually display results of individual studies and syntheses.	Page 6-8 Appendix 3
	13d	Describe any methods used to synthesize results and provide a rationale for the choice(s). If meta-analysis was performed, describe the model(s), method(s) to identify the presence and extent of statistical heterogeneity, and software package(s) used.	Page 6- 8 Appendix 3, 4
	13e	Describe any methods used to explore possible causes of heterogeneity among study results (e.g. subgroup analysis, meta-regression).	Page 8, Appendix 3
	13f	Describe any sensitivity analyses conducted to assess robustness of the synthesized results.	Page 8, Appendix 3
Reporting bias assessment	14	Describe any methods used to assess risk of bias due to missing results in a synthesis (arising from reporting biases).	N/A
Certainty assessment	15	Describe any methods used to assess certainty (or confidence) in the body of evidence for an outcome.	N/A
RESULTS			
Study selection	16a	Describe the results of the search and selection process, from the number of records identified in the search to the number of studies included in the review, ideally using a flow diagram.	Page 8, Figure 1
	16b	Cite studies that might appear to meet the inclusion criteria, but which were excluded, and explain why they were excluded.	Figure 1
Study characteristics	17	Cite each included study and present its characteristics.	Page 8,9, Table 1, Appendix 5, 6
Risk of bias in studies	18	Present assessments of risk of bias for each included study.	Appendix 7
Results of individual studies	19	For all outcomes, present, for each study: (a) summary statistics for each group (where appropriate) and (b) an effect estimate and its precision (e.g. confidence/credible interval), ideally using structured tables or plots.	Appendix 5, 6
Results of syntheses	20a	For each synthesis, briefly summarise the characteristics and risk of bias among contributing studies.	Page 8-11, Table 1
	20b	Present results of all statistical syntheses conducted. If meta-analysis was done, present for each the summary estimate and its precision (e.g. confidence/credible interval) and measures of statistical heterogeneity. If comparing groups, describe the direction of the effect.	Page 8-11, Table 2, Table 3, Figure 2, Figure 3, Appendix 8, 9, 12-14

	20c	Present results of all investigations of possible causes of heterogeneity among study results.	Page 8-11, Appendix 8, 9, 12-14
	20d	Present results of all sensitivity analyses conducted to assess the robustness of the synthesized results.	Page 8-11, Appendix 8, 9, 12-14
Reporting biases	21	Present assessments of risk of bias due to missing results (arising from reporting biases) for each synthesis assessed.	N/A
Certainty of evidence	22	Present assessments of certainty (or confidence) in the body of evidence for each outcome assessed.	N/A
DISCUSSION			
Discussion	23a	Provide a general interpretation of the results in the context of other evidence.	Page 12
	23b	Discuss any limitations of the evidence included in the review.	Page 13, 14
	23c	Discuss any limitations of the review processes used.	Page 13, 14
	23d	Discuss implications of the results for practice, policy, and future research.	Page 12-14
OTHER INFORMATION			
Registration and protocol	24a	Provide registration information for the review, including register name and registration number, or state that the review was not registered.	Page 5
	24b	Indicate where the review protocol can be accessed, or state that a protocol was not prepared.	Page 5
	24c	Describe and explain any amendments to information provided at registration or in the protocol.	Appendix 3
Support	25	Describe sources of financial or non-financial support for the review, and the role of the funders or sponsors in the review.	Page 2, 8
Competing interests	26	Declare any competing interests of review authors.	Page 15
Availability of data, code and other materials	27	Report which of the following are publicly available and where they can be found: template data collection forms; data extracted from included studies; data used for all analyses; analytic code; any other materials used in the review.	Page 15

Appendix 2. Search strategy

Additional details of search strategies

The search strategy comprised three search keyword concepts: SARS-CoV-2, reinfection/protective effectiveness, and previous infection/presence of antibodies/hybrid immunity.

Comprehensive search strategies were designed by a medical librarian, for each database, incorporating both subject headings and keywords based on the individual database's unique thesaurus and available operators. The SARS-COV-2 search concept was adapted from the CADTH Covid-19 search string.[1] The search strategy was validated against known included articles from previously published reviews.

Results from the searches were exported on June 1st, 2022, as RIS and imported into Covidence software (www.covidence.org) for deduplication. Articles reporting identical information to previously included articles were excluded as duplicates. This rule extended to pre-print articles that were subsequently published in peer-reviewed journals. In these cases, the peer-reviewed articles were considered the definitive version.

We searched databases from 1 January 2020 to 1 June 2022. Pre-print articles that were identified during this period and subsequently updated or published as peer-review articles between 1 June 2022 and 15 July 2022 were also included.

Search strategies used for electronic databases

MEDLINE(R) ALL <Ovid; 2020 to June 01, 2022>

#	Query	Results
1	COVID-19/ or exp COVID-19 Testing/ or COVID-19 Vaccines/ or SARS-CoV-2/	166,472
2	(coronavirus/ or betacoronavirus/ or coronavirus infections/) and (disease outbreaks/ or epidemics/ or pandemics/)	40,140
3	(nCoV* or 2019nCoV or 19nCoV or COVID19* or COVID or SARS-COV-2 or SARSCOV-2 or SARS-COV2 or SARSCOV2 or SARS coronavirus 2 or Severe Acute Respiratory Syndrome Coronavirus 2 or Severe Acute Respiratory Syndrome Corona Virus 2).ti,ab,kf,nm,ot,ox,rx,px.	252,595
4	((new or novel or "19" or "2019" or Wuhan or Hubei or China or Chinese) adj3 (coronavirus* or corona virus* or betacoronavirus* or CoV or HCoV)).ti,ab,kf,ot.	70,526
5	(longCOVID* or postCOVID* or postcoronavirus* or postSARS*).ti,ab,kf,ot.	40
6	((coronavirus* or corona virus* or betacoronavirus*) adj3 (pandemic* or epidemic* or outbreak* or crisis)).ti,ab,kf,ot.	12,534
7	((Wuhan or Hubei) adj5 pneumonia).ti,ab,kf,ot.	396
8	((Alpha or "B.1.1.7" or Beta or "B.1.351" or Delta or "B.1.617.2" or Omicron or "B.1.1.529" or gamma or lambda) adj3 variant*).tw,kf.	9,600
9	1 or 2 or 3 or 4 or 5 or 6 or 7 or 8	270,102
10	Reinfection/ or Recurrence/	195,522
11	(reinfect* or re-infect*).tw,kf,ot.	13,909
12	((repeat* or second* or reactivat* or recurrent*) adj2 infect*).tw,kf,ot.	32,668
13	((subsequent* or future) adj infect*).tw,kf,ot.	3,601
14	(("repeat positive" or "re-positive" or "two positive" or "2 positive") adj5 (pcr or polymerase chain reaction)).tw,kf,ot.	231
15	(new infection* or new SARS-CoV-2 infection*).tw,kf,ot.	4,404
16	((risk adj3 infect*) or (protect* adj3 (infect* or postinfect*))).tw,kf,ot.	83,466
17	10 or 11 or 12 or 13 or 14 or 15 or 16	321,390
18	9 and 17	8,847

19	((natural* or primary) adj2 (infect* or immunit*)).tw,kf,ot.	41,996
20	(low vaccin* or unvaccin* or un-vaccin* or "not vaccin*").tw,kf,ot.	11,555
21	("anti-SARS-CoV-2 IgG" or seropositiv* or "SARS-CoV-2 antigen positiv*" or "anti-nucleocapsid IgG antibod*" or "antibody positiv*").tw,kf,ot.	52,616
22	(((prior or previous* or earlier or initial or past) adj2 (infect* or disease or "covid-19" or covid19 or "SARS-CoV-2" or coronavirus or "corona virus")) or ((first or history) adj3 infection*)).tw,kf,ot.	61,975
23	(("covid-19" or covid19) adj2 (recovered or recovery)).tw,kf,ot.	1,998
24	((recovered or convalescen*) adj1 (patient* or population* or person or persons or cases or adults or covid* or sars* or coronavirus*)).tw,kf,ot.	18,021
25	((hybrid adj3 immunity) or (immunity adj3 (infect* or postinfect*))).tw,kf,ot.	9,108
26	19 or 20 or 21 or 22 or 23 or 24 or 25	188,381
27	18 and 26	1,403
28	limit 27 to yr="2020 - 2022"	1,398

Embase <Ovid; 2020 to 2022 June 01>

#	**Query**	**Results**
1	sars-related coronavirus/ or coronavirus disease 2019/ or asymptomatic coronavirus disease 2019/ or long covid/ or exp Severe acute respiratory syndrome coronavirus 2/ or exp SARS coronavirus/ or exp "variant of concern"/	239,763
2	(coronavirinae/ or betacoronavirus/ or coronavirus infection/) and (epidemic/ or pandemic/)	10,874
3	(nCoV* or 2019nCoV or 19nCoV or COVID19* or COVID or SARS-COV-2 or SARSCOV-2 or SARS-COV2 or SARSCOV2 or SARS coronavirus 2 or Severe Acute Respiratory Syndrome Coronavirus 2 or Severe Acute Respiratory Syndrome Corona Virus 2).ti,ab,kf,hw,ot.	273,124
4	((new or novel or "19" or "2019" or Wuhan or Hubei or China or Chinese) adj3 (coronavirus* or corona virus* or betacoronavirus* or CoV or HCoV)).ti,ab,kf,hw,ot.	235,343
5	(longCOVID* or postCOVID* or postcoronavirus* or postSARS*).ti,ab,kf,hw,ot.	102
6	((coronavirus* or corona virus* or betacoronavirus*) adj3 (pandemic* or epidemic* or outbreak* or crisis)).ti,ab,kf,ot.	12,307
7	((Wuhan or Hubei) adj5 pneumonia).ti,ab,kf,ot.	462
8	((Alpha or "B.1.1.7" or Beta or "B.1.351" or Delta or "B.1.617.2" or Omicron or "B.1.1.529" or gamma or lambda) adj3 variant*).tw,kf,ot.	10,083
9	or/1-8	303,594
10	reinfection/ or recurrent infection/	31,916
11	(reinfect* or re-infect*).tw,kf,ot.	17,000
12	((repeat* or second* or reactivat* or recurrent*) adj2 infect*).tw,kf,ot.	47,197
13	((subsequent* or future) adj infect*).tw,kf,ot.	4,331
14	(("repeat positive" or "re-positive" or "two positive" or "2 positive") adj5 (pcr or polymerase chain reaction)).tw,kf,ot.	356
15	(new infection* or new SARS-CoV-2 infection*).tw,kf,ot.	5,757
16	((risk adj3 infect*) or (protect* adj3 (infect* or postinfect*))).tw,kf,ot.	111,501
17	or/10-16	193,620
18	9 and 17	10,163
19	primary infection/	4,596
20	((natural* or primary) adj2 (infect* or immunit*)).tw,kf,ot.	47,657
21	(low vaccin* or unvaccin* or un-vaccin* or "not vaccin*").tw,kf,ot.	13,590
22	("anti-SARS-CoV-2 IgG" or seropositiv* or "SARS-CoV-2 antigen positiv*" or "anti-nucleocapsid IgG antibod*" or "antibody positiv*").tw,kf,ot.	68,366

23	(((prior or previous* or earlier or initial or past) adj2 (infect* or disease or "covid-19" or covid19 or "SARS-CoV-2" or coronavirus or "corona virus")) or ((first or history) adj3 infection*)).tw,kf,ot.	93,696
24	(("covid-19" or covid19) adj2 (recovered or recovery)).tw,kf,ot.	2,295
25	((recovered or convalescen*) adj1 (patient* or population* or person or persons or cases or adults or covid* or sars* or coronavirus*)).tw,kf,ot.	24,455
26	((hybrid adj3 immunity) or (immunity adj3 (infect* or postinfect*))).tw,kf,ot.	10,849
27	or/19-26	250,722
28	18 and 27	1,659
29	limit 28 to yr="2020 - 2022"	1,642

Cochrane Central Register of Controlled Trials <Ovid; 2020 - June 1, 2022>

#	**Query**	**Results**
1	COVID-19/ or exp COVID-19 Testing/ or COVID-19 Vaccines/ or SARS-CoV-2/	1,693
2	(coronavirus/ or betacoronavirus/ or coronavirus infections/) and (disease outbreaks/ or epidemics/ or pandemics/)	132
3	(nCoV* or 2019nCoV or 19nCoV or COVID19* or COVID or SARS-COV-2 or SARSCOV-2 or SARS-COV2 or SARSCOV2 or SARS coronavirus 2 or Severe Acute Respiratory Syndrome Coronavirus 2 or Severe Acute Respiratory Syndrome Corona Virus 2).tw,kw.	10,613
4	((new or novel or "19" or "2019" or Wuhan or Hubei or China or Chinese) adj3 (coronavirus* or corona virus* or betacoronavirus* or CoV or HCoV)).tw,kw.	4,898
5	(longCOVID* or postCOVID* or postcoronavirus* or postSARS*).tw,kw.	6
6	((coronavirus* or corona virus* or betacoronavirus*) adj3 (pandemic* or epidemic* or outbreak* or crisis)).tw,kw.	245
7	((Wuhan or Hubei) adj5 pneumonia).tw,kw.	23
8	((Alpha or "B.1.1.7" or Beta or "B.1.351" or Delta or "B.1.617.2" or Omicron or "B.1.1.529" or gamma or lambda) adj3 variant*).tw,kw.	116
9	1 or 2 or 3 or 4 or 5 or 6 or 7 or 8	10,921
10	Reinfection/ or Recurrence/	12,532
11	(reinfect* or re-infect*).tw,kw.	1,308
12	((repeat* or second* or reactivat* or recurrent*) adj2 infect*).tw,kw.	2,580
13	((subsequent* or future) adj infect*).tw,kw.	162
14	(("repeat positive" or "re-positive" or "two positive" or "2 positive") adj5 (pcr or polymerase chain reaction)).tw,kw.	39
15	(new infection* or new SARS-CoV-2 infection*).tw,kw.	438
16	((risk adj3 infect*) or (protect* adj3 (infect* or postinfect*))).tw,kw.	7,338
17	10 or 11 or 12 or 13 or 14 or 15 or 16	23,434
18	9 and 17	420
19	((natural* or primary) adj2 (infect* or immunit*)).tw,kw.	1,581
20	(low vaccin* or unvaccin* or un-vaccin* or "not vaccin*").tw,kw.	778
21	("anti-SARS-CoV-2 IgG" or seropositiv* or "SARS-CoV-2 antigen positiv*" or "anti-nucleocapsid IgG antibod*" or "antibody positiv*").tw,kw.	3,502
22	(((prior or previous* or earlier or initial or past) adj2 (infect* or disease or "covid-19" or covid19 or "SARS-CoV-2" or coronavirus or "corona virus")) or ((first or history) adj3 infection*)).tw,kw.	5,291
23	(("covid-19" or covid19) adj2 (recovered or recovery)).tw,kw.	154

24	((recovered or convalescen*) adj1 (patient* or population* or person or persons or cases or adults or covid* or sars* or coronavirus*)).tw,kw.	767
25	((hybrid adj3 immunity) or (immunity adj3 (infect* or postinfect*))).tw,kw.	297
26	19 or 20 or 21 or 22 or 23 or 24 or 25	11,924
27	18 and 26	56

Web of Science- (A&HCI , BKCI-SSH , BKCI-S , CCR-EXPANDED , ESCI , IC , CPCI-SSH , CPCI-S , SCI-EXPANDED , SSCI) <2020 - June 1, 2022>

#	Search string	Results
1	(TS=((nCoV* or 2019nCoV or 19nCoV or COVID19* or COVID or SARS-COV-2 or SARSCOV-2 or SARS-COV2 or SARSCOV2 or SARS coronavirus 2 or Severe Acute Respiratory Syndrome Coronavirus 2 or Severe Acute Respiratory Syndrome Corona Virus 2))) OR TS=((((new or novel or "19" or "2019" or Wuhan or Hubei or China or Chinese) NEAR/3 (coronavirus* or "corona virus*" or betacoronavirus* or CoV or HCoV)) OR (longCOVID* or postCOVID* or postcoronavirus* or postSARS*) OR ((coronavirus* or "corona virus*" or betacoronavirus*) NEAR/3 (pandemic* or epidemic* or outbreak* or crisis)) OR ((Wuhan or Hubei) NEAR/5 pneumonia) OR ((Alpha or "B.1.1.7" or Beta or "B.1.351" or Delta or "B.1.617.2" or Omicron or "B.1.1.529" or gamma or lambda) NEAR/3 variant*)))<br><br>[Exact search applied]	318,009
2	TS=(((reinfect* or re-infect*) OR ((repeat* or second* or reactivat* or recurrent*) NEAR/2 infect*) OR ("new infection*" or "new SARS-CoV-2 infection*") OR (("repeat positive" or "re-positive" or "two positive" or "2 positive") NEAR/5 (pcr or “polymerase chain reaction”)) OR ((subsequent* or future) NEAR/1 infect*) OR (risk NEAR/3 infect*) OR (protect* NEAR/3 (infect* or postinfect*))))<br><br>[Exact search applied]	165,361
3	TS=((((natural* or primary) NEAR/2 (infect* or immunit*)) OR ("low vaccin*" or unvaccin* or "un-vaccin*" or "not vaccin*") OR ((prior or previous* or earlier or initial or past) NEAR/2 (infect* or disease or "covid-19" or covid19 or "SARS-CoV-2" or coronavirus or "corona virus")) or ((first or history) NEAR/4 infection*) OR "anti-SARS-CoV-2 IgG" or seropositiv* or "SARS-CoV-2 antigen positiv*" or "anti-nucleocapsid IgG antibod*" or "antibody positiv*" or (("immunity") NEAR/5 (infection* or postinfect* or hybrid)) OR (("covid-19" or covid19) NEAR/2 (recovered or recovery)) OR ((recovered or convalescen*) NEAR/1 (patient* or population* or person or persons or cases or adults or covid* or sars* or coronavirus*))))<br><br>[Exact search applied]	226,129
4	(#1 AND #2 AND #3) and 2022 or 2021 or 2020 (Publication Years)	1729

WHO Covid-19 Database - June 1, 2022 (1252 results)

(ti:(("reinfection" OR "reinfections" OR "re-infection" OR "re-infections" OR "Repeat infections" OR "recurrent infections" OR "repeat positive"))) OR (tw:(("reinfection" OR "reinfections" OR "re-infection" OR "re-infections" OR "Repeat infections" OR "recurrent infections" OR "repeat positive" OR "new infection" OR "risk of infection" OR "new SARS-CoV-2 infection") AND ("natural infection" OR "primary infection" OR "natural immunity" OR unvaccin* OR "un-vaccinated" OR "not vaccinated" OR "Prior infection" OR "previous infection" OR "first infection" OR "past infection" OR seropositiv* OR "antigen positive" OR "antibody positive" OR "postinfection immunity" OR "infection acquired immunity" OR "naturally acquired immunity" OR "recovered patients" OR "convalescent patients" OR "hybrid immunity" OR "hybrid protection")))

EuropePMC – June 1, 2022 (1044 results)

(ABSTRACT:(COVID19* OR COVID OR “SARS-COV-2” OR “SARSCOV-2” OR SARSCOV2 OR “Corona Virus” OR coronavirus OR postcovid OR longcovid) OR TITLE:(COVID19* OR COVID OR “SARS-COV-2” OR “SARSCOV-2” OR SARSCOV2 OR “Corona Virus” OR coronavirus OR postcovid OR longcovid) OR KW:(COVID19* OR COVID OR “SARS-COV-2” OR “SARSCOV-2” OR SARSCOV2 OR “Corona Virus” OR coronavirus OR postcovid OR longcovid)) AND (ABSTRACT:(reinfect* OR "re-infect*" OR "second* infection*" OR "repeat* infection*" OR “reactivated infection*” OR ”recurrent infection*” OR "new infection*" OR "new SARS-CoV-2 infection*" OR “subsequent infection*” OR “future infection*” OR “risk of infection*” OR “risk of Covid* infection”) OR TITLE:(reinfect* OR "re-infect*" OR "second* infection*" OR "repeat* infection*" OR “reactivated infection*” OR ”recurrent infection*” OR "new infection*" OR "new SARS-CoV-2 infection*" OR “subsequent infection*” OR “future infection*” OR “risk of infection*” OR “risk of Covid* infection”) OR KW:(reinfect* OR "re-infect*" OR "second* infection*" OR "repeat* infection*" OR “reactivated infection*” OR ”recurrent infection*” OR "new infection*" OR "new SARS-CoV-2 infection*" OR “subsequent infection*” OR “future infection*” OR “risk of infection*” OR “risk of Covid* infection”)) AND (ABSTRACT:(“natural infection*” OR “primary infection*” OR “natural immunity” OR “naturally acquired immunity” OR unvaccin* OR "un-vaccin*" OR "not vaccin*" OR “Prior infection*” OR “previous infection*” OR “previously infected” OR "first infection*" OR “past infection*” OR seropositiv* OR "antigen positiv*" or "anti-nucleocapsid IgG antibod*" or "antibody positiv*" OR "postinfection immunity” OR “infection acquired immunity” OR “recovered patient*" OR “convalescen* patient*” OR “hybrid immunity” OR “hybrid protection”) OR TITLE:(“natural infection*” OR “primary infection*” OR “natural immunity” OR “naturally acquired immunity” OR unvaccin* OR "un-vaccin*" OR "not vaccin*" OR “Prior infection*” OR “previous infection*” OR “previously infected” OR "first infection*" OR “past infection*” OR seropositiv* OR "antigen positiv*" or "anti-nucleocapsid IgG antibod*" or "antibody positiv*" OR "postinfection immunity” OR “infection acquired immunity” OR “recovered patient*" OR “convalescen* patient*” OR “hybrid immunity” OR “hybrid protection”) OR KW:(“natural infection*” OR “primary infection*” OR “natural immunity” OR “naturally acquired immunity” OR unvaccin* OR "un-vaccin*" OR "not vaccin*" OR “Prior infection*” OR “previous infection*” OR “previously infected” OR "first infection*" OR “past infection*” OR seropositiv* OR "antigen positiv*" or "anti-nucleocapsid IgG antibod*" or "antibody positiv*" OR "postinfection immunity” OR “infection acquired immunity” OR “recovered patient*" OR “convalescen* patient*” OR “hybrid immunity” OR “hybrid protection”)) AND (SRC:"PPR")

ClinicalTrials.Gov - June 1, 2022 (12 studies)

reinfection OR reinfections OR "re-infection" OR "re-infections" OR "repeat infection" OR "second infection" OR "future infection" OR "subsequent infection" OR "recurrent infection" OR "prior infection" OR "previous infection" | Completed, Unknown status Studies | COVID-19 OR Coronavirus

Appendix 3. Additional methodological details

Detailed inclusion criteria

**Population**	Humans of any age, in any geographical setting.
**Exposure group**	Confirmed case of SARS-CoV-2 infection with or without COVID-19 vaccination.<br><br>SARS-CoV-2 infection was defined as a **confirmed case** according to the following criteria, adapted from WHO case definitions[2]: positive nucleic acid amplification test (NAAT) according to laboratory records or self-report, positive SARS-CoV-2 antigen rapid diagnostic test (AgRDT) according to laboratory records or self-report, or a positive serology test from a lab-based assay (i.e., CLIA/ELISA) or an antibody-detecting rapid diagnostic test (Ab-RDT).<br><br>Studies were included if they reported on individuals with previously confirmed infection that had documented vaccination (partial primary series, full primary series, or boosted), as defined in the randomized controlled trials for each vaccine.<br><br>**Partial vaccination was** defined as ≥14 days after a single dose of Pfizer/BioNTech-Comirnaty (BNT162b2), AstraZeneca-Vaxzevria, Moderna-mRNA-1273, or Sinovac-CoronaVac, ≥21 days after a single dose of Sinopharm-BBIBP-CorV or Gamaleja-Sputnik-V, <7 days from the second dose for Pfizer/BioNTech-Comirnaty (BNT162b2), <14 days from the second dose for AstraZeneca-Vaxzevria, Moderna-mRNA-1273, or Sinovac-CoronaVac, and <21 days from the second dose of Sinopharm-BBIBP-CorV or Gamaleja-Sputnik-V.<br><br>**Primary series vaccination was** defined as ≥7 days from the second dose for Pfizer/BioNTech-Comirnaty, ≥14 days from the first dose of Janssen-Ad26.COV2.S ≥14 days from the second dose for AstraZeneca-Vaxzevria, Moderna-mRNA-1273, Sinovac-CoronaVac, or BBIBP-CorV Sinopharm, and ≥21 days from the second dose of Gamaleja-Sputnik-V.<br><br>**Booster vaccination one** was defined as ≥7 days from an additional dose after primary series vaccination.
**Comparison group**	Five comparison groups were eligible:<br>(1) no previous vaccinations and no previously confirmed SARS-CoV-2 infection defined using WHO criteria;<br>(2) previously SARS-CoV-2 infection defined using WHO criteria (defined above);<br>(3) partial primary series vaccination (defined above);<br>(4) full primary series vaccination (defined above);<br>(5) booster vaccination (defined above).
**Outcome**	SARS-CoV-2 Omicron reinfection was defined as a possible, probable, or confirmed reinfection case according to the following criteria, adapted from WHO case definitions.<br><br>**Possible reinfection case** was defined as a NAAT or AgRDT SARS-CoV-2 positive case with a history of a primary SARS-CoV-2 infection diagnosed by serology, with at least 60 days between the positive serology test and the subsequent positive NAAT or AgRDT.<br><br>**Probable reinfection case** was defined as a NAAT or AgRDT SARS-CoV-2 positive case with a history of a primary SARS-CoV-2 infection diagnosed by NAAT or AgRDT. At least 90 days must have elapsed between the episodes or, alternatively, genomic evidence for the second episode must be available and include a lineage that was not submitted to SARS-Cov-2 genomic databases at the time of first infection.<br><br>**Confirmed reinfection case** was defined as two PCR positive episodes supported by viral genomic data from both episodes of infection revealing different Pango lineages. If viral genomic data revealed two distinct Pango lineages, this qualified as adequate evidence to confirm reinfection, regardless of the time elapsed between the two episodes.

	**Hospitalization** was defined as any admission to hospital with a confirmed case of SARS-CoV-2, adapted from WHO case definitions.[3]<br><br>**Severe disease** was defined using a combination of the WHO definitions of severe, critical and fatal COVID-19[4,5]:<br>- Severe COVID-19 disease was a SARS-CoV-2 infected person with "oxygen saturation of <90% on room air, and/or respiratory rate of >30 breaths/minute in adults and children >5 years old (or ≥60 breaths/minute in children <2 months old or ≥50 breaths/minute in children 2-11 months old or ≥40 breaths/minute in children 1–5 years old), and/or signs of severe respiratory distress (accessory muscle use and inability to complete full sentences, and, in children, very severe chest wall indrawing, grunting, central cyanosis, or presence of any other general danger signs)".<br>- Critical COVID-19 disease was a SARS-CoV-2 infected person with "acute respiratory distress syndrome, sepsis, septic shock, or other conditions that would normally require the provision of life sustaining therapies such as mechanical ventilation (invasive or non-invasive) or vasopressor therapy".<br>- COVID-19 death was "a death resulting from a clinically compatible illness, in a probable or confirmed COVID-19 case, unless there is a clear alternative cause of death that cannot be related to COVID-19 disease (e.g., trauma). There should be no period of complete recovery from COVID-19 between illness and death. A death due to COVID-19 may not be attributed to another disease (e.g., cancer) and should be counted independently of preexisting conditions that are suspected of triggering a severe course of COVID-19".
**Study design**	Test-negative case-control, case-control, cross-sectional, cohort, non-randomized controlled trials, and randomized controlled trials.
**Type of literature**	Published peer-reviewed research articles, preprints, and grey literature in any language. We will prioritize peer-reviewed versions of articles for inclusion and analysis in instances where pre-print versions of peer-reviewed articles are available.

Detailed exclusion criteria

**Population**	N/A
**Exposure group**	No evidence of prior infection (defined above). No information on the timing, brand, or dose number for the vaccination in hybrid immunity studies.
**Comparison group**	N/A
**Outcome**	Pre-Omicron reinfection. Prior infection studies that did not report the period of time between primary infection and reinfection. Hybrid immunity studies that did not report the period of time between either the determination of primary infection or vaccination and reinfection. Articles reporting identical information to previously included articles.
**Study design**	Case reports, case series, incomplete randomized controlled trials, and review papers. Pre-print version of articles subsequently published in peer-reviewed journals.
**Type of literature**	Media, news stories, and conference abstracts.

Requesting data from authors

Authors were contacted by email to request the data if relevant data were presented in figures but not reported numerically, if data for Omicron was reported in combination with another variant of concern, or if different levels of hybrid immunity were reported in combination (i.e., partial, primary series, first booster, second booster).

Article, study, and estimate definitions

We defined an article as a published peer-reviewed or pre-print manuscript including any appendices or supplementary files. We defined a study as an investigation reported in an article that involved a single set of criteria defining participant inclusion and exclusion, applied a defined sampling strategy to a particular population, and

provided estimates unique to that population at one time point or over time. An article was deemed to report multiple studies if it described two or more investigations that used different sets of participant inclusion or exclusion criteria, applied a different sampling strategy to the same population, or applied the same sampling strategy to different populations and reported non-pooled estimates unique to each population. An estimate was defined as a numerical value calculated based on the events observed in the sample being studied and intended to represent a population parameter.

Outcome definition and comparison groups

Estimates of vaccine effectiveness (i.e., vaccine vs. unvaccinated) against Omicron variant were obtained from a recent systematic and meta-regression to compare to estimates of the protective effectiveness of prior infection and protective effectiveness of hybrid immunity generated in our analysis.[6] The vaccine effectiveness systematic review used similar inclusion and exclusion criteria to our review. The raw analysis dataset used in the vaccine effectiveness paper was obtained by contacting the corresponding author. The dataset contained the "primary series vs unvaccinated" comparison data and "first booster dose vs unvaccinated" comparison data. We applied our meta-regression model to the dataset to project the trends of vaccine effectiveness protection waning, in parallel to trends of prior infection effectiveness and hybrid immunity effectiveness waning that were generated from data procured in our review. Results are shown in figure 3.

Data extraction

All reviewers completed data extraction training which included completion of two pilot extractions with feedback from a trained reviewer that independently extracted the same articles. Team meetings were held twice per week during the extraction phase to discuss and resolve data extraction challenges.

Analysis

To model waning protection over time, we used log-odds meta-regression to bound protection between 0% and 100% and to translate non-linear waning on a percentage scale as linear waning on the log-odds scale. In this model, we regressed the log-odds of protective effectiveness and comparative protective effectiveness on the mean time since the last immunological challenge (i.e., last vaccine dose or infection).[7] For studies reporting risk ratios or hazard ratios, we converted them to the odds ratio.[8] Our model also included a random intercept for each study, shared across all estimates of protection used from that study. Hybrid immunity analyses did not consider the order in which the immunity status was conferred (e.g., vaccination then infection or vice versa). We extracted data for each available time point and identified the mean time since the last immunological challenge. We then regressed the log-odds of protective effectiveness and comparative protective effectiveness on months since the last immunological challenge.[7]

When calculating effect measures involving hybrid immunity the time points for the exposure group was determined based on the most recently documented immunological challenge (i.e., vaccination or infection) prior to the period assessed for reinfection. Hybrid immunity analyses did not consider the order in which the immunity status was conferred (e.g., vaccination then infection or vice versa).

We did not differentiate between Omicron sub-variants.

Protocol deviations

There were two deviations from our protocol which were enacted to expand the scope of eligible data for inclusion:

1) The list of eligible types of vaccination was expanded to include any type of vaccination.
2) We did not restrict inclusion based on the accuracy of rapid diagnostic tests for confirming the index SARS-CoV-2 infection. Instead, the accuracy of the rapid diagnostic tests was considered as part of our risk of bias assessment.

Appendix 4. Detailed list of comparisons with corresponding effect measures and epidemiological and policy questions

Epidemiological and policy questions	Effect measures	Definition	Potential comparisons for analysis[a]
How much protection is conferred by prior infection?	Protective effectiveness of prior infection	The protective effectiveness of prior infection was 1 - odds ratio for reinfection derived by comparing previously infected (I1) unvaccinated (V0) individuals versus previously uninfected (I0) unvaccinated (V0) individuals.	Prior infection vs Naive: V0/I1 vs. V0/I0
How much protection is conferred by hybrid immunity involving partial primary series, full primary series, or booster vaccination?	Protective effectiveness of hybrid immunity	The protective effectiveness of hybrid immunity was 1 - odds ratio for reinfection derived by comparing individuals with hybrid immunity from previous infection (I1) and partial primary series (VP), full primary series (VF), or booster (VB) vaccination versus previously uninfected (I0) unvaccinated (V0) individuals.	Hybrid with partial primary series vs Naive: VP/I1 vs. V0/I0<br><br>Hybrid with full primary series vs Naive: VF/I1 vs. V0/I0<br><br>Hybrid with first booster vs Naive: VB/I1 vs. V0/I0
Do individuals with hybrid immunity have greater protection compared to individuals with prior infection alone?	Comparative protective effectiveness of hybrid immunity relative to prior infection alone	The comparative protective effectiveness of hybrid immunity relative to prior infection alone was1 - odds ratio for reinfection derived by comparing individuals with hybrid immunity from previous infection (I1) and partial primary series (VP), full primary series (VF), or booster (VB) vaccination versus previously infected (I1) unvaccinated (V0) individuals.	Hybrid with partial primary series vs Infection: VP/I1 vs. V0/I1<br><br>Hybrid with full primary series hybrid vs Infection: VF/I1 vs. V0/I1<br><br>Hybrid with first booster vs Infection: VB/I1 vs. V0/I1
Do individuals with hybrid immunity have greater protection compared to individuals with prior vaccination alone?	Comparative protective effectiveness of hybrid immunity relative to prior vaccination only	The comparative protective effectiveness of hybrid immunity relative to prior vaccination alone was 1 - odds ratio for reinfection derived by comparing individuals with hybrid immunity from previous infection (I1) and partial primary series (VP), full primary series (VF), or booster (VB) vaccination versus previously uninfected (I1) individuals with partial primary series (VP) or full primary series (VF) vaccination.	Hybrid with partial primary series vs. Partial primary series: VP/I1 vs. VP/I0<br><br>Hybrid with partial primary series vs. Full primary series: VP/I1 vs. VF/I0<br><br>Hybrid with partial primary series vs. First booster: VP/I1 vs. VB/I0<br><br>Hybrid with full primary series vs. Full primary series: VF/I1 vs. VF/I0<br><br>Hybrid with full primary series vs. First booster: VF/I1 vs. VB/I0<br><br>Hybrid with first booster vs. First booster: VB/I1 vs. VB/I0
Do individuals with hybrid immunity and a greater number of vaccine doses have more protection compared to individuals with hybrid immunity and fewer vaccine doses?	Comparative protective effectiveness of hybrid immunity with more vaccine doses relative to hybrid immunity with fewer vaccine doses	The comparative protective effectiveness of hybrid immunity with more vaccine doses relative to hybrid immunity with fewer vaccine doses was 1 - odds ratio for reinfection derived by comparing individuals with hybrid immunity from previous infection (I1) and primary series (VF) or booster (VB) vaccination versus individuals with hybrid immunity from previous infection (I1) and partial primary series (VP) or full primary series (VF) vaccination.	Hybrid with full primary series vs. Hybrid with partial primary series: VF/I1 vs. VP/I1<br><br>Hybrid with first booster vs. Hybrid with primary series: VB/I1 vs. VF/I1<br><br>Hybrid with first booster vs. Hybrid with partial primary series: VB/I1 vs. VP/I1

[a]Prior infection abbreviations: previously infected - I1; never infected - I0. Vaccination abbreviations: unvaccinated - V0; partial primary series vaccination - VP; full primary series vaccination - VF; first booster vaccination - VB. The notation when combining immunity status for prior infection and vaccination was a slash (/), which did not imply an order of the events (e.g., full primary series vaccination and prior infection was denoted as VF/I1).

Appendix 5. Characteristics and results of individual prior infection studies included in meta-analysis

Author (Country)	Study Design (Variables controlled for in the prior infection protection estimates)	Severity of Infection	Reinfection variant	Prior Infected Variant	Days since Prior Infection/ Vaccination Completion (Median [Range])	Age Range	Protection (Prior infection vs. Immune naive) [95% CI]	Risk of Bias
Altarawneh 1 (Qatar)[9]	Test-negative case-control (Matched cohorts. Adjusted for presence of co-morbidities)	Any Infection	Omicron	Mixed variant	180 [90-269]	All ages	64·0% [54·7-71·4%]	Serious
		Any Infection	Omicron	Mixed variant	360 [270-449]	All ages	47·2% [37·5-55·4%]	Serious
		Any Infection	Omicron	Mixed variant	450	All ages	59·6% [50·7-67%]	Serious
		Hospitalization or severe disease	Omicron	Mixed variant	314	All ages	87·8% [47·5-97·1%]	Moderate
Altarawneh 2 (Qatar)[10]	Test-negative case-control (Matched cohorts. Adjusted for sex, age group, nationality, and calendar week of PCR test)	Any Infection	Omicron	Mixed variant	324	All ages	44·9% [39·2-50·1%]	Moderate
		Any Infection	Omicron (BA·1)	Mixed variant	324	All ages	50·2% [38·1-59·9%]	Moderate
		Any Infection	Omicron (BA·2)	Mixed variant	324	All ages	46·1% [39·5-51·9%]	Moderate
		Hospitalization or severe disease	Omicron	Mixed variant	324	All ages	85·5% [49·1-95·9%]	Moderate
		Hospitalization or severe disease	Omicron (BA·1)	Mixed variant	324	All ages	92·7% [1·0-99·6%]	Moderate
		Hospitalization or severe disease	Omicron (BA·2)	Mixed variant	324	All ages	73·4% [0·2-92·9%]	Moderate
Andeweg (Netherlands)[11]	Test-negative case-control (Adjusted for age, sex, health region, and testing date)	Any Infection	Omicron (BA·1)	Index-Delta	104 [90-119]	18-100	57% [52-62%]	Serious
		Any Infection	Omicron (BA·1)	Index-Delta	134 [120-149]	18-100	55% [46-62%]	Serious
		Any Infection	Omicron (BA·1)	Index-Delta	164 [150-179]	18-100	53% [43-60%]	Serious
		Any Infection	Omicron (BA.1)	Index-Delta	194 [180-209]	18-100	52% [45-58%]	Serious
		Any Infection	Omicron (BA.1)	Index-Delta	210	18-100	34% [31-38%]	Serious
		Any Infection	Omicron (BA.1)	Index-Delta	104 [90-119]	18-100	55% [48-60%]	Serious
		Any Infection	Omicron (BA.1)	Index-Delta	134 [120-149]	18-100	51% [41-60%]	Serious
		Any Infection	Omicron (BA.1)	Index-Delta	164 [150-179]	18-100	54% [44-62%]	Serious
		Any Infection	Omicron (BA.1)	Index-Delta	194 [180-209]	18-100	53% [45-59%]	Serious
		Any Infection	Omicron (BA.1)	Index-Delta	210	18-100	37% [33-40%]	Serious
		Any Infection	Omicron (BA.2)	Index-Delta	104 [90-119]	18-100	50% [43-56%]	Serious
		Any Infection	Omicron (BA.2)	Index-Delta	134 [120-149]	18-100	57% [49-64%]	Serious
		Any Infection	Omicron (BA.2)	Index-Delta	164 [150-179]	18-100	50% [36-61%]	Serious
		Any Infection	Omicron (BA.2)	Index-Delta	194 [180-209]	18-100	53% [42-62%]	Serious

		Any Infection	Omicron (BA.2)	Index-Delta	210	18-100	38% [34-43%]	Serious
		Any Infection	Omicron (BA.2)	Index-Delta	104 [90-119]	18-100	50% [43-56%]	Serious
		Any Infection	Omicron (BA.2)	Index-Delta	134 [120-149]	18-100	58% [50-66%]	Serious
		Any Infection	Omicron (BA.2)	Index-Delta	164 [150-179]	18-100	55% [41-65%]	Serious
		Any Infection	Omicron (BA.2)	Index-Delta	194 [180-209]	18-100	52% [40-62%]	Serious
		Any Infection	Omicron (BA.2)	Index-Delta	210	18-100	40% [35-44%]	Serious
		Any Infection	Omicron (BA.1)	Index-Delta	104 [90-119]	18-100	42% [1-68%]	Serious
		Any Infection	Omicron (BA.1)	Index-Delta	134 [120-149]	18-100	31% [1-54%]	Serious
		Any Infection	Omicron (BA.1)	Index-Delta	164 [150-179]	18-100	28% [8-43%]	Serious
		Any Infection	Omicron (BA.1)	Index-Delta	194 [180-209]	18-100	12% [1-43%]	Serious
		Any Infection	Omicron (BA.1)	Index-Delta	210	18-100	6% [1-16%]	Serious
		Any Infection	Omicron (BA.1)	Index-Delta	104 [90-119]	18-100	52% [3-77%]	Serious
		Any Infection	Omicron (BA.1)	Index-Delta	134 [120-149]	18-100	24% [1-51%]	Serious
		Any Infection	Omicron (BA.1)	Index-Delta	164 [150-179]	18-100	22% [1-40%]	Serious
		Any Infection	Omicron (BA.1)	Index-Delta	194 [180-209]	18-100	1% [1-35%]	Serious
		Any Infection	Omicron (BA.1)	Index-Delta	210	18-100	5% [1-16%]	Serious
Carazo (Canada)[12]	Test-negative case-control (Adjusted for age, sex, testing-indication and epi-week but not comorbidity)	Any Infection	Omicron	Alpha (B.1.1.7),Beta,Gamma,Delta	134 [90-179]	12-100	67% [57-74%]	Serious
		Any Infection	Omicron	Alpha (B.1.1.7),Beta,Gamma,Delta	455 [180-730]	12-100	37% [29-43%]	Serious
		Any Infection	Omicron	Alpha (B.1.1.7),Beta,Gamma,Delta	136 [90-182]	12-100	66% [57-73%]	Serious
		Any Infection	Omicron	Alpha (B.1.1.7),Beta,Gamma,Delta	228 [183-274]	12-100	49% [32-61%]	Serious
		Any Infection	Omicron	Alpha (B.1.1.7),Beta,Gamma,Delta	320 [275-364]	12-100	35% [21-47%]	Serious
		Any Infection	Omicron	Alpha (B.1.1.7),Beta,Gamma,Delta	456 [365-547]	12-100	29% [17-38%]	Serious
		Any Infection	Omicron	Alpha (B.1.1.7),Beta,Gamma,Delta	639 [548-730]	12-100	27% [8-42%]	Serious
		Any Infection	Omicron	Alpha (B.1.1.7),Beta,Gamma,Delta	134 [90-179]	12-100	49% [8-72%]	Serious

		Any Infection	Omicron	Alpha (B.1.1.7),Beta,Gamma,Delta	455 [180-730]	12-100	1% [1-20%]	Serious
		Hospitalization or severe disease	Omicron	Alpha (B.1.1.7),Beta,Gamma,Delta	134 [90-179]	12-100	81% [52-92%]	Moderate
		Hospitalization or severe disease	Omicron	Alpha (B.1.1.7),Beta,Gamma,Delta	455 [180-730]	12-100	62% [36-77%]	Moderate
Cerqueira-Silva (Brazil)[13]	Test-negative case-control (Matched cohorts. Adjusted for matched design, comorbidities, pregnancy, race, days elapsed between tests, hospital admission, and age)	Any Infection	Omicron	Alpha (B.1.1.7),Delta (B.1.617.2),Gamma	134 [90-179]	18-100	52·8% [48·3-56·8%]	Moderate
		Any Infection	Omicron	Alpha (B.1.1.7),Delta (B.1.617.2),Gamma	272 [180-365]	18-100	32·7% [30·2-35·2%]	Moderate
		Any Infection	Omicron	Alpha (B.1.1.7),Delta (B.1.617.2),Gamma	365	18-100	14·7% [10·8-18·5%]	Moderate
		Hospitalization or severe disease	Omicron	Alpha (B.1.1.7),Delta (B.1.617.2),Gamma	134 [90-179]	18-100	84·5% [73·1-91·1%]	Serious
		Hospitalization or severe disease	Omicron	Alpha (B.1.1.7),Delta (B.1.617.2),Gamma	272 [180-365]	18-100	89·5% [86-92·2%]	Serious
		Hospitalization or severe disease	Omicron	Alpha (B.1.1.7),Delta (B.1.617.2),Gamma	365	18-100	80·3% [74·4-84·8%]	Serious
Chin (USA)[14]	Test-negative case-control (Matched cohorts. Adjusted for age group, and sex)	Any Infection	Omicron	Delta (B.1.617.2)	150	18-100	54·2% [41·4-66·2%]	Moderate
		Any Infection	Omicron	Delta (B.1.617.2)	154	18-100	60·9% [58·5-67·8%]	Moderate
Lind (USA)[15]	Test-negative case-control (Adjusted for date of test, age (continuous), sex, race/ethnicity, comorbidity score, clinical encounters, insurance group, and regional social vulnerability)	Any Infection	Omicron	Mixed variant	328	5-100	25·3% [16·1-33·5%]	Moderate
Michlmayr (Denmark)[16]	Cohort (Adjusted age, sex, comorbidity, region of affiliation and staying at hospital, vaccination status, and time since vaccination)	Any Infection	Omicron	Mixed variant	134 [90-179]	2-100	41·7% [40·8-42·6%]	Serious
		Any Infection	Omicron	Mixed variant	224 [180-269]	2-100	18·8% [17·1-20·3%]	Serious
		Any Infection	Omicron	Mixed variant	314 [270-359]	2-100	18·5% [16·9-20·1%]	Serious
		Any Infection	Omicron	Mixed variant	360	2-100	13·7% [12·3-14·8%]	Serious
		Any Infection	Omicron	Mixed variant	134 [90-179]	2-100	48·1% [46·3-49·9%]	Serious
		Any Infection	Omicron	Mixed variant	224 [180-269]	2-100	25·3% [22·5-27·9%]	Serious
		Any Infection	Omicron	Mixed variant	314 [270-359]	2-100	25·6% [22·9-28·2%]	Serious
		Any Infection	Omicron	Mixed variant	360	2-100	20·6% [18·1-22·9%]	Serious

		Hospitalization or severe disease	Omicron	Mixed variant	90	2-100	69·8% [51·5-81·2%]	Serious
Nyberg (UK)[17]	Retrospective cohort (Adjusted for age, sex, and index of multiple deprivation)	Hospitalization or severe disease	Omicron	Index-Delta	180	18-100	45% [37-52%]	Serious
		Hospitalization or severe disease	Omicron	Index-Delta	180	18-100	32% [26-38%]	Serious
		Hospitalization or severe disease	Omicron	Index-Delta	180	18-100	28% [22-33%]	Serious
		Hospitalization or severe disease	Omicron	Index-Delta	180	18-100	82% [43-94%]	Serious
		Hospitalization or severe disease	Omicron	Index-Delta	180	18-100	82% [76-89%]	Serious
		Hospitalization or severe disease	Omicron	Index-Delta	180	18-100	93% [88-97%]	Serious
		Hospitalization or severe disease	Omicron	Index-Delta	180	18-100	94% [90-99%]	Serious
		Hospitalization or severe disease	Omicron	Index-Delta	180	18-100	34% [22-54%]	Serious
		Hospitalization or severe disease	Omicron	Index-Delta	180	18-100	69% [55-88%]	Serious
		Hospitalization or severe disease	Omicron	Index-Delta	180	18-100	84% [72-99%]	Serious
		Hospitalization or severe disease	Omicron	Index-Delta	180	18-100	86% [74-100%]	Serious
		Hospitalization or severe disease	Omicron	Index-Delta	180	18-100	48% [21-100%]	Serious
		Hospitalization or severe disease	Omicron	Index-Delta	180	18-100	54% [47-62%]	Serious
		Hospitalization or severe disease	Omicron	Index-Delta	180	18-100	37% [29-44%]	Serious
		Hospitalization or severe disease	Omicron	Index-Delta	180	18-100	32% [25-40%]	Serious
		Hospitalization or severe disease	Omicron	Index-Delta	180	18-100	90% [78-100%]	Serious
Šmíd (Czechia)[18]	Cross-sectional (Adjusted for age group, sex, and calendar time)	Any Infection	Omicron	Delta (B.1.617.2)	120 [60-180]	All ages	66·3% [66·3-67·3%]	Moderate
		Any Infection	Omicron	Delta (B.1.617.2)	120 [60-180]	All ages	69% [68-69%]	Moderate
		Any Infection	Omicron	Wild-type,Alpha (B.1.1.7)	241 [181-301]	All ages	48% [46-50%]	Moderate
		Any Infection	Omicron	Wild-type,Alpha (B.1.1.7)	362 [302-422]	All ages	34% [33-35%]	Moderate
		Any Infection	Omicron	Wild-type,Alpha (B.1.1.7)	423	All ages	17% [15-18%]	Moderate
		Any Infection	Omicron	Wild-type,Alpha (B.1.1.7)	302 [181-423]	All ages	12·1% [10·3-13·1%]	Moderate
		Hospitalization or severe disease	Omicron	Delta (B.1.617.2)	120 [60-180]	All ages	73% [55-84%]	Moderate
		Hospitalization or severe disease	Omicron	Wild-type,Alpha (B.1.1.7)	302 [181-423]	All ages	66% [54-75%]	Moderate
		Hospitalization or severe disease	Omicron	Delta (B.1.617.2)	120 [60-180]	All ages	80·8% [40-93·9%]	Moderate
		Hospitalization or severe disease	Omicron	Wild-type,Alpha (B.1.1.7)	302 [181-423]	All ages	87·6% [72·2-94·5%]	Moderate
		Hospitalization or severe disease	Omicron	Delta (B.1.617.2)	120 [60-180]	All ages	82·8% [1-97·6%]	Moderate
		Hospitalization or severe disease	Omicron	Wild-type,Alpha (B.1.1.7)	302 [181-423]	All ages	65·7% [15-86·1%]	Moderate

Appendix 6. Characteristics and results of individual hybrid immunity studies included in meta-analysis

Author (Country)	Study Design (Variables controlled for in the hybrid immunity protection estimates)	Exposure vs Comparator	Vaccine	Severity of Infection	Reinfection Variant	Prior Infected Variant	Days since Prior Infection/ Vaccination Completion (Median [Range])	Age Range	Protection [95% CI]	Risk of Bias
Altarawneh 2 (Qatar)(10)	Test-negative case-control (Matched cohorts. Adjusted for sex, age group, nationality, and calendar week of PCR test)	Infection + 1st booster vaccine vs naive	Pfizer/BioNTech-Comirnaty	Any Infection	Omicron (BA.1)	Mixed variant	42	0-100	74·4% [63·4-82·2%]	Moderate
		Infection + 1st booster vaccine vs naive	Pfizer/BioNTech-Comirnaty	Any Infection	Omicronn (BA.2)	Mixed variant	43	0-100	77·3% [72·4-81·4%]	Moderate
		Infection + 1st booster vaccine vs naive	Pfizer/BioNTech-Comirnaty	Hospitalization or severe disease	Omicronn (BA.2)	Mixed variant	43	0-100	97·5% [57·6-99·9%]	Moderate
		Infection + full primary series vaccine vs naive	Pfizer/BioNTech-Comirnaty	Any Infection	Omicronn (BA.1)	Mixed variant	268	0-100	51·7% [43·5-58·7%]	Moderate
		Infection + full primary series vaccine vs naive	Pfizer/BioNTech-Comirnaty	Any Infection	Omicronn (BA.2)	Mixed variant	270	0-100	55·1% [50·9-58·9%]	Moderate
		Infection + full primary series vaccine vs naive	Pfizer/BioNTech-Comirnaty	Hospitalization or severe disease	Omicronn (BA.2)	Mixed variant	270	0-100	97·8% [82·6-99·7%]	Moderate
		Infection + full primary series vaccine vs naive	Pfizer/BioNTech-Comirnaty	Hospitalization or severe disease	Omicronn (BA.1)	Mixed variant	268	0-100	96·2% [37·7-99·8%]	Moderate
Andeweg (Netherlands) (11)	Test-negative case-control (Adjusted for age, sex, health region, and testing date)	Infection + 1st booster vaccine vs naive	Pfizer/BioNTech-Comirnaty,Moderna-mRNA-1273,AstraZeneca-Vaxzevria,Janssen-Ad26.COV2.S	Any Infection	Omicron (BA.1)	Mixed variant	104 [90-119]	18-100	66% [46-79%]	Serious
		Infection + 1st booster vaccine vs naive	Pfizer/BioNTech-Comirnaty,Moderna-mRNA-1273,AstraZeneca-Vaxzevria,Janssen-Ad26.COV2.S	Any Infection	Omicron (BA.1)	Mixed variant	104 [90-119]	18-100	70% [50-82%]	Serious
		Infection + 1st booster vaccine vs naive	Pfizer/BioNTech-Comirnaty,Moderna-mRNA-1273,AstraZeneca-Vaxzevria,Janssen-Ad26.COV2.S	Any Infection	Omicron (BA.2)	Mixed variant	104 [90-119]	18-100	75% [67-81%]	Serious
		Infection + 1st booster vaccine vs naive	Pfizer/BioNTech-Comirnaty,Moderna-mRNA-1273,AstraZeneca-Vaxzevria,Janssen-Ad26.COV2.S	Any Infection	Omicron (BA.2)	Mixed variant	104 [90-119]	18-100	74% [66-80%]	Serious

		Infection + full primary series vaccine vs naive	Pfizer/BioNTech-Comirnaty,Moderna-mRNA-1273,AstraZeneca-Vaxzevria,Janssen-Ad26.COV2.S	Any Infection	Omicron (BA.1)	Delta (B.1.617.2)	134 [120-149]	18-100	85% [82-87%]	Serious
		Infection + full primary series vaccine vs naive	Pfizer/BioNTech-Comirnaty,Moderna-mRNA-1273,AstraZeneca-Vaxzevria,Janssen-Ad26.COV2.S	Any Infection	Omicron (BA.1)	Delta (B.1.617.2)	164 [150-179]	18-100	81% [75-86%]	Serious
		Infection + full primary series vaccine vs naive	Pfizer/BioNTech-Comirnaty,Moderna-mRNA-1273,AstraZeneca-Vaxzevria,Janssen-Ad26.COV2.S	Any Infection	Omicron (BA.1)	Delta (B.1.617.2)	104 [90-119]	18-100	66% [53-75%]	Serious
		Infection + full primary series vaccine vs naive	Pfizer/BioNTech-Comirnaty,Moderna-mRNA-1273,AstraZeneca-Vaxzevria,Janssen-Ad26.COV2.S	Any Infection	Omicron (BA.1)	Delta (B.1.617.2)	134 [120-149]	18-100	67% [52-77%]	Serious
		Infection + full primary series vaccine vs naive	Pfizer/BioNTech-Comirnaty,Moderna-mRNA-1273,AstraZeneca-Vaxzevria,Janssen-Ad26.COV2.S	Any Infection	Omicron (BA.1)	Delta (B.1.617.2)	164 [150-179]	18-100	68% [50-79%]	Serious
		Infection + full primary series vaccine vs naive	Pfizer/BioNTech-Comirnaty,Moderna-mRNA-1273,AstraZeneca-Vaxzevria,Janssen-Ad26.COV2.S	Any Infection	Omicron (BA.1)	Delta (B.1.617.2)	194 [180-209]	18-100	82% [79-85%]	Serious
		Infection + full primary series vaccine vs naive	Pfizer/BioNTech-Comirnaty,Moderna-mRNA-1273,AstraZeneca-Vaxzevria,Janssen-Ad26.COV2.S	Any Infection	Omicron (BA.1)	Delta (B.1.617.2)	210	18-100	82% [75-88%]	Serious
		Infection + full primary series vaccine vs naive	Pfizer/BioNTech-Comirnaty,Moderna-mRNA-1273,AstraZeneca-Vaxzevria,Janssen-Ad26.COV2.S	Any Infection	Omicron (BA.1)	Delta (B.1.617.2)	104 [90-119]	18-100	66% [52-76%]	Serious
		Infection + full primary series vaccine vs naive	Pfizer/BioNTech-Comirnaty,Moderna-mRNA-1273,AstraZeneca-Vaxzevria,Janssen-Ad26.COV2.S	Any Infection	Omicron (BA.1)	Delta (B.1.617.2)	134 [120-149]	18-100	69% [52-80%]	Serious
		Infection + full primary series vaccine vs naive	Pfizer/BioNTech-Comirnaty,Moderna-mRNA-1273,AstraZeneca-Vaxzevria,Janssen-Ad26.COV2.S	Any Infection	Omicron (BA.1)	Delta (B.1.617.2)	164 [150-179]	18-100	72% [54-83%]	Serious

		Infection + full primary series vaccine vs naive	Pfizer/BioNTech-Comirnaty,Moderna-mRNA-1273,AstraZeneca-Vaxzevria,Janssen-Ad26.COV2.S	Any Infection	Omicron (BA.2)	Delta (B.1.617.2)	194 [180-209]	18-100	84% [81-86%]	Serious
		Infection + full primary series vaccine vs naive	Pfizer/BioNTech-Comirnaty,Moderna-mRNA-1273,AstraZeneca-Vaxzevria,Janssen-Ad26.COV2.S	Any Infection	Omicron (BA.2)	Delta (B.1.617.2)	210	18-100	85% [80-89%]	Serious
		Infection + full primary series vaccine vs naive	Pfizer/BioNTech-Comirnaty,Moderna-mRNA-1273,AstraZeneca-Vaxzevria,Janssen-Ad26.COV2.S	Any Infection	Omicron (BA.2)	Delta (B.1.617.2)	104 [90-119]	18-100	77% [66-85%]	Serious
		Infection + full primary series vaccine vs naive	Pfizer/BioNTech-Comirnaty,Moderna-mRNA-1273,AstraZeneca-Vaxzevria,Janssen-Ad26.COV2.S	Any Infection	Omicron (BA.2)	Delta (B.1.617.2)	194 [180-209]	18-100	64% [43-77%]	Serious
		Infection + full primary series vaccine vs naive	Pfizer/BioNTech-Comirnaty,Moderna-mRNA-1273,AstraZeneca-Vaxzevria,Janssen-Ad26.COV2.S	Any Infection	Omicron (BA.2)	Delta (B.1.617.2)	210	18-100	48% [26-64%]	Serious
		Infection + full primary series vaccine vs naive	Pfizer/BioNTech-Comirnaty,Moderna-mRNA-1273,AstraZeneca-Vaxzevria,Janssen-Ad26.COV2.S	Any Infection	Omicron (BA.2)	Delta (B.1.617.2)	194 [180-209]	18-100	84% [81-87%]	Serious
		Infection + full primary series vaccine vs naive	Pfizer/BioNTech-Comirnaty,Moderna-mRNA-1273,AstraZeneca-Vaxzevria,Janssen-Ad26.COV2.S	Any Infection	Omicron (BA.2)	Delta (B.1.617.2)	210	18-100	85% [80-89%]	Serious
		Infection + full primary series vaccine vs naive	Pfizer/BioNTech-Comirnaty,Moderna-mRNA-1273,AstraZeneca-Vaxzevria,Janssen-Ad26.COV2.S	Any Infection	Omicron (BA.2)	Delta (B.1.617.2)	104 [90-119]	18-100	76% [63-84%]	Serious
		Infection + full primary series vaccine vs naive	Pfizer/BioNTech-Comirnaty,Moderna-mRNA-1273,AstraZeneca-Vaxzevria,Janssen-Ad26.COV2.S	Any Infection	Omicron (BA.2)	Delta (B.1.617.2)	134 [120-149]	18-100	65% [43-78%]	Serious
		Infection + full primary series vaccine vs naive	Pfizer/BioNTech-Comirnaty,Moderna-mRNA-1273,AstraZeneca-Vaxzevria,Janssen-Ad26.COV2.S	Any Infection	Omicron (BA.2)	Delta (B.1.617.2)	164 [150-179]	18-100	51% [28-67%]	Serious

		Infection + full primary series vaccine vs naive	Pfizer/BioNTech-Comirnaty,Moderna-mRNA-1273,AstraZeneca-Vaxzevria,Janssen-Ad26.COV2.S	Any Infection	Omicron (BA.1)	Wild-type,Alpha (B.1.1.7),Delta (B.1.617.2)	194 [180-209]	18-100	68% [57-75%]	Serious
		Infection + full primary series vaccine vs naive	Pfizer/BioNTech-Comirnaty,Moderna-mRNA-1273,AstraZeneca-Vaxzevria,Janssen-Ad26.COV2.S	Any Infection	Omicron (BA.1)	Wild-type,Alpha (B.1.1.7),Delta (B.1.617.2)	210	18-100	60% [44-71%]	Serious
		Infection + full primary series vaccine vs naive	Pfizer/BioNTech-Comirnaty,Moderna-mRNA-1273,AstraZeneca-Vaxzevria,Janssen-Ad26.COV2.S	Any Infection	Omicron (BA.1)	Wild-type,Alpha (B.1.1.7),Delta (B.1.617.2)	104 [90-119]	18-100	61% [47-71%]	Serious
		Infection + full primary series vaccine vs naive	Pfizer/BioNTech-Comirnaty,Moderna-mRNA-1273,AstraZeneca-Vaxzevria,Janssen-Ad26.COV2.S	Any Infection	Omicron (BA.1)	Wild-type,Alpha (B.1.1.7),Delta (B.1.617.2)	134 [120-149]	18-100	56% [46-64%]	Serious
		Infection + full primary series vaccine vs naive	Pfizer/BioNTech-Comirnaty,Moderna-mRNA-1273,AstraZeneca-Vaxzevria,Janssen-Ad26.COV2.S	Any Infection	Omicron (BA.1)	Wild-type,Alpha (B.1.1.7),Delta (B.1.617.2)	164 [150-179]	18-100	63% [55-70%]	Serious
		Infection + full primary series vaccine vs naive	Pfizer/BioNTech-Comirnaty,Moderna-mRNA-1273,AstraZeneca-Vaxzevria,Janssen-Ad26.COV2.S	Any Infection	Omicron (BA.1)	Wild-type,Alpha (B.1.1.7),Delta (B.1.617.2)	194 [180-209]	18-100	68% [56-77%]	Serious
		Infection + full primary series vaccine vs naive	Pfizer/BioNTech-Comirnaty,Moderna-mRNA-1273,AstraZeneca-Vaxzevria,Janssen-Ad26.COV2.S	Any Infection	Omicron (BA.1)	Wild-type,Alpha (B.1.1.7),Delta (B.1.617.2)	134 [120-149]	18-100	53% [32-67%]	Serious
		Infection + full primary series vaccine vs naive	Pfizer/BioNTech-Comirnaty,Moderna-mRNA-1273,AstraZeneca-Vaxzevria,Janssen-Ad26.COV2.S	Any Infection	Omicron (BA.1)	Wild-type,Alpha (B.1.1.7),Delta (B.1.617.2)	164 [150-179]	18-100	52% [32-66%]	Serious
		Infection + full primary series vaccine vs naive	Pfizer/BioNTech-Comirnaty,Moderna-mRNA-1273,AstraZeneca-Vaxzevria,Janssen-Ad26.COV2.S	Any Infection	Omicron (BA.1)	Wild-type,Alpha (B.1.1.7),Delta (B.1.617.2)	104 [90-119]	18-100	52% [39-62%]	Serious
		Infection + full primary series vaccine vs naive	Pfizer/BioNTech-Comirnaty,Moderna-mRNA-1273,AstraZeneca-Vaxzevria,Janssen-Ad26.COV2.S	Any Infection	Omicron (BA.1)	Wild-type,Alpha (B.1.1.7),Delta (B.1.617.2)	134 [120-149]	18-100	63% [54-70%]	Serious

		Infection + full primary series vaccine vs naive	Pfizer/BioNTech-Comirnaty,Moderna-mRNA-1273,AstraZeneca-Vaxzevria,Janssen-Ad26.COV2.S	Any Infection	Omicron (BA.2)	Wild-type,Alpha (B.1.1.7),Delta (B.1.617.2)	164 [150-179]	18-100	76% [64-84%]	Serious
		Infection + full primary series vaccine vs naive	Pfizer/BioNTech-Comirnaty,Moderna-mRNA-1273,AstraZeneca-Vaxzevria,Janssen-Ad26.COV2.S	Any Infection	Omicron (BA.2)	Wild-type,Alpha (B.1.1.7),Delta (B.1.617.2)	194 [180-209]	18-100	69% [52-80%]	Serious
		Infection + full primary series vaccine vs naive	Pfizer/BioNTech-Comirnaty,Moderna-mRNA-1273,AstraZeneca-Vaxzevria,Janssen-Ad26.COV2.S	Any Infection	Omicron (BA.2)	Wild-type,Alpha (B.1.1.7),Delta (B.1.617.2)	210	18-100	69% [50-81%]	Serious
		Infection + full primary series vaccine vs naive	Pfizer/BioNTech-Comirnaty,Moderna-mRNA-1273,AstraZeneca-Vaxzevria,Janssen-Ad26.COV2.S	Any Infection	Omicron (BA.2)	Wild-type,Alpha (B.1.1.7),Delta (B.1.617.2)	104 [90-119]	18-100	62% [46-73%]	Serious
		Infection + full primary series vaccine vs naive	Pfizer/BioNTech-Comirnaty,Moderna-mRNA-1273,AstraZeneca-Vaxzevria,Janssen-Ad26.COV2.S	Any Infection	Omicron (BA.2)	Wild-type,Alpha (B.1.1.7),Delta (B.1.617.2)	134 [120-149]	18-100	67% [58-74%]	Serious
		Infection + full primary series vaccine vs naive	Pfizer/BioNTech-Comirnaty,Moderna-mRNA-1273,AstraZeneca-Vaxzevria,Janssen-Ad26.COV2.S	Any Infection	Omicron (BA.2)	Wild-type,Alpha (B.1.1.7),Delta (B.1.617.2)	164 [150-179]	18-100	77% [64-85%]	Serious
		Infection + full primary series vaccine vs naive	Pfizer/BioNTech-Comirnaty,Moderna-mRNA-1273,AstraZeneca-Vaxzevria,Janssen-Ad26.COV2.S	Any Infection	Omicron (BA.2)	Wild-type,Alpha (B.1.1.7),Delta (B.1.617.2)	194 [180-209]	18-100	67% [47-79%]	Serious
		Infection + full primary series vaccine vs naive	Pfizer/BioNTech-Comirnaty,Moderna-mRNA-1273,AstraZeneca-Vaxzevria,Janssen-Ad26.COV2.S	Any Infection	Omicron (BA.2)	Wild-type,Alpha (B.1.1.7),Delta (B.1.617.2)	210	18-100	65% [40-79%]	Serious
		Infection + full primary series vaccine vs naive	Pfizer/BioNTech-Comirnaty,Moderna-mRNA-1273,AstraZeneca-Vaxzevria,Janssen-Ad26.COV2.S	Any Infection	Omicron (BA.2)	Wild-type,Alpha (B.1.1.7),Delta (B.1.617.2)	104 [90-119]	18-100	62% [44-74%]	Serious
		Infection + full primary series vaccine vs naive	Pfizer/BioNTech-Comirnaty,Moderna-mRNA-1273,AstraZeneca-Vaxzevria,Janssen-Ad26.COV2.S	Any Infection	Omicron (BA.2)	Wild-type,Alpha (B.1.1.7),Delta (B.1.617.2)	134 [120-149]	18-100	67% [57-74%]	Serious

		Infection + full primary series vaccine vs naive	Pfizer/BioNTech-Comirnaty,Moderna-mRNA-1273,AstraZeneca-Vaxzevria,Janssen-Ad26.COV2.S	Any Infection	Omicron (BA.1)	Delta (B.1.617.2)	164 [150-179]	18-100	42% [23-57%]	Serious
		Infection + full primary series vaccine vs naive	Pfizer/BioNTech-Comirnaty,Moderna-mRNA-1273,AstraZeneca-Vaxzevria,Janssen-Ad26.COV2.S	Any Infection	Omicron (BA.1)	Delta (B.1.617.2)	194 [180-209]	18-100	35% [9-53%]	Serious
		Infection + full primary series vaccine vs naive	Pfizer/BioNTech-Comirnaty,Moderna-mRNA-1273,AstraZeneca-Vaxzevria,Janssen-Ad26.COV2.S	Any Infection	Omicron (BA.1)	Delta (B.1.617.2)	210	18-100	39% [20-53%]	Serious
		Infection + full primary series vaccine vs naive	Pfizer/BioNTech-Comirnaty,Moderna-mRNA-1273,AstraZeneca-Vaxzevria,Janssen-Ad26.COV2.S	Any Infection	Omicron (BA.1)	Delta (B.1.617.2)	104 [90-119]	18-100	36% [1-63%]	Serious
		Infection + full primary series vaccine vs naive	Pfizer/BioNTech-Comirnaty,Moderna-mRNA-1273,AstraZeneca-Vaxzevria,Janssen-Ad26.COV2.S	Any Infection	Omicron (BA.1)	Delta (B.1.617.2)	134 [120-149]	18-100	79% [1-97%]	Serious
		Infection + full primary series vaccine vs naive	Pfizer/BioNTech-Comirnaty,Moderna-mRNA-1273,AstraZeneca-Vaxzevria,Janssen-Ad26.COV2.S	Any Infection	Omicron (BA.1)	Delta (B.1.617.2)	164 [150-179]	18-100	41% [14-60%]	Serious
		Infection + full primary series vaccine vs naive	Pfizer/BioNTech-Comirnaty,Moderna-mRNA-1273,AstraZeneca-Vaxzevria,Janssen-Ad26.COV2.S	Any Infection	Omicron (BA.1)	Delta (B.1.617.2)	194 [180-209]	18-100	35% [1-59%]	Serious
		Infection + full primary series vaccine vs naive	Pfizer/BioNTech-Comirnaty,Moderna-mRNA-1273,AstraZeneca-Vaxzevria,Janssen-Ad26.COV2.S	Any Infection	Omicron (BA.1)	Delta (B.1.617.2)	210	18-100	28% [1-48%]	Serious
		Infection + full primary series vaccine vs naive	Pfizer/BioNTech-Comirnaty,Moderna-mRNA-1273,AstraZeneca-Vaxzevria,Janssen-Ad26.COV2.S	Any Infection	Omicron (BA.1)	Delta (B.1.617.2)	164 [150-179]	18-100	1% [1-44%]	Serious
		Infection + full primary series vaccine vs naive	Pfizer/BioNTech-Comirnaty,Moderna-mRNA-1273,AstraZeneca-Vaxzevria,Janssen-Ad26.COV2.S	Any Infection	Omicron (BA.1)	Delta (B.1.617.2)	210	18-100	51% [1-94%]	Serious

		Infection + full primary series vaccine vs naive	Pfizer/BioNTech-Comirnaty,Moderna-mRNA-1273,AstraZeneca-Vaxzevria,Janssen-Ad26.COV2.S	Any Infection	Omicron (BA.1)	Wild-type,Alpha (B.1.1.7),Delta (B.1.617.2)	104 [90-119]	18-100	55% [28-72%]	Serious
		Infection + full primary series vaccine vs naive	Pfizer/BioNTech-Comirnaty,Moderna-mRNA-1273,AstraZeneca-Vaxzevria,Janssen-Ad26.COV2.S	Any Infection	Omicron (BA.1)	Wild-type,Alpha (B.1.1.7),Delta (B.1.617.2)	134 [120-149]	18-100	48% [30-62%]	Serious
		Infection + full primary series vaccine vs naive	Pfizer/BioNTech-Comirnaty,Moderna-mRNA-1273,AstraZeneca-Vaxzevria,Janssen-Ad26.COV2.S	Any Infection	Omicron (BA.1)	Wild-type,Alpha (B.1.1.7),Delta (B.1.617.2)	134 [120-149]	18-100	40% [25-52%]	Serious
		Infection + full primary series vaccine vs naive	Pfizer/BioNTech-Comirnaty,Moderna-mRNA-1273,AstraZeneca-Vaxzevria,Janssen-Ad26.COV2.S	Any Infection	Omicron (BA.1)	Wild-type,Alpha (B.1.1.7),Delta (B.1.617.2)	194 [180-209]	18-100	59% [43-70%]	Serious
		Infection + full primary series vaccine vs naive	Pfizer/BioNTech-Comirnaty,Moderna-mRNA-1273,AstraZeneca-Vaxzevria,Janssen-Ad26.COV2.S	Any Infection	Omicron (BA.1)	Wild-type,Alpha (B.1.1.7),Delta (B.1.617.2)	210	18-100	50% [26-66%]	Serious
		Infection + full primary series vaccine vs naive	Pfizer/BioNTech-Comirnaty,Moderna-mRNA-1273,AstraZeneca-Vaxzevria,Janssen-Ad26.COV2.S	Any Infection	Omicron (BA.1)	Wild-type,Alpha (B.1.1.7),Delta (B.1.617.2)	104 [90-119]	18-100	46% [8-69%]	Serious
		Infection + full primary series vaccine vs naive	Pfizer/BioNTech-Comirnaty,Moderna-mRNA-1273,AstraZeneca-Vaxzevria,Janssen-Ad26.COV2.S	Any Infection	Omicron (BA.1)	Wild-type,Alpha (B.1.1.7),Delta (B.1.617.2)	104 [90-119]	18-100	50% [27-66%]	Serious
		Infection + full primary series vaccine vs naive	Pfizer/BioNTech-Comirnaty,Moderna-mRNA-1273,AstraZeneca-Vaxzevria,Janssen-Ad26.COV2.S	Any Infection	Omicron (BA.1)	Wild-type,Alpha (B.1.1.7),Delta (B.1.617.2)	164 [150-179]	18-100	44% [26-58%]	Serious
		Infection + full primary series vaccine vs naive	Pfizer/BioNTech-Comirnaty,Moderna-mRNA-1273,AstraZeneca-Vaxzevria,Janssen-Ad26.COV2.S	Any Infection	Omicron (BA.1)	Wild-type,Alpha (B.1.1.7),Delta (B.1.617.2)	194 [180-209]	18-100	58% [40-71%]	Serious
		Infection + full primary series vaccine vs naive	Pfizer/BioNTech-Comirnaty,Moderna-mRNA-1273,AstraZeneca-Vaxzevria,Janssen-Ad26.COV2.S	Any Infection	Omicron (BA.1)	Wild-type,Alpha (B.1.1.7),Delta (B.1.617.2)	210	18-100	47% [18-66%]	Serious

Björk (Sweden)(19)	Traditional case-control (Age and sex matched case and control cohorts. Adjusted for for comorbidities)	Infection + full primary series vaccine vs naive	Pfizer/BioNTech-Comirnaty,Moderna-mRNA-1273,AstraZeneca-Vaxzevria	Hospitalization or severe disease	Omicron (BA.1)	Index-Delta	94 [7-180]	0-100	91% [57-98%]	Serious
		Infection + full primary series vaccine vs naive	Pfizer/BioNTech-Comirnaty,Moderna-mRNA-1273,AstraZeneca-Vaxzevria	Hospitalization or severe disease	Omicron (BA.2)	Index-Delta	94 [7-180]	0-100	53% [1-82%]	Serious
		Infection + full primary series vaccine vs naive	Pfizer/BioNTech-Comirnaty,Moderna-mRNA-1273,AstraZeneca-Vaxzevria	Hospitalization or severe disease	Omicron	Index-Delta	94 [7-180]	0-100	92% [59-98%]	Serious
Bruel (France)(20)	Cohort (No adjustment)	Infection + 1st booster vaccine vs 1st booster vaccine	Pfizer/BioNTech-Comirnaty,Moderna-mRNA-1273,	Any Infection	Omicron	Mixed variant	90	72-101	89% [29-98%]	Serious
Carazo (Canada)(12)	Test-negative case-control (Adjusted for age, sex, testing-indication and epi-week but not comorbidity)	Infection + 1st booster vaccine vs naive	Pfizer/BioNTech-Comirnaty,Moderna-mRNA-1273	Any Infection	Omicron	Alpha (B.1.1.7),Beta,Gamma,Delta	7	12-100	83% [81-84%]	Serious
		Infection + 1st booster vaccine vs naive	Pfizer/BioNTech-Comirnaty,Moderna-mRNA-1273	Any Infection	Omicron	Alpha (B.1.1.7),Beta,Gamma,Delta	120 [60-179]	12-100	80% [76-84%]	Serious
		Infection + 1st booster vaccine vs naive	Pfizer/BioNTech-Comirnaty,Moderna-mRNA-1273	Any Infection	Omicron	Alpha (B.1.1.7),Beta,Gamma,Delta	34 [7-60]	12-100	83% [81-84%]	Serious
		Infection + 1st booster vaccine vs naive	Pfizer/BioNTech-Comirnaty,Moderna-mRNA-1273	Hospitalization or severe disease	Omicron	Alpha (B.1.1.7),Beta,Gamma,Delta	7	12-100	99% [84·5-99·9%]	Moderate
		Infection + 1st booster vaccine vs naive	Pfizer/BioNTech-Comirnaty,Moderna-mRNA-1273	Hospitalization or severe disease	Omicron	Alpha (B.1.1.7),Beta,Gamma,Delta	7	12-100	99·9% [95·3-98·9%]	Moderate
		Infection + 1st booster vaccine vs naive	Pfizer/BioNTech-Comirnaty,Moderna-mRNA-1273	Hospitalization or severe disease	Omicron	Alpha (B.1.1.7),Beta,Gamma,Delta	7	12-100	97% [94-99%]	Moderate
		Infection + full primary series vaccine vs naive	Pfizer/BioNTech-Comirnaty,Moderna-mRNA-1273	Any Infection	Omicron	Alpha (B.1.1.7),Beta,Gamma,Delta	7	12-100	68% [67-70%]	Serious
		Infection + full primary series vaccine vs naive	Pfizer/BioNTech-Comirnaty,Moderna-mRNA-1273	Any Infection	Omicron	Alpha (B.1.1.7),Beta,Gamma,Delta	34 [7-60]	12-100	82% [80-84%]	Serious
		Infection + full primary series vaccine vs naive	Pfizer/BioNTech-Comirnaty,Moderna-mRNA-1273	Any Infection	Omicron	Alpha (B.1.1.7),Beta,Gamma,Delta	120 [60-179]	12-100	67% [65-68%]	Serious

		Infection + full primary series vaccine vs naive	Pfizer/BioNTech-Comirnaty,Moderna-mRNA-1273	Any Infection	Omicron	Alpha (B.1.1.7),Beta,Gamma,Delta	224 [180-269]	12-100	63% [60-65%]	Serious
		Infection + full primary series vaccine vs naive	Pfizer/BioNTech-Comirnaty,Moderna-mRNA-1273	Any Infection	Omicron	Alpha (B.1.1.7),Beta,Gamma,Delta	314 [270-359]	12-100	62% [42-75%]	Serious
		Infection + full primary series vaccine vs naive	Pfizer/BioNTech-Comirnaty,Moderna-mRNA-1273	Hospitalization or severe disease	Omicron	Alpha (B.1.1.7),Beta,Gamma,Delta	7	12-100	99·6% [93·3-100%]	Moderate
		Infection + full primary series vaccine vs naive	Pfizer/BioNTech-Comirnaty,Moderna-mRNA-1273	Hospitalization or severe disease	Omicron	Alpha (B.1.1.7),Beta,Gamma,Delta	97	12-100	86·3% [1-99·1%]	Moderate
		Infection + full primary series vaccine vs naive	Pfizer/BioNTech-Comirnaty,Moderna-mRNA-1273	Hospitalization or severe disease	Omicron	Alpha (B.1.1.7),Beta,Gamma,Delta	97	12-100	99·8% [67·3-99·4%]	Moderate
		Infection + full primary series vaccine vs naive	Pfizer/BioNTech-Comirnaty,Moderna-mRNA-1273	Hospitalization or severe disease	Omicron	Alpha (B.1.1.7),Beta,Gamma,Delta	7	12-100	94% [91-96%]	Moderate
		Infection + full primary series vaccine vs naive	Pfizer/BioNTech-Comirnaty,Moderna-mRNA-1273	Hospitalization or severe disease	Omicron	Alpha (B.1.1.7),Beta,Gamma,Delta	7	12-100	99·9% [95·1-97·9%]	Moderate
		Infection + 1st booster vaccine vs infection	Pfizer/BioNTech-Comirnaty,Moderna-mRNA-1273	Any Infection	Omicron	Alpha (B.1.1.7),Beta,Gamma,Delta	7	12-100	69·4% [66·1-72·3%]	Serious
		Infection + 1st booster vaccine vs infection	Pfizer/BioNTech-Comirnaty,Moderna-mRNA-1273	Hospitalization or severe disease	Omicron	Alpha (B.1.1.7),Beta,Gamma,Delta	7	12-100	82·3% [55·5-92·9%]	Moderate
		Infection + full primary series vaccine vs infection	Pfizer/BioNTech-Comirnaty,Moderna-mRNA-1273	Any Infection	Omicron	Alpha (B.1.1.7),Beta,Gamma,Delta	7	12-100	29·1% [22·8-34·9%]	Serious
		Infection + full primary series vaccine vs infection	Pfizer/BioNTech-Comirnaty,Moderna-mRNA-1273	Hospitalization or severe disease	Omicron	Alpha (B.1.1.7),Beta,Gamma,Delta	7	12-100	74·6% [49·8-87·2%]	Moderate
		Infection + full primary series vaccine vs infection	Pfizer/BioNTech-Comirnaty,Moderna-mRNA-1273	Hospitalization or severe disease	Omicron	Alpha (B.1.1.7),Beta,Gamma,Delta	97	12-100	63·9% [1-95·3%]	Moderate
		Infection + 1st booster vaccine vs infection + full primary series vaccine	Pfizer/BioNTech-Comirnaty,Moderna-mRNA-1273	Any Infection	Omicron	Alpha (B.1.1.7),Beta,Gamma,Delta	7	12-100	56·8% [53·6-59·7%]	Serious

		Infection + 1st booster vaccine vs infection + full primary series vaccine	Pfizer/BioNTech-Comirnaty,Moderna-mRNA-1273	Hospitalization or severe disease	Omicron	Alpha (B.1.1.7),Beta,Gamma,Delta	7	12-100	30·1% [1-70%]	Moderate
Cerqueira-Silva (Brazil)(13)	Test-negative case-control (Matched cohorts. Adjusted for matched design, comorbidities, pregnancy, race, days elapsed between tests, hospital admission, and age)	Infection + 1st booster vaccine vs naive	AstraZeneca-Vaxzevria	Any Infection	Omicron	Mixed variant	70	18-100	50·8% [48·9-52·7%]	Moderate
		Infection + 1st booster vaccine vs naive	AstraZeneca-Vaxzevria	Any Infection	Omicron	Mixed variant	6 [0-13]	18-100	68·8% [67·5-70%]	Moderate
		Infection + 1st booster vaccine vs naive	AstraZeneca-Vaxzevria	Any Infection	Omicron	Mixed variant	42 [14-69]	18-100	72·1% [71·4-72·8%]	Moderate
		Infection + 1st booster vaccine vs naive	CoronaVac	Any Infection	Omicron	Mixed variant	6 [0-13]	18-100	66·9% [64·7-69%]	Moderate
		Infection + 1st booster vaccine vs naive	CoronaVac	Any Infection	Omicron	Mixed variant	42 [14-69]	18-100	73·4% [72·4-74·3%]	Moderate
		Infection + 1st booster vaccine vs naive	CoronaVac	Any Infection	Omicron	Mixed variant	70	18-100	54·6% [53·7-55·5%]	Moderate
		Infection + 1st booster vaccine vs naive	Janssen-Ad26.COV2.S	Any Infection	Omicron	Mixed variant	6 [0-13]	18-100	52·4% [46·9-57·4%]	Moderate
		Infection + 1st booster vaccine vs naive	Janssen-Ad26.COV2.S	Any Infection	Omicron	Mixed variant	42 [14-69]	18-100	44·8% [42·4-47·2%]	Moderate
		Infection + 1st booster vaccine vs naive	Pfizer/BioNTech-Comirnaty	Any Infection	Omicron	Mixed variant	6 [0-13]	18-100	68% [65·8-70·2%]	Moderate
		Infection + 1st booster vaccine vs naive	Pfizer/BioNTech-Comirnaty	Any Infection	Omicron	Mixed variant	42 [14-69]	18-100	68·2% [66·4-69·9%]	Moderate
		Infection + 1st booster vaccine vs naive	Pfizer/BioNTech-Comirnaty	Any Infection	Omicron	Mixed variant	70	18-100	58·2% [45·4-68·1%]	Moderate
		Infection + 1st booster vaccine vs naive	AstraZeneca-Vaxzevria	Hospitalization or severe disease	Omicron	Mixed variant	42 [14-69]	18-100	98·1% [97·7-98·5%]	Moderate
		Infection + 1st booster vaccine vs naive	AstraZeneca-Vaxzevria	Hospitalization or severe disease	Omicron	Mixed variant	70	18-100	97·2% [96·2-98%]	Moderate
		Infection + 1st booster vaccine vs naive	AstraZeneca-Vaxzevria	Hospitalization or severe disease	Omicron	Mixed variant	6 [0-13]	18-100	97·6% [96·2-98·5%]	Moderate
		Infection + 1st booster vaccine vs naive	CoronaVac	Hospitalization or severe disease	Omicron	Mixed variant	42 [14-69]	18-100	96·9% [96-97·6%]	Moderate

		Infection + 1st booster vaccine vs naive	CoronaVac	Hospitalization or severe disease	Omicron	Mixed variant	70	18-100	96·7% [96·2-97·1%]	Moderate
		Infection + 1st booster vaccine vs naive	CoronaVac	Hospitalization or severe disease	Omicron	Mixed variant	6 [0-13]	18-100	95% [89·9-97·6%]	Moderate
		Infection + 1st booster vaccine vs naive	Janssen-Ad26.COV2.S	Hospitalization or severe disease	Omicron	Mixed variant	6 [0-13]	18-100	93·3% [72·9-98·3%]	Moderate
		Infection + 1st booster vaccine vs naive	Janssen-Ad26.COV2.S	Hospitalization or severe disease	Omicron	Mixed variant	42 [14-69]	18-100	97·8% [94-99·2%]	Moderate
		Infection + 1st booster vaccine vs naive	Pfizer/BioNTech-Comirnaty	Hospitalization or severe disease	Omicron	Mixed variant	6 [0-13]	18-100	99·6% [93·3-100%]	Moderate
		Infection + 1st booster vaccine vs naive	Pfizer/BioNTech-Comirnaty	Hospitalization or severe disease	Omicron	Mixed variant	42 [14-69]	18-100	96·8% [94·1-98·2%]	Moderate
		Infection + full primary series vaccine vs naive	AstraZeneca-Vaxzevria	Any Infection	Omicron	Mixed variant	6 [0-13]	18-100	59·7% [49·7-67·7%]	Moderate
		Infection + full primary series vaccine vs naive	AstraZeneca-Vaxzevria	Any Infection	Omicron	Mixed variant	42 [14-69]	18-100	45·5% [42·6-48·3%]	Moderate
		Infection + full primary series vaccine vs naive	AstraZeneca-Vaxzevria	Any Infection	Omicron	Mixed variant	104 [70-139]	18-100	38·8% [37·7-39·8%]	Moderate
		Infection + full primary series vaccine vs naive	AstraZeneca-Vaxzevria	Any Infection	Omicron	Mixed variant	140	18-100	40·7% [39·6-41·7%]	Moderate
		Infection + full primary series vaccine vs naive	CoronaVac	Any Infection	Omicron	Mixed variant	6 [0-13]	18-100	53·3% [43·3-61·4%]	Moderate
		Infection + full primary series vaccine vs naive	CoronaVac	Any Infection	Omicron	Mixed variant	42 [14-69]	18-100	46% [42·6-49·2%]	Moderate
		Infection + full primary series vaccine vs naive	CoronaVac	Any Infection	Omicron	Mixed variant	104 [70-139]	18-100	31% [29·4-32·5%]	Moderate
		Infection + full primary series vaccine vs naive	CoronaVac	Any Infection	Omicron	Mixed variant	140	18-100	36·2% [34·9-37·4%]	Moderate
		Infection + full primary series vaccine vs naive	Janssen-Ad26.COV2.S	Any Infection	Omicron	Mixed variant	14	18-100	39·7% [37·5-41·8%]	Moderate
		Infection + full primary series vaccine vs naive	Pfizer/BioNTech-Comirnaty	Any Infection	Omicron	Mixed variant	42 [14-69]	18-100	63·6% [62·5-64·7%]	Moderate

		Infection + full primary series vaccine vs naive	Pfizer/BioNTech-Comirnaty	Any Infection	Omicron	Mixed variant	6 [0-13]	18-100	71·1% [66·8-74·8%]	Moderate
		Infection + full primary series vaccine vs naive	Pfizer/BioNTech-Comirnaty	Any Infection	Omicron	Mixed variant	104 [70-139]	18-100	50·2% [49·4-50·9%]	Moderate
		Infection + full primary series vaccine vs naive	Pfizer/BioNTech-Comirnaty	Any Infection	Omicron	Mixed variant	140	18-100	45·7% [43·7-47·7%]	Moderate
		Infection + full primary series vaccine vs naive	AstraZeneca-Vaxzevria	Hospitalization or severe disease	Omicron	Mixed variant	42 [14-69]	18-100	89·9% [81·9-94·3%]	Moderate
		Infection + full primary series vaccine vs naive	AstraZeneca-Vaxzevria	Hospitalization or severe disease	Omicron	Mixed variant	104 [70-139]	18-100	93·9% [92·8-94·9%]	Moderate
		Infection + full primary series vaccine vs naive	AstraZeneca-Vaxzevria	Hospitalization or severe disease	Omicron	Mixed variant	140	18-100	94·5% [93·8-95·1%]	Moderate
		Infection + full primary series vaccine vs naive	CoronaVac	Hospitalization or severe disease	Omicron	Mixed variant	104 [70-139]	18-100	88·7% [85·4-91·3%]	Moderate
		Infection + full primary series vaccine vs naive	CoronaVac	Hospitalization or severe disease	Omicron	Mixed variant	42 [14-69]	18-100	88·4% [77·9-93·9%]	Moderate
		Infection + full primary series vaccine vs naive	CoronaVac	Hospitalization or severe disease	Omicron	Mixed variant	140	18-100	90·7% [89·5-91·8%]	Moderate
		Infection + full primary series vaccine vs naive	Janssen-Ad26.COV2.S	Hospitalization or severe disease	Omicron	Mixed variant	14	18-100	91·2% [87·2-93·9%]	Moderate
		Infection + full primary series vaccine vs naive	Pfizer/BioNTech-Comirnaty	Hospitalization or severe disease	Omicron	Mixed variant	6 [0-13]	18-100	95·5% [67·6-99·4%]	Moderate
		Infection + full primary series vaccine vs naive	Pfizer/BioNTech-Comirnaty	Hospitalization or severe disease	Omicron	Mixed variant	42 [14-69]	18-100	92% [88-94·7%]	Moderate
		Infection + full primary series vaccine vs naive	Pfizer/BioNTech-Comirnaty	Hospitalization or severe disease	Omicron	Mixed variant	104 [70-139]	18-100	94·7% [93·4-95·7%]	Moderate
		Infection + full primary series vaccine vs naive	Pfizer/BioNTech-Comirnaty	Hospitalization or severe disease	Omicron	Mixed variant	140	18-100	94% [91·4-95·9%]	Moderate
		Infection + 1st booster vaccine vs infection	AstraZeneca-Vaxzevria	Any Infection	Omicron	Mixed variant	6 [0-13]	18-100	56% [53·8-58%]	Moderate
		Infection + 1st booster vaccine vs infection	AstraZeneca-Vaxzevria	Any Infection	Omicron	Mixed variant	70	18-100	32·6% [29·4-35·7%]	Moderate

		Infection + 1st booster vaccine vs infection	AstraZeneca-Vaxzevria	Any Infection	Omicron	Mixed variant	42 [14-69]	18-100	60·5% [59·1-61·9%]	Moderate
		Infection + 1st booster vaccine vs infection	CoronaVac	Any Infection	Omicron	Mixed variant	70	18-100	37·9% [35·8-40%]	Moderate
		Infection + 1st booster vaccine vs infection	CoronaVac	Any Infection	Omicron	Mixed variant	42 [14-69]	18-100	62·7% [61-64·3%]	Moderate
		Infection + 1st booster vaccine vs infection	CoronaVac	Any Infection	Omicron	Mixed variant	6 [0-13]	18-100	54·7% [51·4-57·8%]	Moderate
		Infection + 1st booster vaccine vs infection	Janssen-Ad26.COV2.S	Any Infection	Omicron	Mixed variant	6 [0-13]	18-100	34·1% [26·2-41·1%]	Moderate
		Infection + 1st booster vaccine vs infection	Janssen-Ad26.COV2.S	Any Infection	Omicron	Mixed variant	42 [14-69]	18-100	22·8% [18·8-26·6%]	Moderate
		Infection + 1st booster vaccine vs infection	Pfizer/BioNTech-Comirnaty	Any Infection	Omicron	Mixed variant	70	18-100	43·3% [25·8-56·6%]	Moderate
		Infection + 1st booster vaccine vs infection	Pfizer/BioNTech-Comirnaty	Any Infection	Omicron	Mixed variant	6 [0-13]	18-100	55·7% [52·3-58·9%]	Moderate
		Infection + 1st booster vaccine vs infection	Pfizer/BioNTech-Comirnaty	Any Infection	Omicron	Mixed variant	42 [14-69]	18-100	56·4% [53·7-59%]	Moderate
		Infection + 1st booster vaccine vs infection	AstraZeneca-Vaxzevria	Hospitalization or severe disease	Omicron	Mixed variant	42 [14-69]	18-100	84·5% [79·4-88·4%]	Moderate
		Infection + 1st booster vaccine vs infection	AstraZeneca-Vaxzevria	Hospitalization or severe disease	Omicron	Mixed variant	6 [0-13]	18-100	79·4% [66·8-87·3%]	Moderate
		Infection + 1st booster vaccine vs infection	AstraZeneca-Vaxzevria	Hospitalization or severe disease	Omicron	Mixed variant	70	18-100	81·2% [72·5-87·1%]	Moderate
		Infection + 1st booster vaccine vs infection	CoronaVac	Hospitalization or severe disease	Omicron	Mixed variant	70	18-100	75·7% [69·6-80·7%]	Moderate
		Infection + 1st booster vaccine vs infection	CoronaVac	Hospitalization or severe disease	Omicron	Mixed variant	42 [14-69]	18-100	76·6% [68·1-82·8%]	Moderate
		Infection + 1st booster vaccine vs infection	CoronaVac	Hospitalization or severe disease	Omicron	Mixed variant	6 [0-13]	18-100	67·9% [32·6-84·7%]	Moderate
		Infection + 1st booster vaccine vs infection	Janssen-Ad26.COV2.S	Hospitalization or severe disease	Omicron	Mixed variant	42 [14-69]	18-100	84% [56·6-94·1%]	Moderate

		Infection + 1st booster vaccine vs infection	Janssen-Ad26.COV2.S	Hospitalization or severe disease	Omicron	Mixed variant	6 [0-13]	18-100	55·2% [1-89%]	Moderate
		Infection + 1st booster vaccine vs infection	Pfizer/BioNTech-Comirnaty	Hospitalization or severe disease	Omicron	Mixed variant	6 [0-13]	18-100	97·8% [64·9-99·9%]	Moderate
		Infection + 1st booster vaccine vs infection	Pfizer/BioNTech-Comirnaty	Hospitalization or severe disease	Omicron	Mixed variant	42 [14-69]	18-100	75% [53·3-86·7%]	Moderate
		Infection + full primary series vaccine vs infection	AstraZeneca-Vaxzevria	Any Infection	Omicron	Mixed variant	6 [0-13]	18-100	43·6% [29·4-54·9%]	Moderate
		Infection + full primary series vaccine vs infection	AstraZeneca-Vaxzevria	Any Infection	Omicron	Mixed variant	104 [70-139]	18-100	14·5% [11·9-17·1%]	Moderate
		Infection + full primary series vaccine vs infection	AstraZeneca-Vaxzevria	Any Infection	Omicron	Mixed variant	42 [14-69]	18-100	25·5% [21-29·7%]	Moderate
		Infection + full primary series vaccine vs infection	AstraZeneca-Vaxzevria	Any Infection	Omicron	Mixed variant	140	18-100	17% [14·4-19·6%]	Moderate
		Infection + full primary series vaccine vs infection	CoronaVac	Any Infection	Omicron	Mixed variant	140	18-100	12·3% [9·4-15·1%]	Moderate
		Infection + full primary series vaccine vs infection	CoronaVac	Any Infection	Omicron	Mixed variant	42 [14-69]	18-100	23·4% [18·2-28·3%]	Moderate
		Infection + full primary series vaccine vs infection	CoronaVac	Any Infection	Omicron	Mixed variant	6 [0-13]	18-100	34·5% [20·4-46·1%]	Moderate
		Infection + full primary series vaccine vs infection	CoronaVac	Any Infection	Omicron	Mixed variant	104 [70-139]	18-100	7·3% [4-10·4%]	Moderate
		Infection + full primary series vaccine vs infection	Janssen-Ad26.COV2·S	Any Infection	Omicron	Mixed variant	14	18-100	16·2% [12·4-19·8%]	Moderate
		Infection + full primary series vaccine vs infection	Pfizer/BioNTech-Comirnaty	Any Infection	Omicron	Mixed variant	42 [14-69]	18-100	51·9% [50-53·8%]	Moderate
		Infection + full primary series vaccine vs infection	Pfizer/BioNTech-Comirnaty	Any Infection	Omicron	Mixed variant	6 [0-13]	18-100	60·3% [54·3-65·5%]	Moderate
		Infection + full primary series vaccine vs infection	Pfizer/BioNTech-Comirnaty	Any Infection	Omicron	Mixed variant	140	18-100	26·2% [22·8-29·4%]	Moderate
		Infection + full primary series vaccine vs infection	Pfizer/BioNTech-Comirnaty	Any Infection	Omicron	Mixed variant	104 [70-139]	18-100	32·8% [30·7-34·7%]	Moderate

		Infection + full primary series vaccine vs infection	AstraZeneca-Vaxzevria	Hospitalization or severe disease	Omicron	Mixed variant	42 [14-69]	18-100	41% [1-67·8%]	Moderate
		Infection + full primary series vaccine vs infection	AstraZeneca-Vaxzevria	Hospitalization or severe disease	Omicron	Mixed variant	140	18-100	55·4% [44·6-64·1%]	Moderate
		Infection + full primary series vaccine vs infection	AstraZeneca-Vaxzevria	Hospitalization or severe disease	Omicron	Mixed variant	104 [70-139]	18-100	57·1% [44·8-66·7%]	Moderate
		Infection + full primary series vaccine vs infection	CoronaVac	Hospitalization or severe disease	Omicron	Mixed variant	42 [14-69]	18-100	34·1% [1-66·3%]	Moderate
		Infection + full primary series vaccine vs infection	CoronaVac	Hospitalization or severe disease	Omicron	Mixed variant	140	18-100	34·4% [18·3-47·3%]	Moderate
		Infection + full primary series vaccine vs infection	CoronaVac	Hospitalization or severe disease	Omicron	Mixed variant	104 [70-139]	18-100	39·8% [16·9-56·4%]	Moderate
		Infection + full primary series vaccine vs infection	Janssen-Ad26.COV2.S	Hospitalization or severe disease	Omicron	Mixed variant	14	18-100	39·5% [8·3-60%]	Moderate
		Infection + full primary series vaccine vs infection	Pfizer/BioNTech-Comirnaty	Hospitalization or severe disease	Omicron	Mixed variant	140	18-100	53·6% [30·2-69·1%]	Moderate
		Infection + full primary series vaccine vs infection	Pfizer/BioNTech-Comirnaty	Hospitalization or severe disease	Omicron	Mixed variant	104 [70-139]	18-100	67·8% [57·4-75·6%]	Moderate
		Infection + full primary series vaccine vs infection	Pfizer/BioNTech-Comirnaty	Hospitalization or severe disease	Omicron	Mixed variant	42 [14-69]	18-100	59·6% [36·6-74·2%]	Moderate
		Infection + full primary series vaccine vs infection	Pfizer/BioNTech-Comirnaty	Hospitalization or severe disease	Omicron	Mixed variant	6 [0-13]	18-100	72·6% [1-96·2%]	Moderate
Chin (USA)(14)	Test-negative case-control (Matched based on test week, prison, position, Covid-19 risk score, and room type· Adjusted for age group, and sex)	Infection + 1st booster vaccine vs naive	Pfizer/BioNTech-Comirnaty,Moderna-mRNA-1273	Any Infection	Omicron	Delta (B.1.617.2)	68	18-100	87·4% [81·2-94%]	Moderate
		Infection + 1st booster vaccine vs naive	Pfizer/BioNTech-Comirnaty,Moderna-mRNA-1273	Any Infection	Omicron	Delta (B.1.617.2)	59	18-100	86% [77·2-95·9%]	Moderate
		Infection + full primary series vaccine vs naive	Pfizer/BioNTech-Comirnaty,Moderna-mRNA-1273	Any Infection	Omicron	Delta (B.1.617.2)	201	18-100	72·2% [68-80%]	Moderate
		Infection + full primary series vaccine vs naive	Pfizer/BioNTech-Comirnaty,Moderna-mRNA-1273	Any Infection	Omicron	Delta (B.1.617.2)	230	18-100	74·9% [66·1-86·7%]	Moderate
Lind (USA)(15)	Test-negative case-control (Adjusted	Infection + 1st booster vaccine vs infection	Pfizer/BioNTech-Comirnaty,Moderna-mRNA-1273	Any Infection	Omicron	Mixed variant	14	5-100	45·1% [19-62·8%]	Serious

	for date of test, age (continuous), sex, race/ethnicity, comorbidity score, clinical encounters, insurance group, and regional social vulnerability)	Infection + 1st booster vaccine vs infection	Pfizer/BioNTech-Comirnaty,Moderna-mRNA-1273	Any Infection	Omicron	Mixed variant	82 [14-149]	5-100	45·8% [20-63·2%]	Serious
		Infection + 1st booster vaccine vs infection	Pfizer/BioNTech-Comirnaty,Moderna-mRNA-1273	Any Infection	Omicron	Mixed variant	14	5-100	36% [1-76·2%]	Serious
		Infection + 1st booster vaccine vs infection	Pfizer/BioNTech-Comirnaty,Moderna-mRNA-1273	Any Infection	Omicron	Mixed variant	14	5-100	38·5% [7·2-59·3%]	Serious
		Infection + 1st booster vaccine vs infection	Pfizer/BioNTech-Comirnaty,Moderna-mRNA-1273	Any Infection	Omicron	Mixed variant	14	5-100	36·3% [1-76·4%]	Serious
		Infection + 1st booster vaccine vs infection	Pfizer/BioNTech-Comirnaty,Moderna-mRNA-1273	Any Infection	Omicron	Mixed variant	14	5-100	48·5% [22·2-65·9%]	Serious
		Infection + 1st booster vaccine vs infection	Pfizer/BioNTech-Comirnaty,Moderna-mRNA-1273	Any Infection	Omicron	Mixed variant	14	5-100	34·3% [1-75·7%]	Serious
		Infection + 1st booster vaccine vs infection	Pfizer/BioNTech-Comirnaty,Moderna-mRNA-1273	Any Infection	Omicron	Mixed variant	14	5-100	21·3% [1-71%]	Serious
		Infection + full primary series vaccine vs infection	Pfizer/BioNTech-Comirnaty,Moderna-mRNA-1273	Any Infection	Omicron	Mixed variant	82 [14-149]	5-100	37·3% [8·4-57·1%]	Serious
		Infection + full primary series vaccine vs infection	Pfizer/BioNTech-Comirnaty,Moderna-mRNA-1273	Any Infection	Omicron	Mixed variant	150	5-100	34·2% [18·7-46·8%]	Serious
		Infection + full primary series vaccine vs infection	Pfizer/BioNTech-Comirnaty,Moderna-mRNA-1273	Any Infection	Omicron	Mixed variant	82 [14-149]	5-100	38·4% [10·5-57·6%]	Serious
		Infection + full primary series vaccine vs infection	Pfizer/BioNTech-Comirnaty,Moderna-mRNA-1273	Any Infection	Omicron	Mixed variant	150	5-100	33·3% [17·6-45·9%]	Serious
		Infection + full primary series vaccine vs infection	Pfizer/BioNTech-Comirnaty,Moderna-mRNA-1273	Any Infection	Omicron	Mixed variant	82 [14-149]	5-100	30·8% [1-52·4%]	Serious
		Infection + full primary series vaccine vs infection	Pfizer/BioNTech-Comirnaty,Moderna-mRNA-1273	Any Infection	Omicron	Mixed variant	150	5-100	23·8% [6-38·2%]	Serious
		Infection + full primary series vaccine vs infection	Pfizer/BioNTech-Comirnaty,Moderna-mRNA-1273	Any Infection	Omicron	Mixed variant	14	5-100	33·2% [3·7-53·6%]	Serious
		Infection + full primary series vaccine vs infection	Pfizer/BioNTech-Comirnaty,Moderna-mRNA-1273	Any Infection	Omicron	Mixed variant	14	5-100	33·1% [3·6-53·6%]	Serious
		Infection + full primary series vaccine vs infection	Pfizer/BioNTech-Comirnaty,Moderna-mRNA-1273	Any Infection	Omicron	Mixed variant	14	5-100	32·4% [2·6-53%]	Serious

		Infection + full primary series vaccine vs infection	Pfizer/BioNTech-Comirnaty,Moderna-mRNA-1273	Any Infection	Omicron	Mixed variant	82 [14-149]	5-100	36·1% [7·1-56·1%]	Serious
		Infection + full primary series vaccine vs infection	Pfizer/BioNTech-Comirnaty,Moderna-mRNA-1273	Any Infection	Omicron	Mixed variant	150	5-100	34% [18·5-46·5%]	Serious
		Infection + full primary series vaccine vs infection	Pfizer/BioNTech-Comirnaty,Moderna-mRNA-1273	Any Infection	Omicron	Mixed variant	14	5-100	26·2% [1-48·6%]	Serious
		Infection + 1st booster vaccine vs infection + full primary series vaccine	Pfizer/BioNTech-Comirnaty,Moderna-mRNA-1273	Any Infection	Omicron	Mixed variant	328 [258-384]	5-100	17·0% [1-44·0%]	Serious
Medic (Serbia)(21)	Traditional case-control (Matched chorts· Adjusted for age and sex·)	Infection + 1st booster vaccine vs infection	Pfizer/BioNTech-Comirnaty,Moderna-mRNA-1273,AstraZeneca-Vaxzevria, Gamaleja-Sputnik-V, Gam-COVID-Vac,BBIBP-CorV	Any Infection	Omicron	Mixed variant	7	18-100	18·7% [12·3-24·8%]	Serious
		Infection + 1st booster vaccine vs infection + partial vaccine	Pfizer/BioNTech-Comirnaty,Moderna-mRNA-1273,AstraZeneca-Vaxzevria,Gamaleja-Sputnik-V,BBIBP-CorV	Any Infection	Omicron	Mixed variant	14	18-100	24·8% [7·4-39%]	Serious
		Infection + 1st booster vaccine vs infection + full primary series vaccine	Pfizer/BioNTech-Comirnaty,Moderna-mRNA-1273,AstraZeneca-Vaxzevria,Gamaleja-Sputnik-V,BBIBP-CorV	Any Infection	Omicron	Mixed variant	7	18-100	33·3% [27-38·7%]	Serious
Nielsen (Denmark)(22)	Retrospective cohort (Adjusted for age, sex, comorbidity, region of affiliation, staying at hospital, vaccination status, and time since vaccination)	Infection + full primary series vaccine vs infection	Pfizer/BioNTech-Comirnaty,Moderna-mRNA-1273,AstraZeneca-Vaxzevria,Janssen-Ad26.COV2·S	Any Infection	Omicron	Index-Delta	298 [284-313]	0-100	25·8% [1-46%]	Serious
		Infection + full primary series vaccine vs infection	Pfizer/BioNTech-Comirnaty,Moderna-mRNA-1273,AstraZeneca-Vaxzevria,Janssen-Ad26.COV2.S	Any Infection	Omicron	Index-Delta	328 [314-343]	0-100	24·8% [1-48%]	Serious
		Infection + full primary series vaccine vs infection	Pfizer/BioNTech-Comirnaty,Moderna-mRNA-1273,AstraZeneca-Vaxzevria,Janssen-Ad26.COV2.S	Any Infection	Omicron	Index-Delta	344	0-100	28·6% [1-52%]	Serious
		Infection + full primary series vaccine vs infection	Pfizer/BioNTech-Comirnaty,Moderna-mRNA-1273,AstraZeneca-Vaxzevria,Janssen-Ad26.COV2.S	Any Infection	Omicron	Index-Delta	208 [194-223]	0-100	18·4% [11·2-24·8%]	Serious

		Infection + full primary series vaccine vs infection	Pfizer/BioNTech-Comirnaty,Moderna-mRNA-1273,AstraZeneca-Vaxzevria,Janssen-Ad26.COV2.S	Any Infection	Omicron	Index-Delta	238 [224-253]	0-100	19·3% [6·9-31·4%]	Serious
		Infection + full primary series vaccine vs infection	Pfizer/BioNTech-Comirnaty,Moderna-mRNA-1273,AstraZeneca-Vaxzevria,Janssen-Ad26.COV2.S	Any Infection	Omicron	Index-Delta	268 [254-283]	0-100	37·1% [19·3-51%]	Serious
		Infection + full primary series vaccine vs infection	Pfizer/BioNTech-Comirnaty,Moderna-mRNA-1273,AstraZeneca-Vaxzevria,Janssen-Ad26.COV2.S	Any Infection	Omicron	Index-Delta	28 [14-43]	0-100	56·1% [54-58·2%]	Serious
		Infection + full primary series vaccine vs infection	Pfizer/BioNTech-Comirnaty,Moderna-mRNA-1273,AstraZeneca-Vaxzevria,Janssen-Ad26.COV2.S	Any Infection	Omicron	Index-Delta	88 [74-103]	0-100	39·1% [35·2-42%]	Serious
		Infection + full primary series vaccine vs infection	Pfizer/BioNTech-Comirnaty,Moderna-mRNA-1273,AstraZeneca-Vaxzevria,Janssen-Ad26.COV2.S	Any Infection	Omicron	Index-Delta	118 [104-133]	0-100	30·4% [28·6-33·3%]	Serious
		Infection + full primary series vaccine vs infection	Pfizer/BioNTech-Comirnaty,Moderna-mRNA-1273,AstraZeneca-Vaxzevria,Janssen-Ad26.COV2.S	Any Infection	Omicron	Index-Delta	148 [134-163]	0-100	17·5% [14·8-19·3%]	Serious
		Infection + full primary series vaccine vs infection	Pfizer/BioNTech-Comirnaty,Moderna-mRNA-1273,AstraZeneca-Vaxzevria,Janssen-Ad26.COV2.S	Any Infection	Omicron	Index-Delta	178 [164-193]	0-100	12·1% [8·6-14·8%]	Serious
		Infection + full primary series vaccine vs infection	Pfizer/BioNTech-Comirnaty,Moderna-mRNA-1273,AstraZeneca-Vaxzevria,Janssen-Ad26.COV2.S	Any Infection	Omicron	Index-Delta	58 [44-73]	0-100	46% [43-49%]	Serious
Plumb (USA)(23)	Test-negative case-control (Matched cohorts. Adjusted for sex, race/ethnicity, clinical encounters, underlying health conditions, and days since the previous infection)	Infection + 1st booster vaccine vs infection	Moderna-mRNA-1273,Pfizer/BioNTech-Comirnaty	Hospitalization or severe disease	Omicron	Mixed variant	14	18-100	61·6% [51·4-69·7%]	Moderate
		Infection + full primary series vaccine vs infection	Pfizer/BioNTech-Comirnaty,Moderna-mRNA-1273	Hospitalization or severe disease	Omicron	Mixed variant	14	18-100	40·3% [30·6-48·6%]	Moderate

Shrestha (USA)(24)	Retrospective cohort (Adjusted for boosting dose, time since SARS-CoV-2 exposure, time since prior infection, and vaccine doses)	Infection + 1st booster vaccine vs infection + partial primary series vaccine	Pfizer/BioNTech-Comirnaty,Moderna-mRNA-1273	Any Infection	Omicron	Index-Delta	7	0-100	1% [1-23%]	Serious
		Infection + 1st booster vaccine vs infection	Pfizer/BioNTech-Comirnaty,Moderna-mRNA-1273	Any Infection	Omicron	Index-Delta	331 [288-363]	0-11	59·0% [46·8-68·3%]	Serious
Šmíd (Czechia) (18)	Traditional case-control (Adjusted for age group, sex, and calendar time)	Infection + 1st booster vaccine vs naive	Pfizer/BioNTech-Comirnaty,AstraZeneca-Vaxzevria,Moderna-mRNA-1273,Janssen-Ad26.COV2.S	Any Infection	Omicron	Delta (B.1.617.2)	30 [0-60]	0-100	91·4% [88·2-93·6%]	Moderate
		Infection + 1st booster vaccine vs naive	Pfizer/BioNTech-Comirnaty,AstraZeneca-Vaxzevria,Moderna-mRNA-1273,Janssen-Ad26.COV2.S	Any Infection	Omicron	Delta (B.1.617.2)	61	0-100	80·8% [70·4-88·2%]	Moderate
		Infection + 1st booster vaccine vs naive	Pfizer/BioNTech-Comirnaty,AstraZeneca-Vaxzevria,Moderna-mRNA-1273,Janssen-Ad26.COV2.S	Any Infection	Omicron	Wild-type,Alpha (B.1.1.7)	30 [0-60]	0-100	72·5% [71·5-73·5%]	Moderate
		Infection + 1st booster vaccine vs naive	Pfizer/BioNTech-Comirnaty,AstraZeneca-Vaxzevria,Moderna-mRNA-1273,Janssen-Ad26.COV2.S	Any Infection	Omicron	Wild-type,Alpha (B.1.1.7)	61	0-100	46·1% [43·1-50·1%]	Moderate
		Infection + 1st booster vaccine vs naive	Pfizer/BioNTech-Comirnaty,AstraZeneca-Vaxzevria,Moderna-mRNA-1273,Janssen-Ad26.COV2.S	Hospitalization or severe disease	Omicron	Wild-type,Alpha (B.1.1.7)	30 [0-60]	0-100	95% [78-99%]	Moderate
		Infection + 1st booster vaccine vs naive	Pfizer/BioNTech-Comirnaty,AstraZeneca-Vaxzevria,Moderna-mRNA-1273,Janssen-Ad26.COV2.S	Hospitalization or severe disease	Omicron	Wild-type,Alpha (B.1.1.7)	61	0-100	90% [64-98%]	Moderate
		Infection + 1st booster vaccine vs naive	Pfizer/BioNTech-Comirnaty,AstraZeneca-Vaxzevria,Moderna-mRNA-1273,Janssen-Ad26.COV2.S	Hospitalization or severe disease	Omicron	Delta (B.1.617.2)	61	0-100	71% [1-96%]	Moderate
		Infection + full primary series vaccine vs naive	Pfizer/BioNTech-Comirnaty,AstraZeneca-	Any Infection	Omicron	Delta (B.1.617.2)	30 [0-60]	0-100	80·8% [73·5-86·1%]	Moderate

			Vaxzevria,Moderna-mRNA-1273,Janssen-Ad26.COV2.S							
		Infection + full primary series vaccine vs naive	Pfizer/BioNTech-Comirnaty,AstraZeneca-Vaxzevria,Moderna-mRNA-1273,Janssen-Ad26.COV2.S	Any Infection	Omicron	Delta (B.1.617.2)	61	0-100	85·1% [84-87·2%]	Moderate
		Infection + full primary series vaccine vs naive	Pfizer/BioNTech-Comirnaty,AstraZeneca-Vaxzevria,Moderna-mRNA-1273,Janssen-Ad26.COV2.S	Any Infection	Omicron	Wild-type,Alpha (B.1.1.7)	30 [0-60]	0-100	75·6% [74·6-76·7%]	Moderate
		Infection + full primary series vaccine vs naive	Pfizer/BioNTech-Comirnaty,AstraZeneca-Vaxzevria,Moderna-mRNA-1273,Janssen-Ad26.COV2.S	Any Infection	Omicron	Wild-type,Alpha (B.1.1.7)	61	0-100	43·1% [42·1-44·1%]	Moderate
		Infection + full primary series vaccine vs naive	Pfizer/BioNTech-Comirnaty,AstraZeneca-Vaxzevria,Moderna-mRNA-1273,Janssen-Ad26.COV2.S	Hospitalization or severe disease	Omicron	Delta (B.1.617.2)	61	0-100	93% [49-99%]	Moderate
		Infection + full primary series vaccine vs naive	Pfizer/BioNTech-Comirnaty,AstraZeneca-Vaxzevria,Moderna-mRNA-1273,Janssen-Ad26.COV2.S	Hospitalization or severe disease	Omicron	Wild-type,Alpha (B.1.1.7)	30 [0-60]	0-100	94% [77-95%]	Moderate
		Infection + full primary series vaccine vs naive	Pfizer/BioNTech-Comirnaty,AstraZeneca-Vaxzevria,Moderna-mRNA-1273,Janssen-Ad26.COV2.S	Hospitalization or severe disease	Omicron	Wild-type,Alpha (B.1.1.7)	61	0-100	73% [78-99%]	Moderate

Appendix 7. Risk of bias assessments

Of the 97 estimates reporting the protective effectiveness of prior infection, 27 (27·8%) were at moderate risk of bias and 70 (72·2%) were at serious risk of bias (Appendix, pp 38-39).

a) Studies on the protective effectiveness of prior infection (prior infection vs. immune naive)

First author (Country)	Estimates in analysis (n)	Variant	Outcome	Bias due to confounding	Bias due to participant selection	Bias due to intervention classification	Bias due to deviations from intended interventions	Bias due to missing data	Bias in measurement of outcomes	Bias due to selection of reported result	Overall Risk of bias
Altarawneh 1 (Qatar)[9]	3	Omicron	Any infection	Moderate	Low	Low	Low	Serious	Low	Moderate	Serious
	1	Omicron	Hospitalization or severe disease	Moderate	Low	Low	Low	Moderate	Moderate	Moderate	Moderate
Altarawneh 2 (Qatar)[10]	1	Omicron	Any infection (Symptomatic)	Moderate	Low	Low	Low	Moderate	Moderate	Moderate	Moderate
	1	Omicron	Hospitalization or severe disease	Moderate	Low	Low	Low	Moderate	Low	Moderate	Moderate
	1	Omicron (BA.1)	Any infection (Symptomatic)	Moderate	Low	Low	Low	Moderate	Moderate	Moderate	Moderate
	1	Omicron (BA.1)	Hospitalization or severe disease	Moderate	Low	Low	Low	Moderate	Low	Moderate	Moderate
	1	Omicron (BA.2)	Any infection (Symptomatic)	Moderate	Low	Low	Low	Moderate	Moderate	Moderate	Moderate
	1	Omicron (BA.2)	Hospitalization or severe disease	Moderate	Low	Low	Low	Moderate	Low	Moderate	Moderate
Andeweg (Netherland)[11]	10	Omicron (BA.1)	Any infection	Moderate	Serious	Moderate	Low	Low	Low	Moderate	Serious
	10	Omicron (BA.1)	Any infection (Symptomatic)	Moderate	Serious	Moderate	Low	Low	Low	Moderate	Serious
	5	Omicron (BA.2)	Any infection	Moderate	Serious	Moderate	Low	Low	Low	Moderate	Serious

	5	Omicron (BA.2)	Any infection (Symptomatic)	Moderate	Serious	Moderate	Low	Low	Low	Moderate	Serious
Carazo (Canada)[12]	7	Omicron	Any infection	Moderate	Serious	Low	Low	Low	Low	Moderate	Serious
	2	Omicron	Any infection (Symptomatic)	Moderate	Low	Low	Low	Serious	Low	Moderate	Serious
	2	Omicron	Hospitalization or severe disease	Moderate	Low	Low	Low	Low	Low	Moderate	Moderate
Cerqueira-Silva (Brazil)[13]	3	Omicron	Any infection (Symptomatic)	Low	Low	Low	Low	Low	Low	Moderate	Moderate
	3	Omicron	Hospitalization	Moderate	Serious	Low	Low	Moderate	Moderate	Moderate	Serious
Chin (USA)[14]	2	Omicron	Any infection	Low	Low	Low	Low	Low	Low	Moderate	Moderate
Lind (USA)[15]	1	Omicron	Any infection	Moderate	Low	Low	Low	Moderate	Moderate	Moderate	Moderate
Michlmayr (Denmark)[16]	4	Omicron	Any infection	Moderate	Moderate	Low	Low	Low	Low	Serious	Serious
	4	Omicron	Any infection (Symptomatic)	Moderate	Moderate	Low	Low	Low	Serious	Serious	Serious
	1	Omicron	Hospitalization or severe disease	Moderate	Moderate	Low	Low	Low	Low	Serious	Serious
Nyberg (UK)[17]	8	Omicron	Hospitalization or severe disease	Serious	Moderate	Serious	Low	Serious	Low	Moderate	Serious
	8	Omicron	Severe disease	Serious	Moderate	Serious	Low	Serious	Low	Moderate	Serious
Šmíd (Czech)[18]	6	Omicron	Any infection	Moderate	Low	Low	Low	Moderate	Low	Moderate	Moderate
	6	Omicron	Hospitalization or severe disease	Moderate	Low	Low	Low	Moderate	Low	Moderate	Moderate

Of the 153 estimates reporting the protective effectiveness of hybrid immunity compared to immune-naive individuals, 78 (51·0%) were at moderate risk of bias and 75 (49·0%) were at serious risk of bias (Appendix, pp 40-42).

Of the 86 estimates reporting the comparative protective effectiveness of hybrid immunity relative to individuals with prior infection only, of which five reported on hybrid immunity with primary series vaccination, 48 (55·8%) were at moderate risk of bias and 38 (44.2%) were at serious risk of bias.

Of the six estimates reporting the comparative protective effectiveness of hybrid immunity with more vaccine doses relative to individuals with hybrid immunity with fewer vaccine doses, one (16·6%) was at moderate risk of bias and five (83·3%) were at serious risk of bias (Appendix, pp 40-42).

b) Studies on hybrid immunity

First author (country)	Exposure vs. Comparator	Estimates in analysis (n)	Variant	Outcome	Bias due to confounding	Bias due to participant selection	Bias due to intervention classification	Bias due to deviations from intended interventions	Bias due to missing data	Bias in measurement of outcomes	Bias due to selection of reported result	Overall risk of bias
Altarawneh 2 (Qatar)[10]	Infection and vaccination vs. naive	7	Omicron (BA.1, BA.2)	Any infection (symptomatic)	Moderate	Low	Low	Low	Low	Low	Moderate	Moderate
Andeweg (Netherland)[11]	Infection and vaccination vs. naive	21	Omicron (BA.1)	Any infection	Moderate	Serious	Moderate	Low	Low	Low	Moderate	Serious
	Infection and vaccination vs. naive	21	Omicron (BA.1)	Any infection (symptomatic)	Moderate	Serious	Moderate	Low	Low	Low	Moderate	Serious
	Infection and vaccination vs. naive	11	Omicron (BA.2)	Any infection	Moderate	Serious	Moderate	Low	Low	Low	Moderate	Serious
	Infection and vaccination vs. naive	11	Omicron (BA.2)	Any infection (symptomatic)	Moderate	Serious	Moderate	Low	Low	Low	Moderate	Serious
Björk (Sweden)[19]	Infection and vaccination vs. naive	3	Omicron	Hospitalization or severe disease	Moderate	Low	Low	Low	Low	Low	Serious	Serious
Bruel (France)[20]	Infection and vaccination vs. vaccination	1	Omicron	Any infection	Serious	Low	Low	Low	Moderate	Low	Moderate	Serious

Carazo (Canada)[12]	Infection and vaccination vs. naive [a]	8	Omicron	Any infection	Moderate	Serious	Low	Low	Serious	Low	Moderate	Serious
	Infection and vaccination vs. infection	2	Omicron	Any infection	Moderate	Serious	Low	Low	Serious	Low	Moderate	Serious
	Infection and vaccination vs. infection and vaccination	1	Omicron	Any infection	Moderate	Serious	Low	Low	Serious	Low	Moderate	Serious
	Infection and vaccination vs. naive	8	Omicron	Hospitalization or severe disease	Moderate	Low	Low	Low	Low	Low	Moderate	Moderate
	Infection and vaccination vs. infection	3	Omicron	Hospitalization or severe disease	Moderate	Low	Low	Low	Low	Low	Moderate	Moderate
	Infection and vaccination vs. infection and vaccination	1	Omicron	Hospitalization or severe disease	Moderate	Low	Low	Low	Low	Low	Moderate	Moderate
Cerqueira-Silva (Brazil)[13]	Infection and vaccination vs. naive	24	Omicron	Any infection (symptomatic)	Low	Moderate	Low	Low	Low	Low	Moderate	Moderate
	Infection and vaccination vs. infection	24	Omicron	Any infection (symptomatic)	Low	Moderate	Low	Low	Low	Low	Moderate	Moderate
	Infection and vaccination vs. naive	21	Omicron	Hospitalization or severe disease	Low	Low	Low	Low	Low	Moderate	Moderate	Moderate
	Infection and vaccination vs. infection	21	Omicron	Hospitalization or severe disease	Low	Low	Low	Low	Low	Moderate	Moderate	Moderate
Chin (USA)[14]	Infection and vaccination vs. naive	4	Omicron	Any infection	Low	Low	Low	Low	Low	Low	Moderate	Moderate

Lind (USA)[15]	Infection and vaccination vs. infection	20	Omicron	Any infection	Moderate	Low	Low	Low	Moderate	Serious	Moderate	Serious
	Infection and vaccination vs. vaccination	1	Omicron	Any infection	Moderate	Low	Low	Low	Moderate	Serious	Moderate	Serious
Medić (Serbia)[21]	Infection and vaccination vs. infection	1	Omicron	Any infection	Serious	Moderate	Low	Low	Low	Moderate	Moderate	Serious
	Infection and vaccination vs. infection and vaccination	2	Omicron	Any infection	Serious	Moderate	Low	Low	Low	Moderate	Moderate	Serious
Nielsen (Denmark)[22]	Infection and vaccination vs. infection	12	Omicron	Any infection	Moderate	Moderate	Low	Low	Low	Serious	Moderate	Serious
Plumb (USA)[23]	Infection and vaccination vs. infection	2	Omicron	Hospitalization	Moderate	Low	Moderate	Low	Low	Low	Moderate	Moderate
Shrestha (USA)[24]	Infection and vaccination vs. infection and vaccination	1	Omicron	Any infection	Serious	Moderate	Serious	Low	Low	Serious	Moderate	Serious
	Infection and vaccination vs. infection	1	Omicron	Any infection	Serious	Moderate	Serious	Low	Low	Serious	Moderate	Serious
Šmíd (Czech)[18]	Infection and vaccination vs. naive	8	Omicron	Any infection	Moderate	Low	Low	Low	Moderate	Low	Moderate	Moderate
	Infection and vaccination vs. naive	6	Omicron	Hospitalization	Moderate	Low	Low	Low	Moderate	Low	Moderate	Moderate

Appendix 8. Summary of results for studies reporting sub-group data by age

Three prior infection effectiveness studies reported reinfection results by age subgroups. The reported estimates of protection did not differ between age groups.

Three hybrid immunity effectiveness studies reported reinfection results by age subgroups. One study reported that children aged 12-17 and older adults aged 50-69 had more protection from hybrid immunity effectiveness with partial primary series and full primary series than adults aged 18-49. The other reported estimates of protection did not differ between age groups.

Study	Age group	Exposure and comparator	Time since last immunological hit	Vaccine	Protection against any infection [95% CI]
Carazo (Canada)[12]	12-17	Prior infection vs naive	90-730 days	N/A	57% [36-71%]
Carazo (Canada)	18-49	Prior infection vs naïve	90-730 days	N/A	44% [29-43%]
Carazo (Canada)	50-69	Prior infection vs naïve	90-730 days	N/A	51% [38-60%]
Carazo (Canada)	70+	Prior infection vs naïve	90-730 days	N/A	46% [16-65%]
Carazo (Canada)	12-17	Infection + partial primary series vaccine vs naïve	21 days	Mixed	78% [70-83%]
Carazo (Canada)	18-49	Infection + partial primary series vaccine vs naïve	21 days	Mixed	62% [60-65%]
Carazo (Canada)	50-69	Infection + partial primary series vaccine vs naïve	21 days	Mixed	71% [66-75%]
Carazo (Canada)	70+	Infection + partial primary series vaccine vs naïve	21 days	Mixed	79% [65-87%]
Carazo (Canada)	12-17	Infection + primary series vs naïve	7 days	Mixed	79% [74-93%]
Carazo (Canada)	18-49	Infection + primary series vs naïve	7 days	Mixed	67% [65-68%]
Carazo (Canada)	50-69	Infection + primary series vs naïve	7 days	Mixed	72% [69-74%]
Carazo (Canada)	70+	Infection + primary series vs naïve	7 days	Mixed	67% [60-73%]
Carazo (Canada)	12-17	Infection + 1st booster vaccine vs naïve	7 days	Mixed	96% [65-99%]
Carazo (Canada)	18-49	Infection + 1st booster vaccine vs naïve	7 days	Mixed	79% [77-81%]
Carazo (Canada)	50-69	Infection + 1st booster vaccine vs naïve	7 days	Mixed	86% [83-88%]
Carazo (Canada)	70+	Infection + 1st booster vaccine vs naïve	7 days	Mixed	81% [75-86%]
Andeweg (Netherlands) (Jan to Mar 2022 Cohort, BA.1 infections)[11]	0-11	Prior infection vs naïve	180 days	N/A	41% [34-48%]

Andeweg (Netherlands) (Jan to Mar 2022 Cohort, BA.1 infections)	12-17	Prior infection vs naïve	180 days	N/A	39% [30-46%]
Andeweg (Netherlands) (Jan to Mar 2022 Cohort, BA.1 infections)	18-29	Prior infection vs naive	180 days	N/A	37% [32-42%]
Andeweg (Netherlands) (Jan to Mar 2022 Cohort, BA.1 infections)	30-59	Prior infection vs naïve	180 days	N/A	35% [31-40%]
Andeweg (Netherlands) (Jan to Mar 2022 Cohort, BA.1 infections)	60+	Prior infection vs naïve	180 days	N/A	45% [30-57%]
Andeweg (Netherlands) (Jan to Mar 2022 Cohort, BA.1 infections)	12-17	Infection + primary series vs naïve	180 days	Mixed	69% [44-83%]
Andeweg (Netherlands) (Jan to Mar 2022 Cohort, BA.1 infections)	18-29	Infection + primary series vs naïve	180 days	Mixed	65% [57-72%]
Andeweg (Netherlands) (Jan to Mar 2022 Cohort, BA.1 infections)	30-59	Infection + primary series vs naïve	180 days	Mixed	58% [51-65%]
Andeweg (Netherlands) (Jan to Mar 2022 Cohort, BA.1 infections)	60+	Infection + primary series vs naive	180 days	Mixed	71% [48-83%]
Andeweg (Netherlands) (Jan to Mar 2022 Cohort, BA.2 infections)	0-11	Prior infection vs naïve	180 days	N/A	35% [23-46%]
Andeweg (Netherlands) (Jan to Mar 2022 Cohort, BA.2 infections)	12-17	Prior infection vs naïve	180 days	N/A	54% [43-63%]
Andeweg (Netherlands) (Jan to Mar 2022 Cohort, BA.2 infections)	18-29	Prior infection vs naïve	180 days	N/A	43% [37-48%]
Andeweg (Netherlands) (Jan to Mar 2022 Cohort, BA.2 infections)	30-59	Prior infection vs naïve	180 days	N/A	37% [30-42%]
Andeweg (Netherlands) (Jan to Mar 2022 Cohort, BA.2 infections)	60+	Prior infection vs naive	180 days	N/A	45% [24-61%]
Andeweg (Netherlands) (Jan to Mar 2022 Cohort, BA.2 infections)	12-17	Infection + primary series vs naïve	180 days	Mixed	81% [56-92%]
Andeweg (Netherlands) (Jan to Mar 2022 Cohort, BA.2 infections)	18-29	Infection + primary series vs naïve	180 days	Mixed	67% [57-75%]
Andeweg (Netherlands) (Jan to Mar 2022 Cohort, BA.2 infections)	30-59	Infection + primary series vs naïve	180 days	Mixed	62% [52-69%]
Andeweg (Netherlands) (Jan to Mar 2022 Cohort, BA.2 infections)	60+	Infection + primary series vs naïve	180 days	Mixed	36% [1-64%]
Andeweg (Netherlands) (Nov 2021 to Jan 2022 Cohort)[11]	0-11	Prior infection vs naïve	180 days	N/A	42% [16-60%]
Andeweg (Netherlands) (Nov 2021 to Jan 2022 Cohort)	12-17	Prior infection vs naive	180 days	N/A	37% [6-58%]
Andeweg (Netherlands) (Nov 2021 to Jan 2022 Cohort)	18-29	Prior infection vs naïve	180 days	N/A	7% [-8-20%]
Andeweg (Netherlands) (Nov 2021 to Jan 2022 Cohort)	30-59	Prior infection vs naïve	180 days	N/A	5% [-13-20%]
Andeweg (Netherlands) (Nov 2021 to Jan 2022 Cohort)	60+	Prior infection vs naive	180 days	N/A	20% [-49-57%]
Andeweg (Netherlands) (Nov 2021 to Jan 2022 Cohort)	12-17	Infection + primary series vs naïve	180 days	Mixed	50% [1-94%]
Andeweg (Netherlands) (Nov 2021 to Jan 2022 Cohort)	18-29	Infection + primary series vs naïve	180 days	Mixed	54% [35-67%]
Andeweg (Netherlands) (Nov 2021 to Jan 2022 Cohort)	30-59	Infection + primary series vs naïve	180 days	Mixed	50% [31-64%]
Andeweg (Netherlands) (Nov 2021 to Jan 2022 Cohort)	60+	Infection + primary series vs naive	180 days	Mixed	69% [27-87%]

Appendix 9. Sub-group analysis of the protective effectiveness of hybrid immunity by vaccine type

Subgroup meta-regression analysis showed differences in the level of protection and patterns of waning protection over time by vaccine type. Hybrid immunity effectiveness against hospitalization or severe disease was initially high (>88%) across all vaccine types at 3 months after the last immunological challenge. At 6 months the available data showed a maintenance of protection for mRNA vaccines (98·1% [91·2-99·6%]) but a greater reduction in protection for a mixture of vaccines (75·5% [13·7-98·4%]). In these studies, mixtures of vaccines could refer to single individuals receiving multiple different vaccine products or a group of individuals who collectively received multiple types of vaccine. Hybrid immunity effectiveness against reinfection was highest with a primary series of mRNA vaccines (60·9% [48·3-72·2%], n=5 studies), followed by mRNA and non-replicating viral vector (60·1% [43·0-75·1%], n=3 studies), non-replicating viral vectors vaccines (40·7% [39·6-41·7%], n=1 study) and inactivated vaccines (36·2% [34·9-37·4%], n=1 study). There were significant monthly reductions in reinfection from 3 to 6 months for mRNA vaccines but no change for mixtures of vaccines, with data being limited for other vaccine types.

Vaccine type	Exposure	Comparator	Severity	No. studies	No. estimates	Month 3	Month 6	Percentage point change in protection from 3 to 6 months [95% CI]
mRNA[a]	Prior infection + primary series	Naïve	Hospitalization or severe disease	3	11	97·7% [89·5-99·5%]	98·1% [91·2-99·6%]	-0·47 [-1·4 to +2·0]
NRVV[b]	Prior infection + primary series	Naïve	Hospitalization or severe disease	1	1	93·9% [92·8-94·9%][e]	94·5% [93·8-95·1%][f]	-
Inactivated[c]	Prior infection + primary series	Naïve	Hospitalization or severe disease	1	1	88·4% [77·9-93·9%][e]	90·7% [89·5-91·8%][f]	-
Mixed (NRVV + mRNA)[d]	Prior infection + primary series	Naïve	Hospitalization or severe disease	2	5	89·2% [73·9-96·0%]	75·5% [13·7-98·4%]	-13·6 [-67·0 to -3·8]
mRNA[a]	Prior infection + primary series	Naïve	Any Infection	5	13	68·8% [56·9-78·6%]	60·9% [48·3-72·2%]	-7·9 [-2·0 to -15·7]
NRVV[b]	Prior infection + primary series	Naïve	Any Infection	1	1	38·8% [37·7-39·8%][e]	40·7% [39·6-41·7%][f]	-
Inactivated[c]	Prior infection + primary series	Naïve	Any Infection	1	1	31·0% [29·4-32·5%][e]	36·2% [34·9-37·4%][f]	-
Mixed (NRVV + mRNA)[d]	Prior infection + primary series	Naïve	Any Infection	3	33	70·8% [55·1-82·8%]	60·1% [43·0-75·1%]	-10·7 [-4·5 to +24·8]

[a]mRNA vaccine type contain Pfizer and Moderna; [b]NRVV (Non-replicating viral vector) vaccine type contain AstraZeneca; [c]Inactivated type of vaccine refers to CoronaVac; [d]Mixed vaccine type refers to Pfizer, Moderna, AstraZeneca, and Johnson & Johnson (i.e., mRNA and NRVV). [e]Single estimate at week 10-19 from Cerqueira-Silva (Brazil). [f]Single estimate at over 20 weeks from Cerqueira-Silva (Brazil).

Appendix 10. Sub-group analysis of the protective effectiveness of prior infection and hybrid immunity by prior infection variant

Subgroup meta-regression analysis of the protective effectiveness of prior infection and hybrid immunity by variant causing the index infection was limited to protection from Alpha, Delta, and mixed pre-omicron variants, including Alpha and Delta. Data at 6 months showed no differences in the level of protection by the variant causing the index infection.

Prior infection variant	Exposure	Comparator	Severity	Number of studies	Number of estimates	Month 3	Month 6
**Prior infection**							
Alpha	Infection	Naïve	Hospitalization or severe disease	1	1	-	66·0% [54·0-75·0%][c]
Delta	Infection	Naïve	Hospitalization or severe disease	2	4	74·7% [60·6-85·1%]	-
Mixed variant	Infection	Naïve	Hospitalization or severe disease	5	8	77·7% [62·5-87·9%]	77·4% [66·6-85·5%]
Alpha	Infection	Naïve	Any Infection	1	4	64·2% [60·3-67·9%]	52·8% [50·1-55·5%]
Delta	Infection	Naïve	Any Infection	3	5	65·5% [29·5-89·6%]	55·3% [22·0-84·4%]
Mixed variant	Infection	Naïve	Any Infection	8	54	60·8% [44·6-75·0%]	47·4% [31·9-63·5%]
**Hybrid immunity**							
Alpha	Infection + primary series	Naïve	Hospitalization or severe disease	1	1	94·0% [77·0-95·0%][a]	-
Delta	Infection + primary series	Naïve	Hospitalization or severe disease	1	1	93·0% [49·0-99·0%][b]	-
Mixed variant	Infection + primary series	Naïve	Hospitalization or severe disease	4	21	96·4% [86·9-99·1%]	96·8% [88·3-99·2%]
Alpha	Infection + primary series	Naïve	Any Infection	1	1	75·6% [74·6-76·7%][a]	-
Delta	Infection + primary series	Naïve	Any Infection	5	19	79·6% [61·8-90·4%]	62·4% [40·6-80·1%]
Mixed variant	Infection + primary series	Naïve	Any Infection	5	35	63·7% [53·9-72·5%]	54·8% [44·6-64·5%]

[a]Single estimate at month 1 from Šmíd 2 (Czechia). [b]Single estimate at month 2 from Šmíd 2 (Czechia).[c]Single estimate at month 10 from Šmíd 1 (Czech).

Appendix 11. Sensitivity analysis of the protection conferred by prior infection and hybrid immunity compared to immune naïve for studies at different risk of bias

**Severity of Infection**	**No. studies**	**No. estimates**	**Month 1[a]**	**Month 2[b]**	**Month 3**	**Month 4**	**Month 6**	**Month 9**	**Month 12**	**Month 15**	**Percentage point change in protection from 3 to 6 months [95% CI][c]**	**Percentage point change in protection from 3 to 12 months [95% CI][c]**
**Prior Infection**												
Hospitalization or severe disease **All studies**	6	16	NA	83·2% [72·1-90·5%]	82·5% [71·8-89·7%]	81·7% [71·4-88·9%]	80·1% [70·3-87·2%]	77·5% [67·5-85·1%]	74·6% [63·1-83·5%]	71·6% [57·1-82·6%]	-2·4 [-5·1 to +4·7]	-7·8 [-20·9 to +12·1]
Hospitalization or severe disease **Moderate RoB only**	4	13	NA	77·9% [63·0-87·9%]	77·5% [64·2-86·9%]	77·1% [65·3-85·8%]	76·4% [67·1-83·7%]	77·3% [68·4-81·1%]	74·1% [66·4-80·6%]	72·9% [61·4-82·0%]	-	-
Any Infection[d] **All studies**	10	64	NA	69·5% [57·6-79·2%]	65·2% [52·9-75·9%]	60·7% [48-72·1%]	51·2% [38·6-63·7%]	37·0% [26·0-49·6%]	24·7% [16·4-35·5%]	15·5% [9·9-23·6%]	-14·0 [-12·0 to -18·2]	-40·5 [-33·9 to -51·9]
Any Infection[d] **Moderate RoB only**	6	17	NA	69·2% [58·0-78·6%]	65·1% [53·4-75·3%]	60·7% [48-7-71·6%]	51·5% [39·5-63·4%]	37·7% [27-1-49·6%]	25·6% [17·4-35·9%]	16·4% [10·7-24·2%]	-	-
**Hybrid Immunity - Primary Series**												
Hospitalization or severe disease **All studies**	5	23	95·7% [88·0-98·5%]	95·9% [88·5-98·6%]	96·0% [89·0-98·6%]	96·2% [89·4-98·7%]	96·5% [90·2-98·8%]	97·0% [90·9-99%]	97·4% [91·4-99·2%][e]	NA	+0·50 [-2·2 to +2·1]	+1·3 [-4·3 to +7·4]
Hospitalization or severe disease **Moderate RoB only**	4	20	96·6% [89·3-99·0%]	96·8% [89·8-99·0%]	96·9% [90·3-99·1%]	97·1% [90·7-99·1%]	97·3% [91·3-99·2%]	97·7% [92·1-99·3%]	98·0% [92·5-99·5%][e]	NA	-	-
Any Infection **All studies**	7	55	74·1% [64·8-81·6%]	71·6% [61·9-79·6%]	69·0% [58·9-77·5%]	66·2% [55·8-75·3%]	60·4% [49·6-70·3%]	51·1% [40·2-61·9%]	41·8% [31·5-52·8%][e]	NA	-8·6 [-1·7 to -17·2]	-27·2 [-6·4 to -53·2]
Any Infection **Moderate RoB only**	4	20	77·8% [62·7-88·0%]	74·7% [58·6-86·0%]	71·3% [54·4-83·8%]	67·7% [50·1-81·3%]	59·7% [41·6-75·5%]	46·9% [29·8-64·9%]	34·5% [20·1-52·5%][e]	NA	-	-
**Hybrid Immunity – First Booster**												
Hospitalization or severe disease **All studies**	4	17	98·0% [92·9-99·5%]	97·6% [91·6-99·4%]	97·2% [90·0-99·3%]	96·7% [87·9-99·1%]	95·3% [81·9-98·9%][e]	NA	NA	NA	-1·8 [-10·3 to +0·77]	NA
Hospitalization or severe disease **Moderate RoB only**	4	17	98·0% [92·9-99·5%]	97·6% [91·6-99·4%]	97·2% [90·0-99·3%]	96·7% [87·9-99·1%]	95·3% [81·9-98·9%][e]	NA	NA	NA		
Any Infection **All studies**	6	24	80·1% [72·5-86%]	74·8% [66·0-81·9%]	68·6% [58·8-76·9%]	61·6% [51·2-71·1%]	46·5% [36·0-57·3%][e]	NA	NA	NA	-22·0 [-4·3 to -38·8]	NA
Any Infection **Moderate RoB only**	4	19	78·4% [66·0-87·2%]	72·2% [58·0-83·0%]	64·9% [49·6-77·6%]	61·6% [51·2-71·1%]	40·1% [26·1-55·9%][e]	NA	NA	NA	-	-

This table displays a sensitivity analysis for the point estimates and 95% CIs of protection shown in Figure 2. This analysis used a log-odds meta-regression model. [a]Month 1 data were for persons with hybrid immunity whose last immunological challenge was vaccination and thus were eligible for reinfection within a shorter time frame than people who most recently had prior infection (2 month minimum for probable reinfection). [b]Month 2 data represent the minimum time period for a reinfection among persons with prior infection (i.e., possible reinfection). [c]Confidence intervals calculated using the bootstrap method. Percentage point changes over time are reported from 3 months as this represents probable and confirmed reinfections. [d]Any infections contained mild infections, symptomatic infections and

asymptomatic infections [e]Model predictions beyond the range of the available data. NA: insufficient data for model extrapolation. Prior infection data is available for 2-16 month predictions; hybrid immunity data was available for 1-11 month predictions. Data were extrapolated to a maximum of 3 months beyond the final follow-up date. Abbreviations: RoB= risk of bias; N/A= not applicable.

Appendix 12. Sensitivity analysis of protection conferred by prior infection or hybrid immunity over time using the WHO definition of severe disease

a) Prior infection **b) Hybrid immunity (primary series vaccine)** **c) Hybrid immunity (booster)**

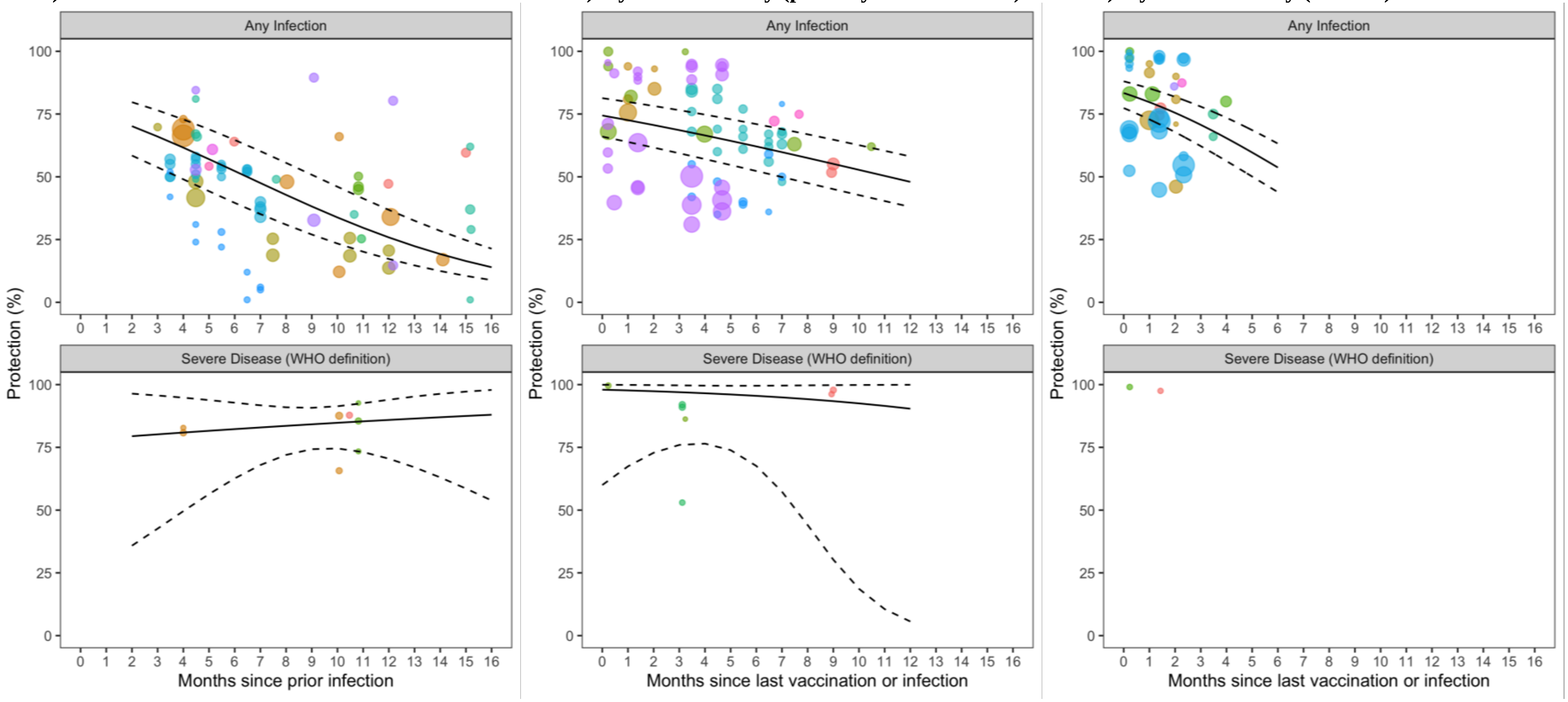

Points of the same color represent estimates from the same study. The diameter of points varies with the sample size of the study.

Appendix 13. Sensitivity analysis of the protection against reinfection and severe disease conferred by the primary-series vaccine, first booster vaccine, prior infection, and hybrid immunity compared to immune naive

Severity of Infection	No. studies	No. estimates	Month 1[a]	Month 2[b]	Month 3	Month 4	Month 6	Month 9	Month 12	Month 15	Percentage point change in protection from 3 to 6 months [95% CI][c]	Percentage point change in protection from 3 to 12 months [95% CI][c]
**Prior Infection**												
Severe Disease	3	8	NA	79·4% [35·8-96·4%]	80·2% [42·6-95·7%]	80·9% [49·6-94·8%]	82·3% [62·6-92·8%]	84·2% [74·3-90·7%]	85·9% [70·5-94%]	87·5% [58·6-97·2%]	-2·4 [-4·7 to +5·1]	-7·8 [-12·1 to +20·9]
Any Infection[d]	10	72	NA	70·2% [58·5-79·7%]	66% [53·8-76·5%]	61·6% [49-72·8%]	52·3% [39·6-64·7%]	38·2% [27-50·8%]	25·8% [17·3-36·8%]	16·4% [10·5-24·7%]	-14·0 [-12·0 to -18·2]	-40·5 [-33·9 to -51·9]
**Hybrid Immunity - Primary Series**												
Severe Disease	3	7	97·7% [67·4-99·9%]	97·4% [72·8-99·8%]	97% [76-99·7%]	96·6% [76·5-99·6%]	95·5% [67·6-99·5%]	93·4% [30·3-99·8%]	90·4% [5·6-99·9%]	NA	-1·5 [-3·4 to +11·5]	-6·6 [-20·9 to +17·8]
Any Infection	7	71	72·6% [63·9-79·8%]	70·6% [61·6-78·2%]	68·6% [59·4-76·5%]	66·5% [57-74·8%]	62·1% [52·3-71%]	55·1% [45·1-64·8%]	48·0% [38·0-58·1%]	NA	-6·5 [-15·1 to +4·9]	-20·6 [-48·8 to +11·4]
**Hybrid Immunity - First Booster**												
Severe Disease	2	2	98·2% [85·5-99·8%]	96·1% [17·4-100%]	NA	NA	NA	NA	NA	NA	NA	NA
Any Infection	6	39	79·7% [72·9-85·2%]	75·5% [67·8-81·8%]	70·7% [62·2-77·9%]	65·4% [56·3-73·5%]	53·7% [43·9-63·2%]	NA	NA	NA	NA	NA

This table displays the data shown in Figure S9. This analysis uses the same log-odds meta-regression model as Figure 2. [a]Month 1 data were for persons with hybrid immunity whose last immunological challenge was vaccination and thus were eligible for reinfection within a shorter time frame than people who most recently had prior infection (2 month minimum for probable reinfection). [b]Month 2 data represented the minimum time period for an infection among persons with prior infection [c]Confidence intervals calculated using the bootstrap method. [d]Any infection contained mild infections, symptomatic infections and asymptomatic infections. NA: insufficient data for model extrapolation. Prior infection data was available for 2-16 month predictions; hybrid immunity data was available for 1-11 month predictions. Data were extrapolated to a maximum of 3 months beyond the final follow-up date.

Appendix 14. Definitions of severe disease in included studies

First Author (Country)	Types of exposures	Sensitivity defined as severe, critical, or fatal COVID-19	Severe disease definition
Altarawneh 1 (Qatar)[9]	Prior infection	Yes	Severe, critical, or fatal COVID-19 as defined per the WHO classification.
Altarawneh 2 (Qatar)[10]	Prior infection and Hybrid immunity	Yes	Severe, critical, or fatal COVID-19 as defined per the WHO classification.
Andeweg (Netherland)[11]	Prior infection and Hybrid immunity	No	N/A
Björk (Sweden)[19]	Hybrid immunity	Yes	A case who was hospitalised for at least 24 h from 5 days before until 14 days after a positive SARS-CoV-2 test and required oxygen supply (≥ 5 L/min) or admittance to an ICU.
Bruel (France)[20]	Hybrid immunity	No	N/A
Carazo (Canada)[12]	Prior infection and Hybrid immunity	No	COVID-19 hospitalization, defined by admission, ≥24-hours and within 14 days of a SARS-CoV-2 positive specimen
		Yes	COVID-19 death
Cerqueira-Silva (Brazil)[13]	Prior infection and Hybrid immunity	No	N/A
Chin (USA)[14]	Prior infection and Hybrid immunity	No	N/A
Lind (USA)[15]	Prior infection and Hybrid immunity	No	N/A
Medić (Serbia)[21]	Prior infection and Hybrid immunity	Yes	COVID-19 pneumonia confirmed by chest imaging
		Yes	COVID-19 pneumonia required mechanical ventilation and/or admission to the ICU
Michlmayr (Denmark)[16]	Prior infection	No	Hospital admission associated with ICD-10 primary diagnosis codes occurring no earlier than two days before, and no later than 14 days after a positive RT-PCR test.
Nielsen (Denmark)[22]	Hybrid immunity	No	N/A
Nyberg (UK)[17]	Prior infection	No	Any hospital attendances, including admissions and attendances at accident and emergency departments, 0–14 days after the first specimen date of the most recent infection episode.
	Prior infection	No	Hospital attendances, admissions and diagnoses during hospital stay
		Yes	Death occurring 0–28 days after the first positive specimen date of the most recent infection episode, again matching the definition used in routine UK government reporting.
Plumb (USA)[23]	Hybrid immunity	No	At least one hospital admission for a COVID-19-like illness, with a hospitalization-associated NAAT performed from 10 days before through 3 days after admission.
		No	COVID-19-like illness: acute respiratory illness or related signs or symptomsusing diagnosis codes from the ICD-10
Shrestha (USA)[24]	Hybrid immunity	No	N/A
Šmíd (Czech)[18]	Prior infection and Hybrid Immunity	No	Hospital admission of a person, who tested positive on a PCR test, within two weeks after the confirmed infection or earlier
		Yes	Admission to ICU during the hospitalization.
		Yes	Use of any type of oxygen therapy

Appendix 15. Six-month protection against reinfection and severe disease conferred by the primary-series vaccine, first booster vaccine, prior infection, and hybrid immunity compared to immune naive individuals

Group	Number of studies	Six-month protection	p-value
**Hospitalization or severe disease**			
Primary series vaccine	12	64·6% [54·5-73·6%]	<0·0001
First booster vaccine	10	76·7% [72·5-80·4%]	<0·0001
Prior infection	6	80·1% [70·3-87·2%]	0·01
Hybrid immunity (primary series vaccine)	5	96·5% [90·2-98·8%]	*ref.*
Hybrid immunity (first booster)	4	95·3% [81·9-98·9%]	0·75
**Any infection**			
Primary series vaccine	15	15·1% [11·3-19·8%]	<0·0001
First booster vaccine	9	24·8% [18·5-32·5%]	<0·0001
Prior infection	10	51·2% [38·6-63·7%]	0·28
Hybrid immunity (primary series vaccine)	7	60·4% [49·6-70·3%]	*ref*
Hybrid immunity (first booster)	6	46·5% [36·0-57·3%]	0·08

Vaccine effectiveness raw data was obtained from a previous systematic review.[6] To obtain six-month protection, we ran a log-odds meta-regression model on all of the data allowing for different slopes and intercepts for each group and a random intercept for each study. We centered the month variable by subtracting six from the number of months such that the intercept for each group represents the protection at six months. See also Figure 3.

Appendix 16. Characteristics of vaccine effectiveness studies

Characteristic	Vaccine effectiveness studies
	Primary or booster vaccination vs. Immune-naive
	N = 19[a] n (%)
Study design	
Cohort	5 (26%)
Cross-sectional	0 (0%)
Test-negative design case-control	14 (74%)
Traditional case-control	0 (0%)
Vaccine-specific estimates	99
Primary series	48
Booster	51
Study population	
General population	18 (95%)
Healthcare workers	1 (5%)
WHO region	
AFR	1 (5%)
AMR	9 (47%)
EMR	1 (5%)
EUR	8 (42%)
SEAR	0 (0%)
WPR	0 (0%)
Reported the time interval since final dose (days)	19 (100%)
Primary series vaccination[b]	
Inactivated: Sinovac-CoronaVac	1 (5%)
mRNA:Pfizer/BioNTech-Comirnaty	16 (84%)
mRNA: Moderna-mRNA-1273	6 (32%)
mRNA: Pfizer/BioNTech-Comirnaty + Moderna-mRNA-1273	8 (42%)
NRVV: AstraZeneca-Vaxzevria	6 (32%)
NRVV: Janssen-Ad26.COV2.S	2 (11%)
Mixed (Inactivated + NRVV + mRNA)	0 (0%)
First booster vaccination[b]	
Inactivated: Sinovac-CoronaVac	1 (5%)
mRNA:Pfizer/BioNTech-Comirnaty	10 (53%)
mRNA: Moderna-mRNA-1273	8 (42%)
mRNA: Pfizer/BioNTech-Comirnaty + Moderna-mRNA-1273	6 (32%)
NRVV:AstraZeneca-Vaxzevria	0 (0%)
NRVV: Janssen-Ad26.COV2.S	1 (5%)
Mixed (Inactivated + NRVV + mRNA)	0 (0%)
Infection severity	
Any reinfection	17 (89%)
Severe disease (includes hospitalisation)	12 (63%)
Severe disease (as per WHO definition)	1 (5%)
Adjustment of primary estimate	
Population characteristics only	19 (100%)
Other characteristics (e.g., time, socioeconomic status, location)	16 (84%)
Risk of bias	
Moderate	16 (84%)
Serious	3 (16%)

[a]Characteristics of 19 studies from 18 articles were reported by the authors of the systematic review.(6) [b]One study presented estimates separately from two different countries and was counted twice. Abbreviations: WHO= World Health Organization; AFR=African Region; AMR=Region of the Americas; EMR=Eastern Mediterranean Region; EUR=European Region; SEAR=South-East Asian Region; WPR=Western Pacific Region; mRNA= messenger ribonucleic acid; NRVV=non-replicating viral vector.

Appendix 17. References for the supplement

1. CADTH COVID-19 Search Strings. CADTH Covid-19 Evidence Portal. Accessed September 2, 2022. https://covid.cadth.ca/literature-searching-tools/cadth-covid-19-search-strings/

2. World Health Organization. WHO COVID-19 Case definition - Updated in Public health surveillance for COVID-19. Published July 22, 2022. Accessed September 2, 2022. https://www.who.int/publications-detail-redirect/WHO-2019-nCoV-Surveillance_Case_Definition-2022.1

3. World Health Organization. *Living Guidance for Clinical Management of COVID-19*.; 2021. https://apps.who.int/iris/bitstream/handle/10665/349321/WHO-2019-nCoV-clinical-2021.2-eng.pdf

4. World Health Organization. *International Guidelines for Certification and Classification (Coding) of COVID-19 as Cause of Death.* https://www.who.int/publications/m/item/international-guidelines-for-certification-and-classification-(coding)-of-covid-19-as-cause-of-death

5. Higdon MM, Baidya A, Walter KK, et al. Duration of effectiveness of vaccination against COVID-19 caused by the omicron variant. *Lancet Infect Dis*. 2022;22(8):1114-1116. doi:10.1016/S1473-3099(22)00409-1

6. Szanyi J, Wilson T, Scott N, Blakely T. A log-odds system for waning and boosting of COVID-19 vaccine effectiveness. *Vaccine*. 2022;40(28):3821-3824. doi:10.1016/j.vaccine.2022.05.039

7. Zhang J, Yu KF. What's the Relative Risk?A Method of Correcting the Odds Ratio in Cohort Studies of Common Outcomes. *JAMA*. 1998;280(19):1690-1691. doi:10.1001/jama.280.19.1690

8. Altarawneh HN, Chemaitelly H, Hasan MR, et al. Protection against the Omicron Variant from Previous SARS-CoV-2 Infection. *N Engl J Med*. 2022;386(13):1288-1290. doi:10.1056/NEJMc2200133

9. Altarawneh HN, Chemaitelly H, Ayoub HH, et al. Effects of Previous Infection and Vaccination on Symptomatic Omicron Infections. *N Engl J Med*. 2022;387(1):21-34. doi:10.1056/NEJMoa2203965

10. Andeweg SP, de Gier B, Eggink D, et al. Protection of COVID-19 vaccination and previous infection against Omicron BA.1, BA.2 and Delta SARS-CoV-2 infections. *medRxiv*. Published online January 1, 2022:2022.02.06.22270457. doi:10.1101/2022.02.06.22270457

11. Carazo S, Skowronski DM, Brisson M, et al. Protection against Omicron re-infection conferred by prior heterologous SARS-CoV-2 infection, with and without mRNA vaccination. *medRxiv*. Published online January 1, 2022:2022.04.29.22274455. doi:10.1101/2022.04.29.22274455

12. Cerqueira-Silva T, de Araujo Oliveira V, Paixão ES, et al. Vaccination plus previous infection: protection during the omicron wave in Brazil. *Lancet Infect Dis*. 2022;22(7):945-946. doi:10.1016/S1473-3099(22)00288-2

13. Chin ET, Leidner D, Lamson L, et al. Protection against Omicron conferred by mRNA primary vaccine series, boosters, and prior infection. *medRxiv*. Published online January 1, 2022:2022.05.26.22275639. doi:10.1101/2022.05.26.22275639

14. Lind ML, Robertson AJ, Silva J, et al. Effectiveness of Primary and Booster COVID-19 mRNA Vaccination against Omicron Variant SARS-CoV-2 Infection in People with a Prior SARS-CoV-2 Infection. *medRxiv*. Published online January 1, 2022:2022.04.19.22274056. doi:10.1101/2022.04.19.22274056

15. Michlmayr D, Hansen CH, Gubbels SM, et al. Observed protection against SARS-CoV-2 reinfection following a primary infection: A Danish cohort study among unvaccinated using two years of nationwide PCR-test data. *Lancet Reg Health - Eur*. 2022;20:100452. doi:10.1016/j.lanepe.2022.100452

16. Nyberg T, Ferguson NM, Nash SG, et al. Comparative analysis of the risks of hospitalisation and death associated with SARS-CoV-2 omicron (B.1.1.529) and delta (B.1.617.2) variants in England: a cohort study. *The Lancet*. 2022;399(10332):1303-1312. doi:10.1016/S0140-6736(22)00462-7

17. Šmíd M, Berec L, Přibylová L, et al. Protection by Vaccines and Previous Infection Against the Omicron Variant of Severe Acute Respiratory Syndrome Coronavirus 2. *J Infect Dis*. Published online April 28, 2022:jiac161. doi:10.1093/infdis/jiac161

18. Björk J, Bonander C, Moghaddassi M, et al. COVID-19 vaccine effectiveness against severe disease from SARS-CoV-2 Omicron BA.1 and BA.2 subvariants – surveillance results from southern Sweden, December 2021 to March 2022. *Eurosurveillance*. 2022;27(18):2200322.

19. Bruel T, Pinaud L, Tondeur L, et al. SARS-CoV-2 Omicron neutralization and risk of infection among elderly after a booster dose of Pfizer vaccine. *medRxiv*. Published online January 1, 2022:2022.03.30.22273175. doi:10.1101/2022.03.30.22273175

20. Medić S, Anastassopoulou C, Lozanov-Crvenković Z, et al. Risk and severity of SARS-CoV-2 reinfections during 2020–2022 in Vojvodina, Serbia: A population-level observational study. *Lancet Reg Health - Eur*. 2022;20:100453. doi:10.1016/j.lanepe.2022.100453

21. Nielsen KF, Moustsen-Helms IR, Schelde AB, et al. Vaccine effectiveness against SARS-CoV-2 reinfection during periods of Alpha (B.1.1.7), Delta (B.1.617.2) or Omicron (B.1.1.529) dominance: A Danish nationwide study. *medRxiv*. Published online January 1, 2022:2022.06.01.22275858. doi:10.1101/2022.06.01.22275858

22. Plumb I, Feldstein L, Barkley E, et al. Effectiveness of COVID-19 mRNA Vaccination in Preventing COVID-19–Associated Hospitalization Among Adults with Previous SARS-CoV-2 Infection — United States, June 2021–February 2022. *2022*. 71:549-555.

23. Shrestha NK, Shrestha P, Burke PC, Nowacki AS, Terpeluk P, Gordon SM. Coronavirus Disease 2019 Vaccine Boosting in Previously Infected or Vaccinated Individuals (COVID-19). *Clin Infect Dis*. Published online April 27, 2022:ciac327. doi:10.1093/cid/ciac327
